# Advances in nanomaterial-mediated CRISPR/Cas delivery: from lipid nanoparticles to vesicle-derived systems

**DOI:** 10.3389/fbioe.2025.1669104

**Published:** 2026-01-22

**Authors:** Bingning Wang, Jingyuan Lu, Xiaoyi Zhang, Ruoyang Hu, Haowei Ma

**Affiliations:** 1 Department of Chemical and Biomolecular Engineering, Institute of Materials Science, University of Connecticut, Storrs, CT, United States; 2 Tongji Medical College, Huazhong University of Science and Technology, Wuhan, China; 3 Department of Medicine, Jacobi Medical Center/Albert Einstein College of Medicine, Bronx, NY, United States; 4 Sargent College, Boston University, Boston, MA, United States; 5 Department of Mechanical and Aerospace Engineering, Case Western Reserve University, Cleveland, OH, United States

**Keywords:** nanocarriers, CRISPR/Cas delivery, gene editing, lipid nanoparticles, polymeric nanoparticles, targeted delivery, biocompatibility, *in vivo* gene therapy

## Abstract

Gene and genome editing therapies are increasingly connected with nanomaterials, which protect and transport fragile nucleic acids and CRISPR/Cas systems through biological barriers safely and accurately. This review discusses how different nanocarriers, including lipid-based, polymeric, inorganic, and vesicle-derived systems, can improve delivery efficiency, cell targeting, endosomal escape, and intracellular movement for gene and genome editing. It summarizes findings from early clinical and preclinical studies, comparing several carrier types such as ionizable lipid nanoparticles, polymeric nanoparticles, micelles, gold and silica nanostructures, and engineered extracellular vesicles. The review also explains how specific design factors, such as surface ligands, charge modification, PEGylation, and stimulus-responsive behaviors, influence biodistribution, and improve on-target efficiency while lowering immune responses and off-target effects. Ethical and regulatory concerns for *in vivo* editing are highlighted, along with current methods used to study nano–bio interactions. Among these carriers, ionizable lipid nanoparticles show the most advanced performance for delivering nucleic acids and CRISPR systems. However, new polymer-based and exosome-inspired carriers are progressing rapidly for repeated and targeted applications. Hybrid and responsive systems may also enable better spatial and temporal control of editing. Future research should focus on stronger *in vivo* potency testing, improved biocompatibility evaluation, and standardized manufacturing to ensure clinical safety and reliability.

## 1 Introduction

In genetic medicine, the integration of nanomaterials with gene and genome editing has enabled a major breakthrough, creating new possibilities for improving therapeutic strategies (Mostafavi and Zare, 2022). Over the past few decades, deeper understanding of the human genome has enabled the development of new methods to treat genetic disorders (Zhang W. et al., 2021). The use of nanomaterials has become a key innovation in this field, improving the precision, efficiency, and safety of gene therapy and genome editing (Yadav K. et al., 2023). Their unique physical and chemical properties make them suitable for developing more specific and targeted treatments (Liu L. et al., 2021), as they can interact directly with genetic material and influence how genes are controlled and expressed (Kim et al., 2018; Priyadarsini et al., 2018). Gene therapy, which involves adding, deleting, or modifying genes within human cells, offers hope for treating inherited diseases and certain cancers (Mirón-Barroso et al., 2021; Gong et al., 2023). However, its success depends on creating delivery systems that can transfer therapeutic genes safely and effectively to the target cells (Rizeq et al., 2019). Due to their adjustable size, surface charge, and high biocompatibility, nanomaterials have emerged as powerful carriers that help overcome these delivery challenges (Nocito et al., 2021).

Many types of nanomaterials have been explored to improve gene delivery, with each system providing distinct advantages (Dasari Shareena et al., 2018). Nanoparticles made from polymers or lipids can effectively encapsulate genetic material and protect it from enzymatic degradation (Nocito et al., 2021). Liposomes, composed of lipid bilayers resembling cellular membranes, facilitate cellular uptake by mimicking natural membrane structures (Jahangiri-Manesh et al., 2022). Engineered viral vectors also play a critical role in introducing therapeutic genes into target cells (Wei Hu et al., 2023). These nanomaterials not only safeguard the genetic cargo but also enhance intracellular transport, ensuring precise delivery to the intended site (Fernando et al., 2020). Meanwhile, CRISPR-based genome editing has revolutionized precision medicine (Gong et al., 2023). The CRISPR/Cas9 system allows for site-specific DNA modification with remarkable accuracy, offering strong potential for correcting genetic mutations and treating diseases at their molecular roots (Krasteva and Georgieva, 2022). However, the efficient delivery of CRISPR components to target cells remains a significant challenge (Shishparenok et al., 2023). Nanomaterials are essential in addressing this issue, as they can transport and protect CRISPR elements while guiding them to the appropriate genomic locations (Shi et al., 2014). Integrating nanomaterial-based carriers with CRISPR technologies enhances both editing precision and biosafety, advancing the practical use of these transformative tools (Hu et al., 2022).

The purpose of this research is to closely analyze the relationship between nanomaterials, gene therapy, and genome editing, focusing on current advances and potential future applications. The study mainly aims to explain how nanomaterials enhance the efficiency, precision, and safety of gene-based treatments and genome modification techniques. Various nanomaterial delivery systems, including nanoparticles, liposomes, and viral vectors, are examined to describe their functions in gene transfer, CRISPR-mediated genome editing, and RNA interference. Moreover, the review discusses the challenges associated with the use of nanomaterials in gene therapy, such as regulatory limitations and ethical concerns. It also identifies new developments and areas requiring further investigation. Overall, this study contributes to the expanding field of nanomaterial-based genetic medicine and supports a deeper understanding of their potential in treating genetic disorders.

## 2 Nanomaterial properties and functionalities

**FIGURE 1:**
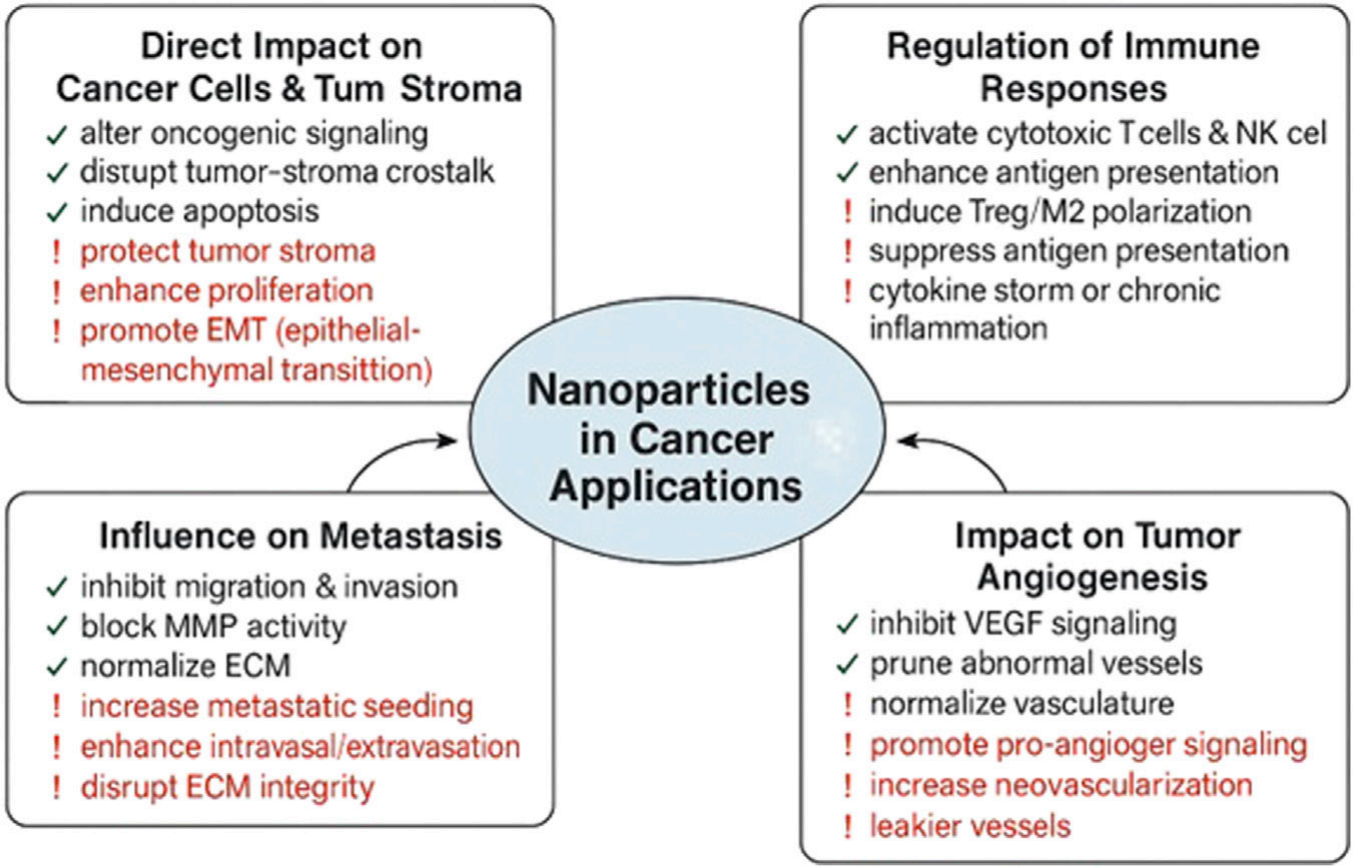
Roles of nanoparticles (NPs) in cancer. NPs can impede (green) or promote (red) cancer progression by directly affecting tumor cells and stroma, modulating immune responses, influencing metastasis, and altering angiogenesis.

Engineered nanomaterials play a crucial role in enhancing the diagnosis and treatment of diseases by addressing the limitations of conventional drug delivery systems (Fernando et al., 2020). Nanotechnology introduces innovative strategies, including the targeting of specific cells and directing therapeutic molecules to designated organelles (Jahangiri-Manesh et al., 2022). These advances improve the precision of drug delivery and help resolve major challenges related to biodistribution and intracellular transport (Raya et al., 2022). In 2000, the U.S. National Science and Technology Council initiated the National Nanotechnology Initiative (NNI), which outlined strategic objectives and challenges to stimulate progress and promote the development of advanced nano-enabled technologies (Boverhof et al., 2015). Nanoparticles (NPs) demonstrate significant potential by enhancing cargo stability and solubility, facilitating membrane transport, and prolonging circulation time, which together contribute to safer and more effective therapies (Figure 1). Despite extensive research, a considerable gap remains between preclinical animal results and human clinical applications, primarily due to limited understanding of physiological and pathological differences (Judith and Vasudevan, 2022). Bridging this gap is essential for realizing the full clinical potential of nanomedicine (Kumar et al., 2021; Stater et al., 2021).

The surface functionality of nanomaterials is crucial for targeted gene delivery in gene therapy, as it enables specific and controlled interactions with target cells (Anwar et al., 2020). In this process, nanoparticles are functionalized with ligands that selectively bind to receptors overexpressed on the surface of target cells, ensuring accurate cellular uptake and the precise delivery of therapeutic genes (Wang and Guo, 2016; Kreyling et al., 2010; Dufresne, 2017). When ligands possess high affinity for their receptors, nanomaterials can efficiently navigate the biological environment and reach the desired cells or tissues, hence minimizing off-target effects and enhancing therapeutic outcomes (Liu et al., 2022a; Ying et al., 2017). Surface modification thus allows researchers to customize ligands according to cellular characteristics, improving the specificity and efficiency of gene delivery systems (Manimekala et al., 2022). One of the greatest challenges in gene therapy is overcoming biological barriers such as the blood–brain barrier (BBB), which tightly regulates the movement of substances from the bloodstream into the brain (Kushwaha et al., 2022; Liu et al., 2022b; Chen et al., 2019). To address this, nanomaterials are engineered with surface ligands that interact with specific receptors or transport systems on BBB endothelial cells, facilitating receptor-mediated translocation into brain tissue (Chi and Liu, 2023; Xu and Liang, 2020; Ge et al., 2019). Careful selection and engineering of ligands remain essential, as they determine binding affinity, transport efficiency, and overall success in crossing complex biological barriers (Hao et al., 2022; Quader and Kataoka, 2017).

Surface functionality plays a crucial role in maintaining the stability of nanomaterials within complex biological environments, as it determines how these materials interact with biological components, particularly the immune system, which can strongly influence their circulation time and efficiency in gene delivery (Brüngel et al., 2023; Cheng et al., 2023). To improve their stability and biocompatibility, nanoparticles are often modified through surface coatings and charge adjustments (Vaidh et al., 2022). Coating nanoparticles with materials such as polymers or lipids forms a protective layer that prevents immune recognition and reduces *opsonization*, a process in which blood proteins adhere to the nanoparticle surface and mark them for clearance by phagocytic cells (Nain et al., 2022; Kladko et al., 2021). Minimizing opsonization extends the circulation time of nanomaterials, allowing them more opportunities to reach target cells and effectively deliver therapeutic genes (Liu MY. et al., 2021). Adjusting the surface charge also affects nanoparticle–biological interactions; neutral or slightly negative surfaces are preferred because they decrease nonspecific interactions and improve the “stealth” properties of the nanomaterials, thus enhancing their systemic stability and functional lifespan (Chen et al., 2019; Cheng et al., 2023; Liu et al., 2020). Furthermore, ligand selection is a key factor in achieving cell-specific targeting during gene delivery, as nanoparticles are engineered with surface ligands that can recognize and bind to overexpressed receptors on target cells (Rao et al., 2009; Liang T. et al., 2022). The process begins by characterizing the target cells and identifying receptors of interest, followed by selecting ligands with high affinity for those receptors (Yao et al., 2018; Jackman et al., 2021). This targeted recognition facilitates efficient cellular uptake, as the binding between the ligands and receptors triggers internalization mechanisms that enable the nanoparticles to enter the desired cells and deliver therapeutic genes (Huang et al., 2023; Buschmann et al., 2021). The specificity achieved through careful ligand selection helps minimize off-target effects by ensuring that nanoparticles interact only with intended cells, thereby reducing undesired biological responses and enhancing overall therapeutic efficacy (Bora et al., 2019; Yaqoob et al., 2020; Rawtani et al., 2019).

A biopolymer–Ni/Zn Np biocomposite (Prakasha et al., 2023) was developed using the exopolysaccharide of *Rhodotorula mucilaginosa* UANL-00IL, which facilitated the formation of polymorphic nanoparticles of 8–26 nm in size (Rahman et al., 2022). Unlike conventional nanocomposites with defined geometries, this material lacked a fixed structural shape, contributing to its unique physicochemical properties (Liang and Zhao, 2021). It demonstrated strong antimicrobial and antibiofilm activities against *Staphylococcus aureus* and *Pseudomonas aeruginosa* and *in vivo* assessments in male rats confirmed its biocompatibility, showing no toxicity at a dose of 24 mg/kg body weight (Rezk et al., 2020). Similarly, the AgNps/ZnONps nanocomposite was synthesized using an aqueous extract of the green alga *Ulva fasciata*, producing silver and zinc oxide nanoparticles of varying morphologies and dimensions (He et al., 2019). This composite exhibited significant antibacterial potential against *Escherichia coli* and *Salmonella* spp., particularly at elevated concentrations, and showed a synergistic effect when combined with antibiotics, suggesting its applicability in combination antimicrobial therapies (Raza et al., 2021; Vance et al., 2015). Another notable material, the tungsten carbide (Wc), silver (Ag), and copper (Cu) nanocomposite, was fabricated using commercially obtained nanoparticles of differing shapes and sizes (Chu et al., 2017). The mixture displayed potent antimicrobial efficiency against *S. aureus* and *P. aeruginosa*, confirming the collective role of its metallic components in enhancing bactericidal properties (Makabenta et al., 2021). Additionally, Ag/TiO_2_ nanorods were synthesized using the horizontal vapor-phase growth (HVPG) technique (Fu et al., 2022). Their needle-like ends were capable of penetrating bacterial membranes, which substantially increased their antibacterial performance against *S. aureus* by causing direct structural disruption of the cell wall (Lin and Wang, 2015).

**FIGURE 2:**
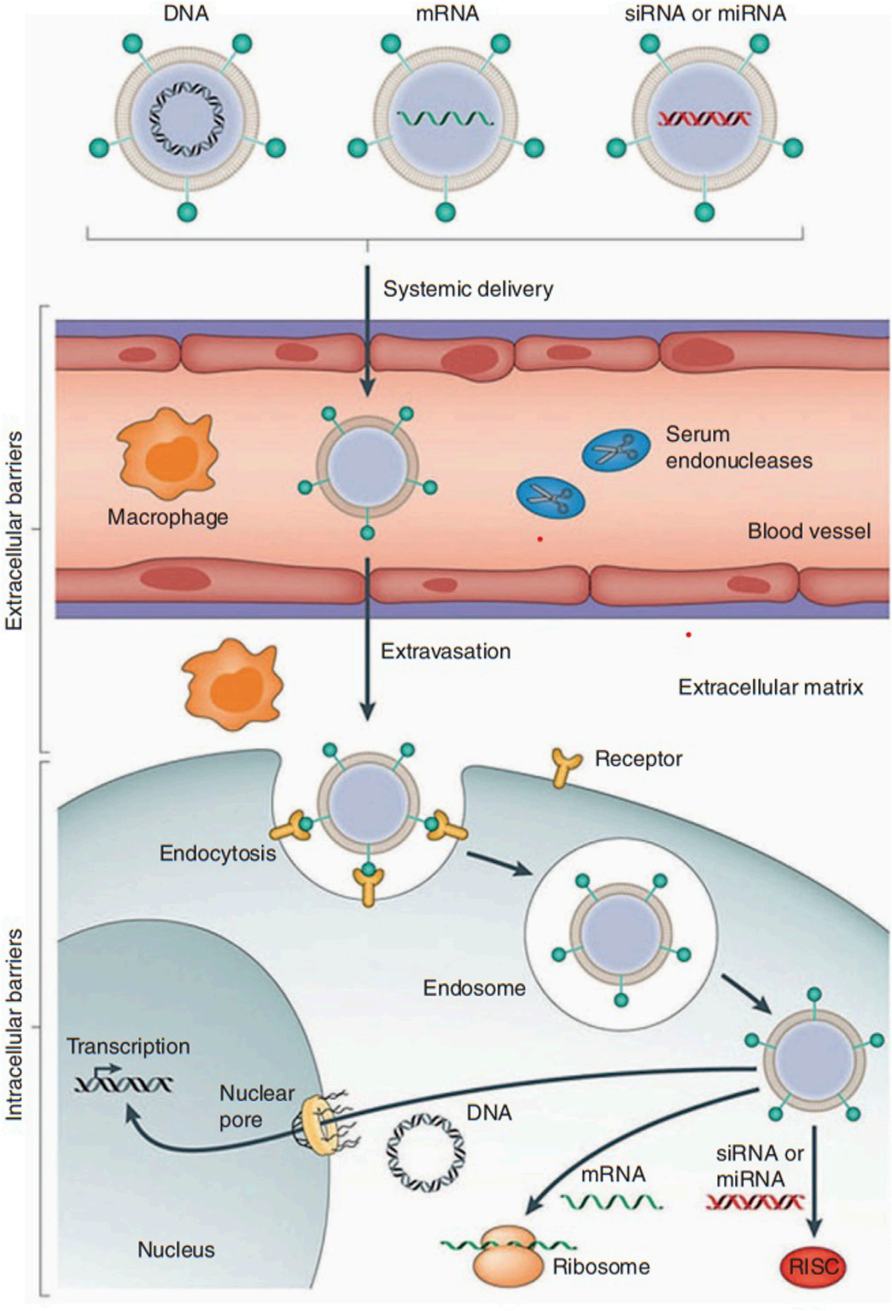
A nanoparticle for gene therapy. Reprinted from Chen et al. (2016) with permission from Springer Nature.

Another nanostructure, the Ag–Au/ZnO nanostructure, was synthesized with *Justicia adhatoda* plant extract (Karthikeyan et al., 2021). It demonstrated good antimicrobial activity against *E. coli* and *S. aureus*, showing the value of plant-based nanocomposites (Jones and Grainger, 2009). The α-BiO_2_-ZnO nanostructure, made by chemical synthesis, produced a 1.5-cm inhibition zone against *S. aureus* at 1 mg/L. T-β-D-glu-ZnO Nps (trichoderma-β-D-glucan-zinc oxide nanoparticles) were spherical in shape and synthesized with a fungal mycelial water extract from *Trichoderma harzianum* (Tsoi et al., 2016). They inhibited the growth of *S. aureus* inside roundworms and also showed dose-dependent inhibitory effects on human pulmonary carcinoma A549 cells (Shin et al., 2023). ZnO/Fe_3_O_4_/rGO nanocomposites were rod-shaped and spherical (Besenhard et al., 2023). They showed stronger effects against *E. coli* than *S. aureus*, and adding reduced graphene oxide (rGO) increased antibacterial activity (Irshad et al., 2023). A second type of Ag/TiO_2_ was made with an aqueous extract of *Acacia nilotica* (Zhao et al., 2023). This nanocomposite produced inhibition zones of different sizes against *E. coli*, MRSA, and *P. aeruginosa* (Teleanu et al., 2019). Its mechanism included lowering glutathione levels, which caused ROS production and lipid peroxidation (Eichhorn et al., 2022). Finally, ZEO-Ag Nps, ZEO-Cu Nps, and ZEO-Zn Nps were coatings made from copper-doped chitosan–gelatin (CSG) nanocomposites prepared by green synthesis (Yan et al., 2020). Their antibacterial activity depends on copper levels, and tests showed no harm to bone marrow stromal cell functions on Cu-doped coatings. This improved the biological performance of Ti-based materials (Bokov et al., 2021). Surface charge modification is an important factor in how nanomaterials work in targeted gene delivery (Sohaebuddin et al., 2010). The charge of nanoparticles influences how they interact with biological systems, including circulation time and uptake by cells (Besenhard et al., 2023). By changing the surface charge, scientists can improve their performance for delivering therapeutic genes (Lee et al., 2020). Neutral or slightly negative charges are usually preferred for targeted gene delivery (Mohsin et al., 2023). This helps reduce unwanted interactions with proteins or cells in the blood, giving nanomaterials more time to reach target cells (Mohsin et al., 2023; Xie et al., 2019). Surface charge also affects cellular uptake (Min et al., 2021). By lowering electrostatic repulsion between negatively charged cell membranes and the nanoparticles, positively or neutrally charged surfaces can improve uptake (Ashish and Singh, 2021). This is important for gene delivery because higher cellular uptake raises the chance of successful delivery of therapeutic material (Kuhlbusch et al., 2018). Biodegradation is another challenge in nanoparticle gene therapy since therapeutic nucleic acids can break down before they reach target cells (Kim et al., 2022), reducing their effectiveness (Kabir et al., 2019). To solve this, researchers use stable and biocompatible materials, such as lipid-based nanoparticles or polymer carriers (Sethuram et al., 2022). These materials protect the genetic cargo from enzymes, keeping it stable during circulation and helping deliver it to the right tissues (Figure 2).

## 3 Advances in gene-based therapies

Nanomaterials can encapsulate therapeutic genes, protecting them from degradation and facilitating their controlled release (Naskar and Kim, 2020). Additionally, surface modifications of nanomaterials allow for improved cellular uptake and intracellular trafficking of therapeutic genes (Jeong et al., 2022). This targeted and controlled delivery helps optimize the therapeutic effect while minimizing potential side effects (Nafees et al., 2013). Moreover, nanomaterials can overcome physiological barriers such as the blood–brain barrier, enabling the delivery of gene therapies to previously inaccessible tissues (Sharma et al., 2022). These advances in delivery technology open new possibilities for treating diseases with a genetic component that were previously challenging to effectively address (Alamri et al., 2020). One example of nanomaterial-enhanced gene therapy is the use of liposomal nanoparticles (Pokhrel et al., 2016). These lipid-based nanomaterials have demonstrated success in delivering therapeutic genes, especially for treating diseases like cancer (Sheikhzadeh et al., 2021). Liposomal nanoparticles can encapsulate genes, protect them during circulation, and release them specifically at the target site, maximizing the therapeutic effect while minimizing damage to healthy tissues (Kishore et al., 2023). Multifunctional nanoparticles play a crucial role in preclinical gene delivery studies, offering a versatile platform for enhancing the efficiency and specificity of gene targeting (Zhang Y. et al., 2021). One key strategy in nanoparticle functionalization involves PEGylation, which employs entities such as PEG-βCD and PEG-PEI (Stern et al., 2012). This approach contributes to enhanced stabilization, prevents unwanted protein absorption, and extends circulation time, thereby optimizing the overall performance of gene delivery systems (Han et al., 2022). Another significant aspect of multifunctional nanoparticles lies in their targeting capabilities (Yin et al., 2017). Utilizing constructs such as RGD-HA-PEI-PBLG, R-PEG20C, and transferrin-coated lipid, researchers have achieved improved gene target efficacy *in vivo* (Yang et al., 2015). These targeted delivery systems enable a more precise and efficient approach, enhancing the therapeutic impact of gene delivery (Wang et al., 2018). Stimulus-responsive nanoparticles further enhance gene delivery efficacy (Yang TC. et al., 2020). Through pH-sensitive, light-sensitive, and redox-sensitive designs, these nanoparticles respond dynamically to the microenvironment, ensuring optimal gene delivery under specific conditions (Pillai, 2014). This adaptability enhances the overall success of gene delivery systems *in vivo* (Rawtani et al., 2019). Cell-penetrating nanoparticles, such as p (DAHa-E/APIb), exhibit the ability to cross cell membranes efficiently, facilitating enhanced cellular uptake (Unnisa et al., 2023). This property is vital for ensuring the effective delivery of genetic material into target cells, thereby maximizing the therapeutic potential of gene-based therapies (Yang Y. et al., 2020). In the context of endosome escaping, nanoparticles like (Arg)7-FI-PNA have demonstrated the ability to cross cell membranes and improve endosomal escaping, overcoming a significant barrier in the gene delivery process (Pillai and Ceballos-Coronel, 2013). This capability is critical for ensuring that the genetic material reaches its intended destination within the cell (Wong et al., 2023). Nuclear localization is another key consideration, and nanoparticles such as PC/pDNA/NLS and VKRKKKP-R8 have been designed to facilitate it (Samarasinghe et al., 2012). This capability ensures that the delivered genetic material reaches the cell nucleus, where it can exert its therapeutic effects more effectively (Åström et al., 2015). Table 1 gives a detailed summary of gene-based therapies that use nanomaterials to treat different diseases. One clear example is the use of polymeric nanoparticles in delivering gene therapies for genetic disorders (Zheng et al., 2021). These nanoparticles, made of biocompatible and biodegradable materials, have been shown to deliver therapeutic genes effectively to target cells (Vimbela et al., 2017). In Duchenne muscular dystrophy (DMD), a serious genetic disease, researchers have used polymeric nanoparticles to carry the dystrophin gene. This method restored gene expression and reduced disease symptoms in preclinical studies (Yuan et al., 2023). Another important example is the use of viral vectors covered with nanomaterials to improve their safety and efficiency (Flores et al., 2012). Adeno-associated virus (AAV), which is often used in gene therapy, has been coated with nanomaterials to increase stability and improve targeting (Suri et al., 2007). This improved AAV has shown good results in treating inherited retinal diseases by delivering therapeutic genes to the retina, slowing the loss of vision (Zhi et al., 2022). Lipid-based nanocarriers are also important in developing cancer gene therapies (Kavanagh and Green, 2022). For example, liposomal nanoparticles have been used to deliver tumor-suppressing genes directly to cancer cells (Sahel et al., 2023). This targeted approach reduces harm to healthy tissues and increases therapeutic effects, highlighting the value of nanomaterials in precision medicine for cancer treatment (Demirer et al., 2021).

**TABLE 1:** Examples of gene-based therapies utilizing nanomaterials for various diseases.

Disease/condition	Type of gene therapy	Nanomaterial carrier	Therapeutic effect	Company/institution	Reference
Hereditary transthyretin amyloidosis (hATTR)	RNA interference (siRNA)	Lipid nanoparticles (LNPs)	Reduces transthyretin protein production	Alnylam Pharmaceuticals	Kaur et al. (2023)
Cytomegalovirus (CMV) retinitis	Viral vector (adenovirus)	Polymeric nanoparticles	Delivers antiviral genes to infected cells	Emory University	Padayachee and Singh (2020)
Cancer (melanoma)	DNA plasmid	Polymeric nanoparticles	Delivers tumor-suppressor genes	University of California, Los Angeles	Kopecny and Vlcko (2020)
Hemophilia B	Viral vector (adeno-associated virus)	Lipid nanoparticles (LNPs)	Corrects the factor IX gene defect	Pfizer	Gad et al. (2020)
Beta-thalassemia	Lentiviral vector	Modified red blood cells	Corrects the beta-globin gene defect	National Institutes of Health	Li et al. (2023)
Parkinson’s disease	AAV vector	Focused ultrasound	Delivers genes to regulate dopamine production	The Michael J. Fox Foundation	Xie et al. (2021)
Lysosomal storage disorders	AAV vector	Engineered exosomes	Delivers enzyme replacement genes to target cells	Massachusetts Institute of Technology	Salekdeh et al. (2021)
Cystic fibrosis	AAV vector	Nebulized liposomes	Corrects the *CFTR* gene defect in lung cells	Vertex Pharmaceuticals	Hur and Chung (2021)
Huntington’s disease	CRISPR/Cas9	AAV vector	Silences the mutated huntingtin gene	University College London	Shao et al. (2021)
Sickle cell disease	Lentiviral vector	Modified hematopoietic stem cells	Corrects the beta-globin gene defect	Bluebird Bio	Salman et al. (2022)
Age-related macular degeneration (AMD)	AAV vector	Injectable nanoparticles	Delivers genes to regulate vascular endothelial growth factor (VEGF)	Harvard University	Živojević et al. (2021)
Type 1 diabetes	AAV vector	Pancreatic islet cells	Restores insulin production	University of California, San Francisco	Song et al. (2023)
Alpha-1 antitrypsin deficiency	AAV vector	Liposomes	Delivers the alpha-1 antitrypsin gene to liver cells	Takeda Pharmaceutical Company	Falzarano et al. (2015)
Glioblastoma (brain cancer)	Plasmid DNA	Polymeric nanoparticles	Delivers tumor-suppressor genes and immune-stimulatory genes	The Ohio State University	Miller and Siegwart (2018)
Duchenne muscular dystrophy (DMD)	AAV vector	Microfluidic chip delivery	Delivers microdystrophin gene to muscle cells	Stanford University	Lastochkina et al. (2022)
Myocardial infarction (heart failure)	Plasmid DNA	Exosomes	Delivers pro-angiogenic genes to stimulate blood vessel growth	The Texas Heart Institute	Wu et al. (2022a)

One of the main ways nanomaterials reduce off-target effects is by surface modifications that improve cellular specificity (Xi et al., 2022). When nanomaterial surfaces are functionalized with ligands or peptides, they can selectively bind to specific cell receptors, directing the nanoparticles to the intended target cells (Mohammadinejad et al., 2020). This targeted process lowers the chance of therapeutic genes affecting non-target cells, thereby reducing off-target effects (Mohamad et al., 2023). In addition, the size and physicochemical properties of nanomaterials help them avoid the immune system and reach the target cells more effectively (Liu et al., 2023). Encapsulating therapeutic genes within nanocarriers protects them from degradation and immune detection during circulation (Xin et al., 2022). This protection ensures that the therapeutic payload remains intact until it arrives at the target site, thus reducing the possibility of off-target interactions (Duan et al., 2021). Nanomaterials also provide controlled release systems, which allow a gradual and continuous delivery of therapeutic genes (He and Zhao, 2020). Such controlled release is important because it prevents sudden increases in gene expression that might otherwise cause off-target effects (Tariq et al., 2023). By controlling release kinetics, nanomaterials help maintain a therapeutic concentration at the target site while reducing exposure in non-target tissues (Hamid and Salee, 2022). In addition, nanomaterials can cross physiological barriers, such as the BBB, which improves their specificity for particular tissues (Yang et al., 2022). This property is very important in gene therapies for diseases of the central nervous system since off-target effects may cause serious problems (Zhang L. et al., 2022). The BBB is a natural barrier that limits the entry of foreign substances, including therapeutic agents, into the brain (Höijer et al., 2020). Although it is essential for keeping the central nervous system stable, the BBB also creates a major obstacle to delivering gene therapies to treat neurological diseases (Dodds, 2023). Nanomaterials can overcome this obstacle through several mechanisms (Hossain A. et al., 2021). One common method is to modify the surface of nanoparticles with ligands or peptides that can attach to receptors on BBB endothelial cells (Zhen et al., 2020). These modified nanoparticles are able to bind to the receptors, cross the BBB by transcytosis, and deliver therapeutic genes into the brain parenchyma (Foley et al., 2022). This strategy increases the specificity of gene delivery and reduces off-target effects in other tissues (Saritha et al., 2022). The small size of nanomaterials is very important for their ability to pass through the tight junctions of the BBB (Cho et al., 2019). Nanoparticles can use endocytosis and transcytosis pathways to cross the BBB effectively, which makes it possible to deliver therapeutic genes into the brain (Aziz et al., 2023). This feature is highly useful for treating neurodegenerative diseases and genetic disorders that affect the central nervous system (Campuzano and Pingarrón, 2023). In addition, nanocarriers help protect therapeutic genes while they circulate in the body (Huang et al., 2021). The encapsulation of genes inside nanomaterials protects them from enzymatic breakdown and immune system detection, keeping them safe and functional when they reach the target site in the brain (Shende and Trivedi, 2021). One of the main functions of liposomal nanoparticles is their capacity to enclose and protect therapeutic genes (Foley et al., 2022). The lipid bilayer of liposomes forms a safe environment around the gene cargo, preventing enzymatic breakdown and avoiding immune detection during circulation in the body (Chen et al., 2020). This protective role helps ensure the stable and efficient delivery of therapeutic genes to the tumor site (Yang et al., 2022). In addition, the surface properties of liposomal nanoparticles can be adjusted to improve their selectivity toward cancer cells (Sharma and Lew, 2022). By adding targeting ligands or antibodies, they can specifically recognize and attach to receptors that are highly expressed on the surface of cancer cells (Satta et al., 2023). This targeted strategy allows greater build-up of liposomal nanoparticles inside tumor tissue, which improves therapeutic impact while reducing harm to normal healthy cells (Lee et al., 2022). Liposomal nanoparticles can also provide the controlled release of therapeutic genes, which allows for longer exposure to cancer cells (Yan and Liang, 2022). This slow release is important because it helps keep an effective number of therapeutic genes in the tumor microenvironment, thus improving treatment results (Liang and Liang, 2021). In addition, liposomal nanoparticles are flexible and can deliver more than one therapeutic agent, such as genes together with chemotherapy drugs (Crowley et al., 2021). Using this combined strategy increases the success of cancer treatment by targeting different parts of tumor growth and development at the same time (Chin et al., 2019). Progress in gene-based therapies has also changed personalized medicine, making it possible to design treatments that match the genetic background of each patient (Zhang A. et al., 2022). A major benefit of gene-based therapies in personalized medicine is that they can address genetic disorders at their source (Tiwari et al., 2023). Instead of only managing symptoms, these treatments focus on the genetic mutations or problems that cause the disease (Li et al., 2022a). This approach provides more effective and lasting results, which fits with the main ideas of personalized medicine (Liang Y. et al., 2022). Gene therapies also make it possible to design treatments based on each person’s unique genetic profile (Abinaya and Viswanathan, 2021). By studying a patient’s genetic data, doctors can find specific markers or mutations that play a role in the development of disease (Gbian and Omri, 2021). Tailoring gene therapies to focus on these specific genetic factors can improve the effectiveness of treatment while reducing possible side effects, creating a more personalized type of patient care (Souri et al., 2022). In addition, progress in gene editing tools, especially CRISPR/Cas9, has introduced new opportunities for accurate genome changes (Lu et al., 2023). This progress makes it possible to fix harmful genetic mutations or add therapeutic genes directly into the patient’s genome (Rouatbi et al., 2019). Editing genes at the molecular level increases the value of personalized medicine because it addresses the exact genetic causes of diseases (Evans et al., 2019). The use of gene-based therapies is not limited to genetic disorders but also extends to cancer treatment (Moradi et al., 2022). In this case, targeted gene therapies for cancer can use the unique genetic features of tumor cells, giving a specific approach that avoids unnecessary harm to healthy tissues (Wu Z. et al., 2022). Such targeted accuracy is a main feature of personalized medicine, where treatment plans are created based on the genetic and molecular profile of each individual (Dinari et al., 2018).

### 3.1 Precision genome editing techniques

Nanomaterials have an important role in improving precision genome editing because they act as carriers for different gene-editing tools (QIAO et al., 2021). They are usually designed as nanoparticles and display special features such as large surface area, good biocompatibility, and the possibility of surface modification (Chenouard et al., 2023). These properties make nanomaterials, such as gold nanoparticles, suitable for delivering CRISPR/Cas9 components with accuracy, which supports efficient and targeted changes in the genome (Khan, 2021). Their function in protecting and transporting genetic material also increases the success of precision genome editing methods (Sun et al., 2023). Many types of nanomaterials are useful in this field, including liposomes, polymers, and inorganic nanoparticles such as gold and silica (Khan, 2021). Liposomes—made of lipid bilayers—can encapsulate and transport genetic material, while biodegradable polymers are helpful for the controlled release of editing tools (Sun et al., 2023). Inorganic nanoparticles like gold and silica give stability and controlled release because of their tunable surface chemistry and porous structure (Ojha et al., 2023). Nanomaterials thus improve the outcomes of genome editing applications (Tay and Melosh, 2019; Wang et al., 2025). CRISPR technology, which uses RNA-guided Cas proteins, has changed the field of precision genome editing (Ryu et al., 2020). It gives researchers the ability to edit genes precisely, either to correct mutations or add specific changes (Shen Y. et al., 2018). Despite being simple, cost-effective, and versatile, CRISPR still faces challenges such as off-target effects and ethical issues, which have pushed researchers to improve it further (Li et al., 2017). Although CRISPR technology is the leading tool, other genome editing methods like zinc finger nucleases (ZFNs) and transcription activator-like effector nucleases (TALENs) are also available (Wei et al., 2022). Both ZFNs and TALENs are protein-based systems that create double-strand breaks for editing (Yang et al., 2023). However, compared to CRISPR, they are usually more complex and less flexible in their design (Li, 2019). The ease of use and versatility of CRISPR make it the most common choice for precise genome editing, though scientists continue to work on making it more accurate and on reducing unwanted effects (Zhai et al., 2022).

Nanomaterials play an important role in delivering CRISPR components by encapsulating and protecting genetic material until it reaches target cells (Alsulami, 2021). For example, lipid-based nanocarriers form stable complexes with CRISPR, protecting them from degradation and helping cellular uptake (Zia et al., 2021). The surface properties of nanomaterials can also be designed to improve targeting, reduce off-target effects, and increase precision in genome editing (Peng et al., 2020). Researchers are still improving nanomaterials to make delivery safer and more effective (Gao et al., 2021). By modifying surfaces and adding functional groups, they aim to lower toxicity and improve compatibility with cells (Roma-rodrigues et al., 2020). In addition, stimulus-responsive nanomaterials allow the controlled release of CRISPR at specific places inside cells; this supports the development of safer nanomaterial-based delivery systems and helps address current challenges (Dannhäuser et al., 2022).

CRISPR technology solves many of the challenges of older gene-editing tools such as zinc finger nucleases and TALENs by offering a simpler and more flexible approach (Yadav MR. et al., 2023). The easy design of guide RNAs for chosen DNA sequences improves accuracy, making CRISPR a cost-effective and scalable method. This is why researchers and clinicians prefer it for creating precise and efficient genome modifications (Sharshar et al., 2020). In addition to correcting genetic diseases, precision genome editing can be used to create genetically modified organisms in agriculture, produce disease-resistant crops, and design microbes for better biofuel production (Niazian et al., 2021). It also helps scientists study gene function and regulation, which supports new knowledge about biological processes (Edagwa et al., 2017). Its possible uses in cancer immunotherapy and treatment of infectious diseases show how powerful and wide-ranging this technology is for both science and industry (Liu et al., 2018). However, avoiding off-target effects with CRISPR remains a key concern (Shen et al., 2021). Current research is working to improve CRISPR accuracy through better guide RNAs and modified Cas proteins (Ma et al., 2017). In addition, bioinformatics tools are used to predict possible off-target sites, which helps scientists design improved guide RNA sequences (Shen et al., 2022). Innovative CRISPR variants that show reduced off-target effects are being developed, and this supports the continued progress and reliability of CRISPR technology (Pezoa et al., 2022). A detailed understanding of the factors that influence off-target effects enables researchers to design strategies that can reduce these problems (feng et al., 2023). This commitment helps overcome one of the major limitations of CRISPR and improves its reliability (Fiaz et al., 2021). At the same time, nanomaterial-based delivery systems are advancing, and researchers are working to make them safer and more effective (Ladu, 2021). Surface modifications and functionalizations are especially important in lowering cytotoxicity and improving biocompatibility, thus making these nanocarriers more dependable for precise genome editing (Mushtaq et al., 2019). Another important step is the creation of stimulus-responsive nanomaterials which allow CRISPR components to be released in a controlled way inside specific target cells (Doroftei et al., 2021). Such precision makes genome editing techniques more accurate and reduces the risks linked to off-target effects (Corte et al., 2019). In genome editing approaches, CRISPR remains dominant because it is simple, flexible, and cost-effective (Ferdous et al., 2022). Its programmable nature allows scientists to adapt it to different genetic modifications, making it a suitable choice for many applications (Khan, 2019). Other tools, such as ZFNs and TALENs, are available, but their complex design and reduced flexibility make them less widely used than CRISPR (Katayama et al., 2016). The applications of precision genome editing go far beyond fixing genetic diseases (Popova et al., 2023). This technology also has great value in agriculture, where genetically modified organisms are created to increase crop yields and increase plant resistance to disease (Calos, 2017). It is also used for engineering microbes that can improve biofuel production, thus supporting sustainable energy solutions (Haque et al., 2021). In addition to these industrial uses, precision genome editing helps scientists study gene function and regulation, which leads to progress in many biological processes (van de Wiel et al., 2017). The medical use of genome editing is also clear in cancer immunotherapy and in treating infectious diseases (Feng et al., 2018). Because it can change genes very accurately, it gives researchers new ways to develop targeted therapies that may change how diseases are treated (Zych et al., 2018). As studies continue, the wide use and strong impact of genome editing become more obvious, influencing the future of both medicine and biotechnology (Maity et al., 2023). A strong connection between nanomaterials and genome editing has also opened new opportunities (Agapito-Tenfen, 2016). Nanomaterials, with their special properties, make it possible to deliver gene-editing tools more effectively and with higher precision, solving problems in methods such as CRISPR (Su et al., 2016). As new delivery systems using nanomaterials improve and CRISPR technology itself becomes more advanced, genome editing will likely transform medicine, agriculture, and many other industries (Lamboro et al., 2021).

### 3.2 Nanomaterial-based delivery systems

**FIGURE 3:**
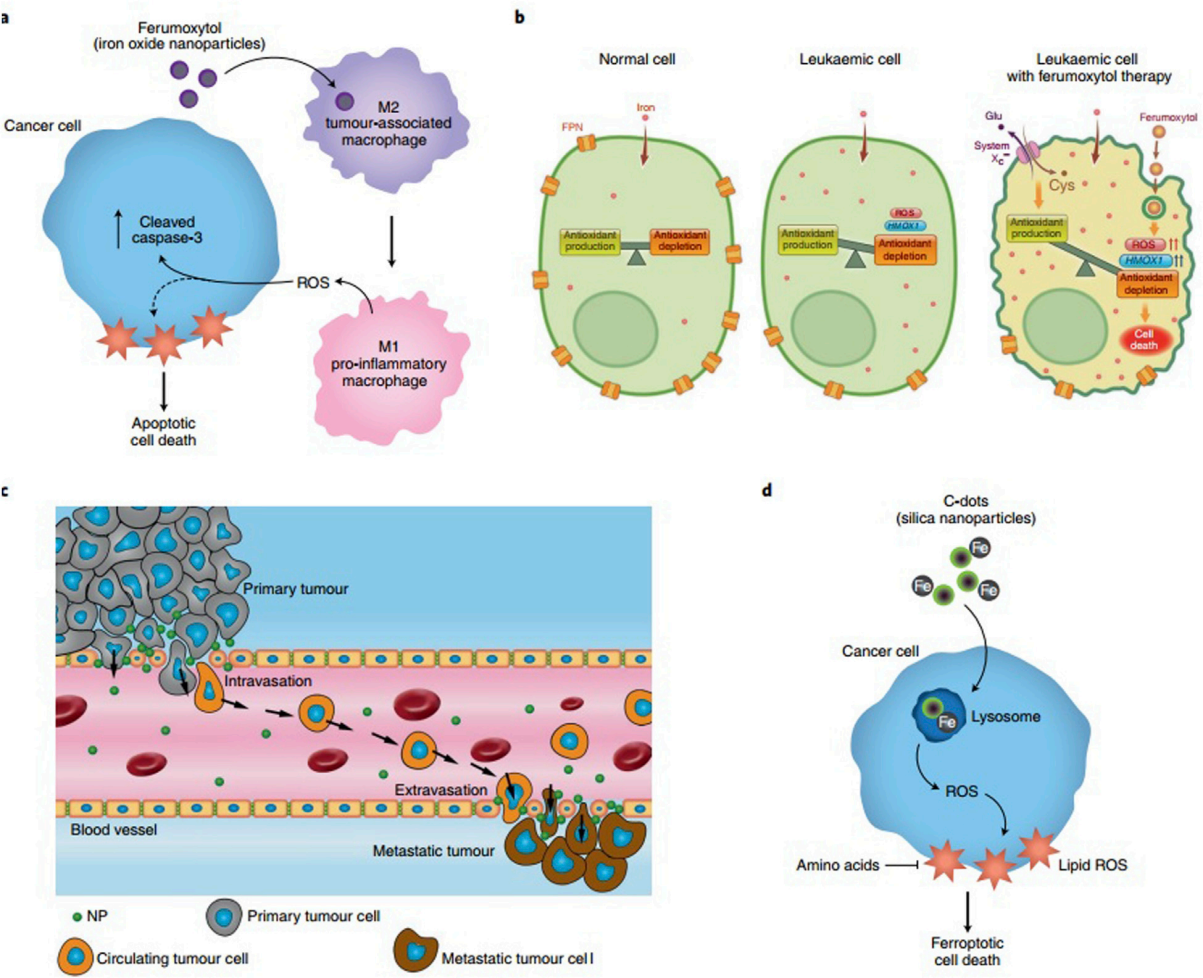
Mechanisms by which nanoparticle-induced ancillary effects impact cancer. **(a)** Ferumoxytol iron oxide nanoparticles modulate macrophage polarization in tumors, promoting M1 polarization and inducing cancer cell death through extracellular reactive oxygen species (ROS). **(b)** Uptake of ferumoxytol by iron-retaining leukemia cells activates the ferroptotic pathway, leading to cell death. **(c)** Titanium dioxide nanoparticles alter the permissiveness of endothelial cells in the microvasculature, thereby facilitating metastasis. **(d)** Carbon dots (C-dots) accumulate iron *in vivo*, triggering ferroptotic cell death in cancer cells. FPN, ferroportin. Reprinted from Stater et al. (2021) with permission from Springer Nature.

**FIGURE 4:**
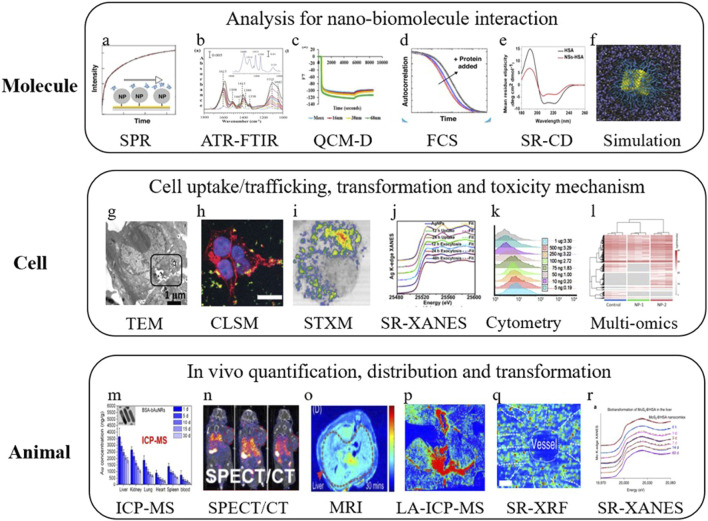
Analytical methods used to study nano–bio interactions at different levels. Diagram shows **(a)** surface plasmon resonance (SPR) as an indirect method for analyzing the biocorona; **(b)** ATR-FTIR spectra that monitor glycine adsorption on TiO_2_ nanoparticles over time; **(c)** QCM-D spectra that present frequency changes during fibrinogen adsorption on surfaces with different topographies; **(d)** fluorescence correlation spectroscopy (FCS) as an indirect method to study nanomaterial–protein interactions; **(e)** synchrotron radiation circular dichroism (SR-CD) spectra of HSA in PBS before and after contact with nanosheets (NSs); **(f)** schematic view of the internalization pathway of cubic PEGylated nanoparticles; **(g)** transmission electron microscopy (TEM) images of cells and Ag nanoparticles; **(h)** effect of protein corona on cell adhesion and survival; **(i)** soft X-ray transmission microscopy (STXM) dual-energy contrast images displaying macrophage uptake of Gd@C_82_(OH)_22_ *in vivo*; **(j)** X-ray absorption near-edge structure (XANES) analysis of silver chemical forms during uptake and clearance in cells; **(k)** flow cytometry used to detect exposed TGF-β1 in the protein corona; **(l)** multi-omics analysis for toxicity evaluation in risk assessment; **(m–o)** quantification of *in vivo* nanomaterial distribution using inductively coupled plasma mass spectrometry (ICP-MS), single-photon emission computed tomography/computed tomography (SPECT/CT), and magnetic resonance imaging (MRI); **(p)** laser ablation–ICP-MS gold imaging showing PEG-GNP movement and intra-organ distribution in the liver; **(q, r)** synchrotron radiation X-ray fluorescence (SR-XRF) microscopy and XANES mapping of molybdenum in the liver together with the characterization of *in vivo* biotransformation of MoS_2_. Reprinted from Liu et al. (2022a) with permission from Elsevier.

Nanomaterial-based delivery systems play an important role in enhancing the targeted delivery of therapeutic agents by using the special properties of nanoparticles, liposomes, and viral vectors. Figure 3 shows the mechanisms through which nanoparticle-related effects influence cancer (Gogolev et al., 2021). Because of their very small size, nanoparticles can pass through biological barriers, reach certain cells or tissues, and facilitate accurate drug delivery (Ansari et al., 2020). The lipid bilayer structure of liposomes is similar to cell membranes, which helps in cellular uptake and provides the controlled release of therapeutic agents (Zhou et al., 2022). Viral vectors, which are safely modified from viruses, act as highly effective carriers that deliver genetic material to target cells with strong accuracy. Figure 4 presents the use of different analytical methods for studying nano–bio interactions at various levels.

Nanoparticles offer several advantages in drug delivery, making them indispensable in the field. Their small size enables efficient cellular uptake, delivering therapeutic agents directly into target cells (Joun et al., 2022). The high surface-area-to-volume ratio allows for functionalization, tailoring surface properties for specific applications (Rong et al., 2020). Additionally, tunable release kinetics ensure controlled and sustained drug release, thus optimizing therapeutic efficacy (Ibrahim et al., 2022). Biocompatible and biodegradable nanoparticle formulations enhance their safety profile, making them ideal carriers for various therapeutic compounds (Turcheniuk and Mochalin, 2017a). Table 2 presents an overview of nanomaterial-based delivery systems, showcasing various advances and applications in the field.

**TABLE 2:** Nanomaterial-based delivery systems.

Nanomaterial type	Description	Advantage	Disadvantage	Application	Example	Current research focus	Future potential	Reference
Polymeric nanoparticles	Biocompatible and biodegradable polymers used to encapsulate drugs	Improved drug solubility, controlled release, and targeted delivery	Potential immunogenicity and limitations in large-scale production	Cancer treatment, gene therapy, and vaccines	PLGA nanoparticles and liposomes	Improving targeting efficiency and reducing side effects	Personalized medicine and combination therapy	Ding et al. (2022)
Mesoporous silica nanoparticles	Highly ordered porous structure for drug loading	High drug loading capacity, sustained release, and biocompatibility	Potential for premature drug release and limited understanding of long-term effects	Drug delivery, imaging, and biocatalysis	MCM-41 and SBA-15	Enhancing controlled release and bioimaging capabilities	Theranostics and combination therapy with imaging and treatment	Ghormade et al. (2011)
Metallic nanoparticles	Nanoparticles made of various metals like gold or silver	Unique optical properties, potential for thermal therapy, and imaging	Potential toxicity and concerns about environmental impact	Cancer treatment, antimicrobial applications, and diagnostics	Gold and silver nanoparticles	Optimizing biocompatibility and exploring theranostic applications	Multifunctional nanoparticles for combined diagnosis and treatment	Koupaei et al. (2019)
Lipid-based nanoparticles	Nanoparticles made from natural or synthetic lipids	Biocompatible, improved drug solubility, and controlled release	Potential for instability and challenges in large-scale production	Drug delivery, gene therapy, and vaccines	Liposomes and micelles	Enhancing targeting and controlled release and overcoming drug resistance	Next-generation vaccines and siRNA delivery	Kozielski et al. (2014)
Carbon nanotubes	Cylindrical structures of carbon atoms with unique properties	High drug loading capacity, potential for targeted delivery, and imaging	Potential toxicity and concerns about environmental impact	Drug delivery, gene therapy, and tissue engineering	Single-walled nanotubes and multi-walled nanotubes	Addressing safety concerns and exploring theranostic applications	Targeted drug delivery for complex diseases	Morán et al. (2018)
Dendrimers	Branched, tree-like synthetic molecules	High drug loading capacity and versatility in surface modification	Potential for immunogenicity and complex and expensive synthesis	Drug delivery, gene therapy, and imaging	PAMAM dendrimers	Improving biocompatibility and targeting efficiency	Multifunctional platforms for theranostics and imaging	Wang et al. (2017)
Quantum dots	Semiconductor nanocrystals with unique optical properties	Efficient light emitters and potential for imaging and drug delivery	Potential toxicity and concerns about environmental impact	Imaging, drug delivery and biosensing	CdSe/ZnS quantum dots	Enhancing biocompatibility and brightness and exploring theranostic applications	Image-guided surgery and personalized medicine	Liang et al. (2023)
Micelles	Colloidal aggregates formed by amphiphilic molecules	Improved drug solubility, controlled release, and potential for targeted delivery	Potential for instability and limitations in drug loading capacity	Drug delivery, gene therapy, and imaging	Polymeric micelles	Optimizing stability and controlled release and exploring theranostic applications	Co-delivery of drugs and imaging agents for cancer treatment	González-Reyna et al. (2023)
Polymeric micelles	Amphiphilic block copolymers self-assembling into micelles	Improved drug solubility, controlled release, and potential for targeted delivery	Potential for instability and limitations in drug loading capacity	Drug delivery, gene therapy, and imaging	Polymeric micelles	Optimizing stability and controlled release and exploring theranostic applications	Co-delivery of drugs and imaging agents for cancer treatment	Wang et al. (2019)
Gold nanorods	Anisotropic gold nanoparticles with unique optical properties	Photothermal therapy, imaging, and drug delivery	Potential complexity in synthesis and concerns about long-term effects	Cancer treatment, photothermal ablation, and bioimaging	Gold nanorods	Enhancing targeting and therapeutic efficacy and exploring theranostic applications	Multimodal therapy combining photothermal therapy with other modalities	Imlimthan et al. (2020)
Superparamagnetic iron oxide nanoparticles (SPIONs)	Magnetic nanoparticles for biomedical applications	Magnetic resonance imaging (MRI) contrast agents, drug delivery, and hyperthermia therapy	Potential for aggregation and concerns about long-term effects	Diagnostics, drug delivery, theranostics	SPIONs	Improving biocompatibility and targeting efficiency and exploring theranostic applications	MRI-guided drug delivery and theranostics	Zhao et al. (2020)
Polymeric micelles	Amphiphilic block copolymers self-assembling into micelles	Improved drug solubility, controlled release, and potential for targeted delivery	Potential for instability and limitations in drug loading capacity	Drug delivery, gene therapy, and imaging	Polymeric micelles	Optimizing stability and controlled release and exploring theranostic applications	Co-delivery of drugs and imaging agents for cancer treatment	Sami et al. (2012)
Exosomes	Endogenous nanovesicles derived from cells	Biocompatible, natural carriers for drug delivery, and potential for targeted therapy	Limited drug loading capacity and challenges in large-scale production	Drug delivery, gene therapy, and vaccines	Exosomes	Enhancing drug loading capacity and targeting efficiency and overcoming biological barriers	Next-generation drug delivery systems with improved biocompatibility and targeting	Qamar et al. (2021)
Carbon dots	Carbon-based nanoparticles with unique optical properties	Biocompatible, fluorescence for imaging and potential for drug delivery	Limited understanding of long-term effects and challenges in controlled synthesis	Bioimaging, drug delivery, and biosensing	Carbon dots	Enhancing biocompatibility and brightness and exploring theranostic applications	Multifunctional platforms for imaging, drug delivery, and theranostics	Ihnatsyeu-Kachan et al. (2017)
Polymeric hydrogels	Three-dimensional networks of cross-linked polymers	Biocompatible, sustained drug release, and potential for tissue engineering	Potential for limited drug loading capacity and challenges in controlled degradation	Drug delivery, tissue engineering, and cell therapy	Chitosan hydrogels and hyaluronic acid hydrogels	Improving drug loading and release profiles and exploring theranostic applications	Personalized drug delivery systems with controlled release and biocompatibility	Liu et al. (2022a)

### 3.3 Chitosan

Polymeric nanoparticles show high biocompatibility and adjustable properties, giving them a long residence time in ocular tissues (Lin et al., 2022). They can carry both hydrophilic and hydrophobic drugs, which makes them useful for different therapeutic agents (Liu J. et al., 2021). Their ability to provide sustained release allows for longer therapeutic effects (Banerjee et al., 2019). In addition, they can use both passive and active targeting, improving their value in treating conditions such as glaucoma, age-related macular degeneration, and diabetic retinopathy (Dubey et al., 2023). However, despite these benefits, polymeric nanoparticles also face some limitations (Velluto et al., 2021). They may aggregate, and their loading capacity for hydrophobic drugs is limited (Jain, 2012). These problems can reduce drug delivery efficiency and must be carefully considered during formulation (Miao et al., 2014). Even so, current research is trying to solve these problems and expand the use of polymeric nanoparticles in ocular drug delivery (Li et al., 2022b).

Liposomes, although unstable and costly to produce, are biocompatible and allow good control over drug release (Štimac et al., 2019). They are better suited for hydrophobic drugs and can provide sustained release, making them useful for conditions such as dry eye syndrome and fungal keratitis (Soroush et al., 2016). Passive targeting is possible, but their stability problems encourage more studies to improve their design and manufacturing (Singh et al., 2013). Chitosan-based implants provide sustained release, good biocompatibility, and biodegradability, which make them effective for long-term treatment (Yu et al., 2016a). The main disadvantage is that they require surgical implantation (Dash et al., 2021). Nevertheless, because of their extended drug release and compatibility, they are promising for treating ocular diseases such as glaucoma and retinoblastoma (Arjama et al., 2018). Chitosan-coated prodrugs improve drug solubility and stability, and they can deliver drugs directly to ocular tissues (Yu et al., 2016a). Their complex design and synthesis are challenges, but their controlled release and potential for active targeting make them valuable for ocular diseases (Aseri et al., 2015). These prodrugs show strong potential in drug delivery, especially for conditions that need better solubility, stability, and targeted release (Santana et al., 2022).

### 3.4 Alginate

In oral drug delivery, alginate has an important role as it is used in tablets and capsules for delivering proteins, peptides, and nucleotides (Ashique et al., 2023). The release system is mainly pH-sensitive, which makes it possible to deliver drugs to selected parts of the gastrointestinal tract (Hastings et al., 2015). Alginate is often chosen because it is biocompatible and non-toxic, making it a good material for controlled and sustained drug release (Madl et al., 2012). However, alginate can also face the problem of gel erosion, which may require extra solutions such as enteric coatings (Pan et al., 2021). These coatings stop the drug from being released too early and help ensure proper delivery (Gupta, 2020). In practice, alginate is applied in the form of tablets and capsules for proteins, peptides, and nucleotides (Khan et al., 2023). The release system works in a pH-sensitive way, allowing targeted release in certain regions of the gastrointestinal tract (Yadav P. et al., 2023). Because it is safe and non-toxic, alginate is a suitable material for achieving long-term and steady drug release (Lan et al., 2020). Despite these benefits, the problem of gel erosion when using alginate may require the use of enteric coatings (Tang et al., 2010). These help prevent early drug release and support proper drug delivery (Yu et al., 2016b). In oral drug delivery, alginate is used in making tablets and capsules for delivering proteins, peptides, and nucleotides (Kotb et al., 2023). The main release method is pH-sensitive, which ensures drug delivery to exact areas of the gastrointestinal tract (Dai et al., 2021). Alginate is chosen because it is biocompatible and non-toxic, which makes it suitable for controlled drug release (Khan et al., 2023). However, the risk of gel erosion linked with alginate may make enteric coatings necessary (Manikantan et al., 2023). These coatings work as a protective layer, stopping early drug release and helping the drug delivery system to remain effective (Chen et al., 2017). In oral drug delivery, alginate is applied in preparing tablets and capsules that carry proteins, peptides, and nucleotides (Xavier Mendes et al., 2021). The use of a pH-sensitive system guarantees targeted drug delivery to the right parts of the gastrointestinal tract (Shafee et al., 2021). Because alginate is safe and non-toxic, it is a strong option for continuous drug release (Cai and Xu, 2011). Nevertheless, the issue of gel erosion in alginate use raises the need for extra methods, such as adding enteric coatings (Pillai et al., 2018). These coatings act as protection, avoiding early release of drugs and keeping the drug delivery process effective (Turcheniuk and Mochalin, 2017b). In oral drug delivery, alginate plays an important role in making tablets and capsules for proteins, peptides, and nucleotides (Kausar, 2021). The main idea of its release mechanism is based on pH sensitivity, which allows drugs to be released carefully and only in certain parts of the gastrointestinal tract (Lin and Mao, 2011). Alginate has many useful properties, such as being biocompatible and non-toxic, which make it suitable for controlled drug release (Barua et al., 2020). However, one problem with alginate is gel erosion, and this requires a practical solution, such as using enteric coatings (Kang et al., 2017). These coatings act as a barrier to protect the drug, lowering the chance of early release and keeping the accuracy and effectiveness of the delivery process (Barhoum et al., 2022). In oral drug delivery, alginate is an important material used in tablets and capsules designed to release proteins, peptides, and nucleotides (Lupu et al., 2020). The key principle of its release system depends on pH sensitivity, which helps the drug reach specific areas of the gastrointestinal tract (Szczeszak et al., 2015). Because alginate is biocompatible and non-toxic, it is a good choice for supporting controlled and sustained drug release (Vélez et al., 2017). Still, the risk of gel erosion when using alginate makes it necessary to find other solutions, such as enteric coatings (Ohyanagi et al., 2011). These coatings work as a protective shield, reducing the chance of drugs releasing too early and making sure drug delivery remains precise and effective (Hossain CM. et al., 2021).

### 3.5 Liposomes

Liposomes and alginate are two distinct systems in the field of drug delivery, each defined by specific characteristics and applications (Kapoor et al., 2018). Structurally, liposomes are spherical vesicles that consist of a phospholipid bilayer membrane, while alginate is a linear polysaccharide composed of β-D-mannuronic acid and α-L-guluronic acid residues (Giordani, 2020). The architecture of liposomes is more complex than alginate (McDonald et al., 2015). This structural complexity, however, allows liposomes to provide greater flexibility in modifying their composition for targeted drug delivery (Opanasopit et al., 2008). In contrast, alginate is a natural and biodegradable polymer, which highlights its environmentally friendly properties (Amreddy et al., 2017). With respect to charge, liposomes may be neutral, cationic, or anionic, making it possible to adjust their surface charges for specific cellular interactions (Zhou et al., 2018). For example, cationic liposomes can improve cellular uptake through electrostatic attraction (Khaliq et al., 2023). Alginate, although typically non-ionic, can be engineered into ionic forms, demonstrating its adaptability in charge modification (Han et al., 2021). Among the major benefits of liposomes is their capacity to be functionalized with ligands, enabling targeted delivery to particular cells and offering additional strategies for selective drug administration through ligand conjugation (Mashima and Takada, 2022). Alginate, unlike liposomes, can reach particular tissues through passive targeting mechanisms (Majidi et al., 2016). Liposomes may show instability under physiological conditions, which creates the need for structural modifications to enhance their stability (Wang et al., 2015). In contrast, alginate is normally stable in physiological environments, making it a stronger option in this aspect (Luly et al., 2020). Both liposomes and alginate are biocompatible. Liposomes are also mucoadhesive, which makes them appropriate for wound healing and for drug delivery in the gastrointestinal tract (Khaliq et al., 2023). The drug loading ability of these carriers shows different features (Xu et al., 2022). Liposomes can encapsulate both hydrophilic and hydrophobic drugs, and they provide higher efficiency for a wide range of molecules (Tian et al., 2013). Furthermore, liposomes can be engineered for sustained or controlled release (Pissuwan et al., 2011). Alginate, mostly used for hydrophilic drugs, can form hydrogels that allow localized drug delivery (Zhu et al., 2022). Applications of liposomes include drug delivery, hydrophobic drug encapsulation, gene therapy, and cosmetic products (Khaliq et al., 2023). Alginate, on the other hand, is widely applied in cell encapsulation, tissue engineering, and controlled release systems (Shen L. et al., 2018). Toxicity must also be considered in comparison. Liposomes are mostly well-tolerated but may cause dose-dependent toxicity (Shafee et al., 2021). Alginate is generally non-toxic, although it may trigger some immune response (Erel-Akbaba et al., 2021). It is often described as less toxic than certain liposome formulations, strengthening its safety profile (Lai et al., 2017). Both materials are important for drug delivery: Liposomes offer versatility and targeting potential, while alginate provides cost-effectiveness and mechanical strength for applications such as tissue engineering, cell encapsulation, and pharmaceutical systems (Lin et al., 2015). Cost is another critical factor (Casper et al., 2023). Liposomes are usually more costly to produce, while alginate is cheaper and therefore more suitable for large-scale pharmaceutical production (Davis, 2009). This cost advantage makes alginate an appealing alternative in broader drug delivery applications (Luly et al., 2020). The targeting abilities of liposomes extend beyond charge modification and include functionalization with ligands for specific cellular interactions (Deng et al., 2019). This property enables liposomes to provide more strategies for targeted drug delivery through ligand conjugation (Khanna et al., 2023). In comparison, alginate, although less versatile in this aspect, compensates with its ability to achieve tissue-specific delivery via passive targeting mechanisms (Lima et al., 2019). These differences demonstrate the unique strengths of each material in overcoming challenges in drug delivery (Naik et al., 2022). The application context further highlights the complementary roles of liposomes and alginate (Wang et al., 2021). Liposomes are particularly effective in drug delivery, especially for encapsulating hydrophobic drugs, in gene therapy, and in cosmetic formulations (Xu et al., 2021). Alginate, however, is mainly applied in cell encapsulation, tissue engineering, and controlled drug release (Shen Y. et al., 2018). The wide range of applications illustrates the distinct features each material contributes, confirming their importance in the biomedical field (Ghormade et al., 2011).

With regard to toxicity, liposomes are generally well tolerated, although dose-dependent toxicities can occur (Koupaei et al., 2019). By contrast, alginate is widely recognized as non-toxic, although it may trigger immune responses in certain cases (Kozielski et al., 2014). Its favorable safety profile, being less toxic than some liposome formulations, emphasizes its suitability for biomedical use where minimizing adverse effects is critical (Morán et al., 2018). Overall, liposomes and alginate present specific advantages and characteristics in the field of drug delivery (Wang et al., 2017). Liposomes are notable for their versatility, ability to manipulate charge, and potential for ligand-mediated targeting (Liang et al., 2023). Alginate is distinguished by its natural, biodegradable qualities, cost-effectiveness, physiological stability, and its applications in tissue engineering and cell encapsulation (González-Reyna et al., 2023). The combination of these advantages positions both liposomes and alginate as valuable elements in advancing drug delivery systems, with their individual attributes addressing different therapeutic and biomedical requirements (Wang et al., 2019).

## 4 Applications in CRISPR-based genome editing

Applications of CRISPR-based genome editing involve a wide variety of nanomaterials, each used through specific delivery methods to improve the precision and efficiency of gene modification (Imlimthan et al., 2020). Lipid nanoparticles, applied via encapsulation, have shown enhanced delivery performance and reduced off-target activity in human T cells (Zhao et al., 2020). In particular, editing the *PD-1* gene, an immune checkpoint, produced stronger T cell responses against cancer cells, thus demonstrating significant potential for progress in cancer immunotherapy (Sami et al., 2012). Mesoporous silica nanoparticles, when conjugated with Cas9, provided greater stability and protection of the Cas9 enzyme (Qamar et al., 2021). This method, when applied to rice plants, successfully targeted the OsSWEET11 gene (a sugar transporter), producing increased drought resistance (Ihnatsyeu-Kachan et al., 2017). Carbon nanotubes, functionalized with guide RNA, displayed improved cellular uptake and efficient targeted delivery in mice (Liu et al., 2022a). By editing the *HPRT1* (hypoxanthine–guanine phosphoribosyltransferase 1) gene, this approach corrected a genetic defect, serving as a model for therapeutic applications (Chen et al., 2017). Polymer nanoparticles carrying CRISPR components allowed controlled release and sustained delivery in bacteria (Xavier Mendes et al., 2021). Targeting multidrug resistance genes led to greater sensitivity to antibiotics, offering a potential strategy for overcoming antibiotic resistance (Shafee et al., 2021). Exosomes engineered for CRISPR delivery functioned as natural carriers with enhanced biocompatibility and selective targeting (Cai and Xu, 2011). In human stem cells, this approach was applied to the *β-globin* gene (hemoglobin subunit), indicating a possible treatment for sickle cell disease (Pillai et al., 2018). Magnetic nanoparticles manipulated by an external magnetic field have enabled spatially directed delivery to specific regions of zebrafish embryos. Editing the *EGFP* gene (fluorescent protein) permitted precise modifications in defined tissues, highlighting opportunities for spatially controlled genome editing (Priyadarsini et al., 2018). Gold nanoparticles, integrated with photothermal ablation and CRISPR, demonstrated a synergistic strategy against cancer cells. In human cancer cells, targeting the *EGFR* (epidermal growth factor receptor) gene enabled both precise editing and cancer cell destruction—an innovative method reported by Mirón-Barroso et al. (2021). Table 3 provides a summary of successful genome editing applications using nanomaterials.

**TABLE 3:** Examples of successful genome editing applications using nanomaterials.

Target organism	Nanomaterial	Genome editing tool	Application	Outcome	Delivery method	Advantage	Challenge	Reference
Rice	Chitosan nanoparticles	CRISPR/Cas9	Increased drought tolerance	Improved crop yield under drought conditions	Particle bombardment	Enhanced delivery efficiency and reduced off-target effects	Requires optimization for large-scale application	Turcheniuk and Mochalin (2017b)
Human cells	Polymeric micelles	CRISPR/Cas9	Correction of a genetic mutation causing beta-thalassemia	Reduced disease severity in preclinical models	Lipid nanoparticle-mediated delivery	Targeted delivery and improved biocompatibility	Potential for off-target effects and requires further safety testing	Kausar (2021)
Bacteria	Mesoporous silica nanoparticles	CRISPR/Cas9	Elimination of antibiotic-resistant genes	Reduced spread of antibiotic-resistant bacteria	Electroporation	Efficient delivery and potential for clinical applications	Requires further development for *in* *vivo* use	Lin and Mao (2011)
Mushrooms	Lipid nanoparticles	CRISPR/Cas9	Increased production of valuable metabolites	Enhanced production of commercially valuable compounds	Protoplast transformation	Targeted delivery and high editing efficiency	Requires optimization for specific fungi strains	Barua et al. (2020)
Animals (mice)	Gold nanoparticles	TALENs	Correction of a genetic mutation causing muscular dystrophy	Improved muscle function in preclinical models	Intramuscular injection	Efficient delivery to muscle tissue	Requires further development for systemic delivery	Kang et al. (2017)
Plants (tomatoes)	Carbon nanotubes	CRISPR/Cas9	Enhanced resistance to a viral disease	Reduced crop losses due to viral infection	Agrobacterium-mediated transformation	Improved delivery efficiency and precise gene editing	Potential cytotoxicity of carbon nanotubes and requires further safety assessment	Barhoum et al. (2022)
Human cells (cancer)	Polymeric nanoparticles	CRISPR/Cas9	Knockdown of a cancer-promoting gene	Reduced tumor growth in preclinical models	Electroporation	Targeted delivery to cancer cells	Requires further development for clinical applications and potential for off-target effects	Lupu et al. (2020)
Yeast	Liposomes	CRISPR/Cas9	Improved production of biofuels	Increased efficiency of biofuel production	Electroporation	Efficient delivery and improved biocompatibility	Requires optimization for large-scale fermentation processes	Szczeszak et al. (2015)
Fruit flies (*Drosophila*)	Carbon dots	CRISPR/Cas9	Modeling human genetic diseases	Improved understanding of disease mechanisms	Microinjection	Efficient delivery to specific cell types	Requires further development for high-throughput applications	Vélez et al. (2017)
Algae	Silica nanoparticles	CRISPR/Cas9	Increased lipid production for biofuels	Enhanced production of renewable energy sources	Electroporation	Targeted delivery and improved biocompatibility	Requires optimization for specific algal strains	Ohyanagi et al. (2011)
Human cells (stem cells)	Exosomes	CRISPR/Cas9	Correction of a genetic mutation causing sickle cell disease	Improved clinical outcomes in preclinical models	Viral transduction	Targeted delivery to stem cells and potential for regenerative medicine	Requires further development for clinical applications and potential for off-target effects	Hossain et al. (2021b)
Plants (corn)	Graphene oxide nanoparticles	CRISPR/Cas9	Improved nutritional value	Enhanced food security and nutrition	Particle bombardment	Efficient delivery and improved editing efficiency	Requires further safety assessment of graphene oxide nanoparticles	Kapoor et al. (2018)
Bacteria (*E. coli*)	Magnetic nanoparticles	CRISPR/Cas9	Creation of living biosensors for environmental monitoring	Improved detection of pollutants and toxins	Electroporation	Efficient delivery and potential for real-world applications	Requires further development for long-term stability and sensitivity	Giordani (2020)
Animals (pigs)	Polymeric nanoparticles	Base editing	Correction of a genetic mutation causing cystic fibrosis	Improved lung function in preclinical models	Intravenous injection	Targeted delivery and precise gene editing	Requires further development for clinical applications and potential for off-target effects	McDonald et al. (2015)
Plants (potatoes)	Chitosan-coated gold nanoparticles	CRISPR/Cas9	Increased resistance to a fungal disease	Reduced crop losses due to fungal infection	Agroinfiltration	Targeted delivery and improved disease resistance	Requires optimization for specific plant diseases and environmental conditions	Opanasopit et al. (2008)
Human cells (immune cells)	Dendrimers	CRISPR/Cas9	Engineering T cells for cancer immunotherapy	Improved efficacy of cancer treatment in preclinical models	Electroporation	Targeted delivery to immune cells and potential for personalized medicine	Requires further development for clinical applications and potential for off-target effects	Amreddy et al. (2017)
Animals (zebrafish)	Mesoporous silica nanoparticles	CRISPR/Cas9	Modeling human developmental disorders	Improved understanding of disease development	Microinjection	Efficient delivery to embryos and high editing efficiency	Requires further development for large-scale studies	Zhou et al. (2018)
Plants (soybeans)	Polymeric micelles	Base editing	Increased oil production	Enhanced food production and biofuel potential	Particle bombardment	Precise gene editing and reduced off-target effects	Requires optimization for specific agricultural traits and environmental conditions	Khaliq et al. (2023)
Bacteria (*Lactococcus lactis*)	Liposomes	CRISPR/Cas9	Engineering bacteria for food production	Improved production of food additives and probiotics	Electroporation	Targeted delivery and potential for industrial applications	Requires further development for large-scale fermentation processes and safety assessment	Han et al. (2021)
Animals (rats)	Lipid nanoparticles	CRISPR/Cas9	Correction of a genetic mutation causing Huntington’s disease	Improved neurological function in preclinical models	Intranasal delivery	Targeted delivery to the brain and potential for neurological disease treatment	Requires further development for clinical applications and safety assessment	Mashima and Takada (2022)
Human cells (neurons)	Exosomes derived from stem cells	CRISPR/Cas9	Treatment of neurodegenerative diseases	Improved neuronal function in preclinical models	Viral transduction	Targeted delivery to neurons and potential for personalized medicine	Requires further development for clinical applications and potential for off-target effects and ethical considerations	Majidi et al. (2016)
Plants (cotton)	Carbon dots	CRISPR/Cas9	Improved fiber quality	Enhanced textile production and economic benefits	Agrobacterium-mediated transformation	Efficient delivery and reduced environmental impact	Requires further safety assessment of carbon dots and optimization for specific cotton varieties	Wang et al. (2015)
Bacteria (*Salmonella*)	Magnetic nanoparticles	CRISPR-Cpf1	Development of live vaccines	Improved immunity against bacterial infections	Electroporation	Efficient delivery and potential for novel vaccine development	Requires further development for clinical applications and safety assessment of CRISPR-Cpf1	Luly et al. (2020)
Animals (dogs)	Polymeric nanoparticles	Base editing	Correction of a genetic mutation causing muscular dystrophy	Improved muscle function in preclinical models	Intravenous injection	Targeted delivery and precise gene editing	Requires further development for clinical applications and safety assessment of base editing technology	Xu et al. (2022)
Animals (mice)	Dendrimers	TALENs	Correction of a genetic mutation causing blindness	Improved vision in preclinical models	Intravenous injection	Targeted delivery to specific organs and potential for vision restoration therapies	Requires further development for clinical applications and safety assessment of TALENs	Tian et al. (2013)
Human cells (fibroblasts)	Liposomes	CRISPR/Cas9	Editing genes associated with aging	Extended lifespan in preclinical models	Electroporation	Targeted delivery to specific cell types and potential for anti-aging therapies	Requires further development for clinical applications, ethical considerations, and safety assessment of potential unintended consequences	Pissuwan et al. (2011)
Plants (rice)	Carbon nanotubes	Base editing	Increased salt tolerance	Improved crop yield in saline environments	Agrobacterium-mediated transformation	Precise gene editing and enhanced stress tolerance	Requires further safety assessment of carbon nanotubes and optimization for specific rice varieties	Zhu et al. (2022)
Bacteria (*Streptococcus pneumoniae*)	Magnetic nanoparticles	CRISPR/Cas9	Development of next-generation vaccines	Improved immunity against bacterial infections	Electroporation	Efficient delivery and potential for multi-strain vaccines	Requires further development for clinical applications and safety assessment of CRISPR/Cas9 in humans	Shen et al. (2018b)
Animals (pigs)	Exosomes	Base editing	Modification of organ compatibility for xenotransplantation	Improved survival rates in preclinical models	Intravenous injection	Targeted delivery to specific organs and potential for addressing organ shortage	Requires further development for clinical applications, ethical considerations, and safety assessment of base editing and xenotransplantation	Erel-Akbaba et al. (2021)

## 5 RNA interference

**FIGURE 5:**
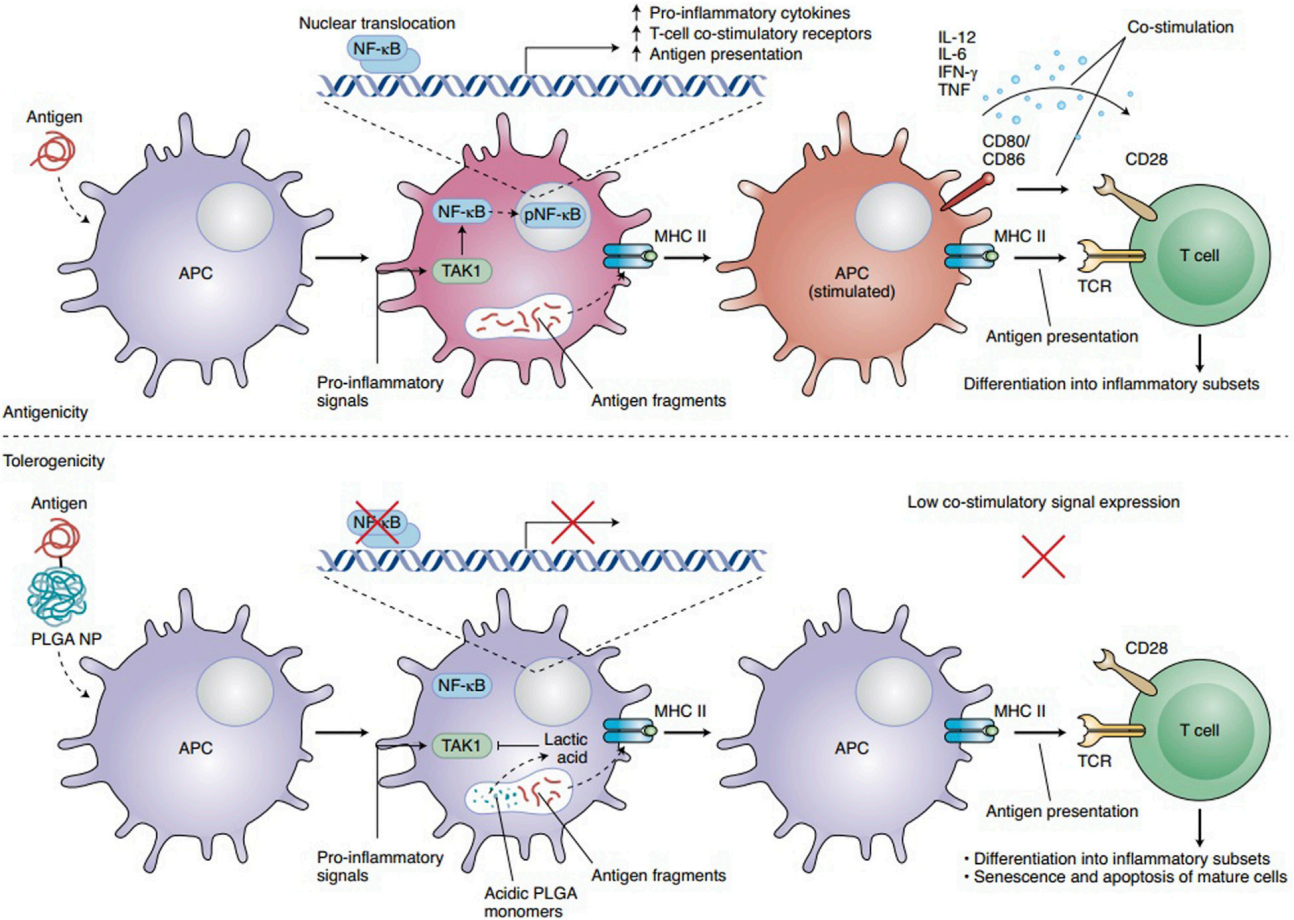
Immunomodulatory effects induced by nanoparticles (NPs) support the establishment of antigen tolerance. In the upper section, the foreign antigen is internalized by antigen-presenting cells (APCs) together with an external pro-inflammatory activation signal. This co-stimulation contributes to the priming of naive T cells, resulting in the activation and/or proliferation of mature antigen-reactive T cells. This occurs through enhanced presentation of antigen fragments via major histocompatibility complexes (MHCs), the expression of juxtacrine co-stimulatory receptors, and the release of pro-inflammatory cytokines. In contrast, the lower section illustrates the co-uptake of antigen with poly (lactic-co-glycolic acid) (PLGA) nanomaterial. The degradation of PLGA into acidic monomers leads to the inhibition of TAK1, mediated by lactic acid, which reduces NF-κB phosphorylation and prevents nuclear translocation of pNF-κB. Consequently, the responsiveness to pro-inflammatory signals acting through TAK1 is weakened. When sufficient co-stimulation is absent, the presentation of antigen fragments to T cells does not activate naive cells and instead promotes the senescence or apoptosis of mature antigen-reactive T cells. The figure also includes essential labels such as IFN-γ (interferon-γ), IL (interleukin), MHC (major histocompatibility complex), TCR (T-cell receptor), and TNF (tumor necrosis factor). Reprinted from Stater et al. (2021) with permission of Elsevier.

Nanomaterials act as important facilitators in RNA interference (RNAi) by creating a stable and protective environment for RNA molecules, such as small interfering RNA (siRNA) (Ferdous et al., 2022). Their unique features, including small size and specific surface characteristics, allow efficient encapsulation and delivery of siRNA to target cells (Su et al., 2016). This encapsulation protects the fragile RNA molecules from enzymatic degradation, increases their stability during transport, and improves their bioavailability once they reach the target cells (Katayama et al., 2016). The precision of nanomaterial-mediated RNAi is based on the ability to specifically regulate disease-associated genes, increasing the effectiveness of therapeutic interventions (Popova et al., 2023). Nanomaterials also influence the pharmacokinetics of RNA interference by improving the circulation and bioavailability of RNA molecules (Calos, 2017). Their small size supports longer circulation in the bloodstream and prevents rapid clearance by the immune system (Zhou et al., 2022). Furthermore, nanomaterials can be designed for enhanced permeability and retention (EPR), which enables selective accumulation at disease sites (van de Wiel et al., 2017). This accumulation raises the concentration of RNA molecules at the target, thus increasing therapeutic efficiency (Feng et al., 2018). By modulating the pharmacokinetics of RNA interference, nanomaterials improve precision and the overall success of therapies (Maity et al., 2023). Nanomaterials also offer a multifaceted approach to targeted delivery in RNA interference (Zych et al., 2018). Through surface modifications and functionalizations, they can be engineered to recognize specific markers on target cells, leading to selective binding and internalization (Agapito-Tenfen, 2016). This targeted delivery ensures the controlled release of RNA molecules at the intended sites and reduces off-target effects (Su et al., 2016). Such precision enhances therapeutic outcomes by concentrating the therapeutic load at the disease site, lowering systemic exposure and decreasing possible side effects (Lamboro et al., 2021). In addition, nanomaterials help overcome barriers linked to RNA interference (Gogolev et al., 2021). RNA delivery often faces problems such as enzymatic degradation, recognition by the immune system, and limited cellular uptake (Ansari et al., 2020). Nanomaterials address these challenges by offering protective encapsulation, shielding RNA from degradation, avoiding immune recognition, and improving cellular uptake (Zhou et al., 2022). These improvements strengthen the precision and effectiveness of therapeutic strategies by ensuring the successful delivery of RNA molecules to target cells while overcoming biological barriers (Singh et al., 2013). Different nanomaterials have been developed to support RNA interference, each enhancing the accuracy and efficiency of therapeutic methods (Agapito-Tenfen, 2016). Lipid-based, polymeric, and inorganic nanoparticles are notable examples (Padayachee and Singh, 2020). Lipid-based nanoparticles provide a lipid bilayer resembling cell membranes, which increases cellular uptake (Falzarano et al., 2015). Polymeric nanoparticles allow the modification of surface properties, making targeted delivery possible (Turcheniuk and Mochalin, 2017a). Inorganic nanoparticles, including gold nanoparticles, show distinctive physicochemical properties that support effective RNA delivery (Ding et al., 2022). The choice of nanomaterial depends on the specific therapeutic aim and the type of RNA molecules delivered. These nanomaterials illustrate the range of strategies used to increase precision in RNA interference and optimize therapeutic performance (Ghormade et al., 2011). Table 4 provides a detailed overview of nanomaterials and their role in RNAi. The wide variety of nanomaterials tested for RNA interference highlights the need to balance benefits and potential limitations for each type (Koupaei et al., 2019). Knowledge of the unique features and restrictions of each nanomaterial is essential for their best use in RNA interference applications (Kozielski et al., 2014). Lipid nanoparticles, which use a complexation loading method, are applied to different cell types (Rong et al., 2020). They show high delivery efficiency with controlled release and stable gene silencing (Mohamad et al., 2023). Their biodegradable structure and flexibility are advantages, but possible off-target effects and cost issues remain concerns (Wang et al., 2017). Carbon nanotubes, loaded through functionalization, are able to target cells that are otherwise hard to reach (Wang et al., 2017). Although their delivery efficiency varies and is still under study, they have strong potential for selective delivery (Liang et al., 2023). However, safety risks and aggregation remain major drawbacks (Song et al., 2023). Metal nanoparticles, applied through conjugation, can target a variety of cell types (González-Reyna et al., 2023). Their delivery efficiency is moderate and requires improvement, with gene silencing effects also at a moderate level (Wang et al., 2019). Their strengths are biocompatibility and adjustable properties, but disadvantages include toxicity and non-specific interactions (Imlimthan et al., 2020). Dendrimers, commonly loaded through encapsulation, are utilized for delivery across different cell types, showing delivery efficiency and gene silencing efficiency that depend on their size (Zhao et al., 2020). These nanoparticles are biocompatible and adaptable; however, potential toxicity and the difficulty of achieving controlled release remain important concerns (Sami et al., 2012). Exosomes, functioning as natural carriers, demonstrate strong delivery efficiency with inherent targeting across multiple cell types (Morán et al., 2018). Their advantages include biocompatibility and low immunogenicity, although their restricted loading capacity and variability between batches present challenges (Zhang L. et al., 2022). Inorganic nanoparticles, such as gold nanoparticles, are generally loaded through conjugation and are directed toward particular cell types (Khaliq et al., 2023). Their delivery efficiency continues to vary and remains the subject of ongoing research, as does their variable gene silencing efficiency (Dodds, 2023). These nanoparticles offer unique properties, including imaging functions, and display potential for controlled release (Lin et al., 2022). Nevertheless, safety issues and the incomplete understanding of their mechanisms represent significant disadvantages (Liu J. et al., 2021). Figure 5 illustrates the immunomodulatory effects achieved through nanoparticle mediation and how these contribute to antigen tolerance.

**TABLE 4:** Examination of nanomaterials in facilitating RNA interference.

Nanomaterial type	Delivery mechanism	Advantage	Disadvantage	RNAi target	*In* *vitro* studies	*In* *vivo* studies	Potential application	Reference
Polymeric nanoparticles	Encapsulation and conjugation	High stability, targeted delivery, and controlled release	Potential toxicity and immune system activation	Specific genes and mRNAs	Knockdown efficiency and cellular uptake	Tumor suppression and gene silencing	Cancer therapy, genetic diseases	Dubey et al. (2023)
Lipid nanoparticles	Encapsulation and complexation	High biocompatibility and efficient cellular uptake	Potential off-target effects and instability	Specific genes and mRNAs	Gene knockdown and silencing efficiency	Gene therapy and vaccine development	Infectious diseases and genetic disorders	Velluto et al. (2021)
Carbon nanotubes	Conjugation and functionalization	High loading capacity and specific targeting	Potential cytotoxicity and agglomeration	Specific genes and mRNAs	Gene silencing and cellular uptake	Targeted drug delivery and gene therapy	Cancer treatment and neurodegenerative diseases	Jain (2012)
Gold nanoparticles	Conjugation and functionalization	High biocompatibility and ease of modification	Potential immunogenicity and limited targeting options	Specific genes and mRNAs	Gene knockdown and silencing efficiency	Drug delivery and gene therapy	Cancer treatment and inflammatory diseases	Miao et al. (2014)
Inorganic nanoparticles	Encapsulation and conjugation	High stability and diverse functionalities	Potential toxicity and limited biodegradability	Specific genes and mRNAs	Gene silencing and cellular uptake	Imaging and targeted drug delivery	Cancer diagnosis and theranostics	Li et al. (2022b)
Dendrimers	Encapsulation and conjugation	High functionality and controlled release	Potential cytotoxicity and limited *in vivo* stability	Specific genes and mRNAs	Gene knockdown and silencing efficiency	Drug delivery and gene therapy	Cancer treatment and genetic diseases	Štimac et al. (2019)
Micelles	Encapsulation and conjugation	High biocompatibility and controlled release	Potential stability issues and limited targeting options	Specific genes and mRNAs	Gene knockdown and silencing efficiency	Drug delivery and gene therapy	Cancer treatment and cardiovascular diseases	Soroush et al. (2016)
Liposomes	Encapsulation and conjugation	High biocompatibility and well-established technology	Potential stability issues and limited targeting options	Specific genes and mRNAs	Gene knockdown and silencing efficiency	Drug delivery and gene therapy	Cancer treatment and infectious diseases	Singh et al. (2013)
Exosomes	Encapsulation	Natural origin, efficient cellular uptake, and targeted delivery	Limited loading capacity and complex isolation	Specific genes and mRNAs	Gene knockdown and silencing efficiency	Drug delivery and gene therapy	Cancer treatment and neurodegenerative diseases	Yu et al. (2016a)
Metal–organic frameworks (MOFs)	Encapsulation and conjugation	High biocompatibility, tunable porosity, and surface chemistry	Limited understanding of *in* *vivo* behavior and potential off-target effects	Specific genes and mRNAs	Gene silencing and cellular uptake	Drug delivery and gene therapy	Cancer treatment and regenerative medicine	Dash et al. (2021)
Stimulus-responsive polymers	Encapsulation and conjugation	Controlled release and response to specific triggers (e.g., pH and temperature)	Potential complexity of design and limited understanding of *in* *vivo* behavior	Specific genes and mRNAs	Gene knockdown and silencing efficiency	Targeted drug delivery and gene therapy	Cancer treatment and inflammatory diseases	Arjama et al. (2018)
Nanogels	Encapsulation and conjugation	High biocompatibility, controlled release, and high loading capacity	Potential stability issues and limited targeting options	Specific genes and mRNAs	Gene knockdown and silencing efficiency	Drug delivery and gene therapy	Cancer treatment and genetic diseases	Yu et al. (2016a)
Polymeric micelles	Encapsulation and conjugation	High biocompatibility, controlled release, and improved stability compared to traditional micelles	Potential complexity of design and limited targeting options	Specific genes and mRNAs	Gene knockdown, silencing efficiency	Drug delivery and gene therapy	Cancer treatment and neurodegenerative diseases	Aseri et al. (2015)
Nanodiamonds	Conjugation and functionalization	High biocompatibility, unique optical properties, and long circulation time	Potential for aggregation and limited understanding of *in* *vivo* behavior	Specific genes and mRNAs	Gene silencing and cellular uptake	Drug delivery, imaging, and gene therapy	Cancer treatment and genetic disorders	Santana et al. (2022)
Calcium phosphate nanoparticles	Encapsulation and adsorption	High biocompatibility, biodegradability, and efficient cellular uptake	Potential for off-target effects and limited loading capacity	Specific genes and mRNAs	Gene silencing and cellular uptake	Gene therapy and vaccine development	Infectious diseases and genetic disorders	Ashique et al. (2023)
Silica nanoparticles	Encapsulation and conjugation	High biocompatibility, diverse functionalities, and ease of surface modification	Potential for aggregation and limited understanding of *in* *vivo* behavior	Specific genes and mRNAs	Gene silencing and cellular uptake	Drug delivery, imaging, and gene therapy	Cancer treatment and regenerative medicine	Hastings et al. (2015)

## 6 Clinical development of nanoparticles for gene delivery

Multifunctional nanoparticles have received considerable attention in preclinical studies related to gene delivery (Madl et al., 2012). The area of gene therapy, especially in the prevention and treatment of genetic diseases, has experienced a rapid increase in clinical trials worldwide (Pan et al., 2021). However, despite such progress, none of the gene therapeutics based on nanoparticles have yet gained approval from the FDA. This absence of approval shows the continuing difficulties and safety concerns linked to gene therapy (Gupta, 2020). A milestone in the clinical progress of gene therapy was achieved when Anderson et al. carried out the first human clinical trial, where the adenosine deaminase gene was systemically delivered to a 4-year-old girl suffering from severe combined immunodeficiency disease. The initial success of this case marked the beginning of a global expansion of research in gene therapy (Khan et al., 2023). Another important clinical trial was on severe combined immunodeficiency-X1. This inherited disorder, linked to the X chromosome, was treated using complementary DNA in a retrovirus-derived vector with *ex vivo* infection of CD34^+^ cells (Yadav P. et al., 2023). The 10-month follow-up showed encouraging outcomes, with T, B, and NK cell counts and their functions similar to those of age-matched healthy controls. Nevertheless, safety issues appeared later. After 3 years, the two youngest boys in the trial developed leukemia; this was mainly due to retrovirus vector integration near a proto-oncogene, which caused deregulated premalignant cell growth (Lan et al., 2020). This incident highlighted the safety risks of viral vectors and placed serious limits on the wide use of gene therapy (Tang et al., 2010). Despite these challenges, researchers continue to examine safer and more efficient strategies for gene delivery through multifunctional nanoparticles in preclinical studies, showing the active and developing character of this field (Yu et al., 2016b).

Nanoparticle-based gene therapy is currently under clinical evaluation for a wide range of diseases, using different delivery systems. One such approach is the use of polyethylenimine (PEI)-based nanoparticles. BC-819/PEI, developed by BioCanCell, is being studied for bladder cancer (BC) through local administration and is in phase 2 with active status (NCT00595088). In addition, BC-819, also from BioCanCell, has finished phase 1/2 clinical trials for ovarian cancer (OC) with intraperitoneal (IP) administration (NCT00826150). Another product, DTA-H19 by BioCanCell, has completed phase 1/2 trials for pancreatic neoplasms (PN) through local administration (NCT00711997).

Lipid-based nanoparticles represent another important system. TKM-080301, from Tekmira Pharmaceuticals Corporation, has completed phase 1 trials for hepatic metastases (HM) using intra-arterial (IA) administration (NCT01437007). Ongoing studies include hepatocellular carcinoma (HC) in phase 1/2, with active recruitment (NCT02191878), as well as completed phase 1/2 trials for neuroendocrine tumors (NET) and adrenocortical carcinoma (ACC) (NCT01262235). Another example is Atu027, developed by Silence Therapeutics GmbH, which has completed phase 1 testing for advanced solid cancer (ASC) using intravenous (IV) administration (NCT00938574). Similarly, ALN-TTR02, produced by Alnylam Pharmaceuticals for transthyretin amyloidosis (TTR-A), has completed phase 2 studies with IV administration (NCT01617967).

In addition, DOTAP-Chol-fus1 from the MD Anderson Cancer Center uses PLGA-based nanoparticles for lung cancer (LC) and has completed phase 1 testing with IV administration (NCT00059605). DCR-MYC, by Dicerna Pharmaceuticals, is being tested for solid tumors (ST), multiple myeloma (MM), and non–Hodgkin lymphoma (NHL). It is actively recruiting for both phase 1 (NCT02110563) and 1/2 (NCT02314052) studies, including hepatocellular carcinoma (HC) as one of the target conditions. PLGA-based nanoparticles are also used in siG12D LODER, a product of Silenseed, which is in phase 2 trials for pancreatic cancer (PC) via local administration (NCT01676259). Furthermore, Nitto Denko Corporation is conducting phase 1 studies for extensive hepatic fibrosis (EHF) with ND-L02-s0201 injection through IV administration (NCT02227459).

**FIGURE 6:**
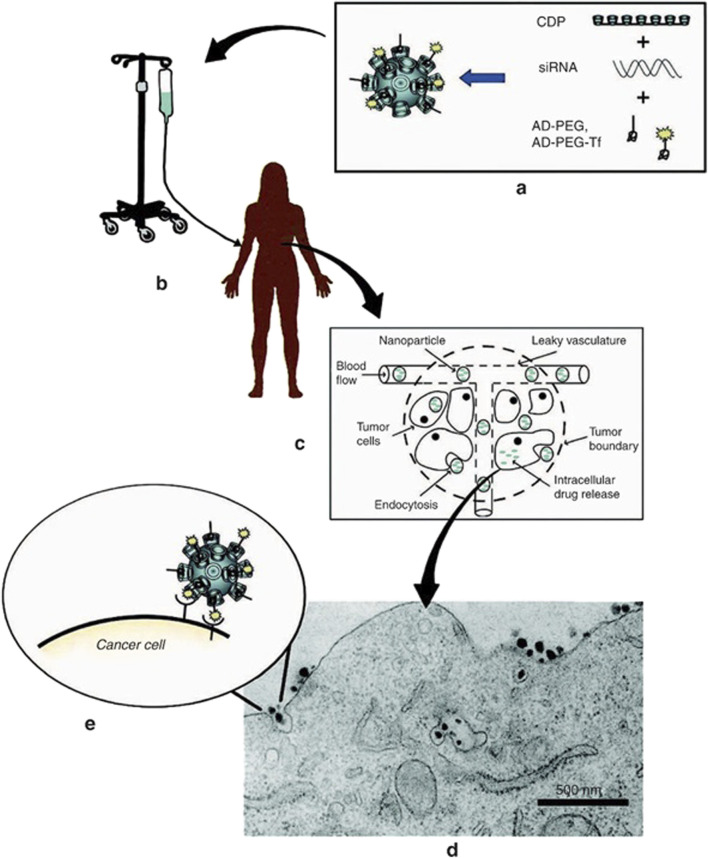
Proposed mechanism for CALAA-01. **(a)** Nanoparticles formed through the assembly of a linear polymer containing cyclodextrin (CDP), an adamantane-PEG conjugate (AD-PEG), a targeting element (transferrin, Tf), and the therapeutic gene (siRNA). **(b)** Patients receive an infusion of these nanoparticles. **(c)** Nanoparticles circulate and infiltrate tumors. **(d)** Receptor-mediated endocytosis occurs. **(e)** Targeted nanoparticles interact with receptors on the cancer cell surface. Reprinted from Davis (2009) with permission from American Chemical Society.

Continuing from the previous discussion, the successful performance of CALAA-01 in clinical trials has encouraged further investigation into nanoparticle-based gene delivery systems. Researchers are now examining different modifications and formulations in order to improve their efficiency and achieve more precise delivery to target sites. One important strategy is the optimization of nanoparticle composition to provide stronger stability and controlled release of therapeutic genes. In addition, researchers are attempting to refine targeting ligands so that they bind specifically to cancer cells, thus reducing off-target effects and improving the overall accuracy of treatment (Figure 6). Progress in nanotechnology has also supported the design of multifunctional nanoparticles that can transport several therapeutic agents at the same time. These complex systems are able to combine gene therapy with other treatment methods, including chemotherapy and immunotherapy, creating synergistic effects and improving therapeutic results. Such integration is highly valuable for addressing the complexity and heterogeneity of cancer, leading to more effective and individualized treatment options.

In recent years, investigators have also studied how the unique physicochemical features of nanoparticles may help overcome barriers in gene delivery, including the challenge of crossing the blood–brain barrier. This development has opened new opportunities for the treatment of neurological diseases through the targeted transport of therapeutic genes into specific regions of the brain. The adaptability of nanoparticles also makes it possible to design systems according to the needs of different diseases; this supports more tailored and efficient forms of gene therapy. As this field continues to expand, ongoing studies are working to resolve critical issues such as long-term safety, scalability, and production on a large scale. Cooperative work between researchers, clinicians, and industry representatives will play an essential role in turning these advances into practical, safe, and widely available treatments. The transition from laboratory research to clinical application of nanoparticle-based gene delivery systems shows great promise for transforming gene therapy and influencing the treatment of many diseases, particularly cancer. Table 5 provides an overview of the progress of clinical developments concerning nanoparticles used for gene delivery.

**TABLE 5:** Clinical development of nanoparticles for gene delivery.

Nanoparticle name/ID	Trial ID	Clinical phase	Gene delivery target	Delivery mechanism	Clinical trial status	Primary endpoint	Safety profile	Key findings/results
Onpattro (ALN-TTRsc)	NCT02006232	Phase III	TTR	Lipid nanoparticle	Completed	Reduction in serum TTR levels	Generally well-tolerated and mild injection site reactions reported	Significant reduction in serum TTR levels and improved patient outcomes in phase III trials
Glybera (alipogene tiparvovec)	NCT00803319	Approved	Lipoprotein lipase (LPL)	Adeno-associated viral vector (AAV)	Approved	Increase in plasma LPL activity	Limited side-effects reported and long-term safety data ongoing	First gene therapy approved in Europe for treatment of LPL deficiency
Kymriah (tisagenlecleucel)	NCT02122952	Approved	CAR T cells	Lentiviral vector	Approved	Event-free survival	Cytokine release syndrome and neurologic toxicities reported and manageable with supportive care	First CAR T-cell therapy approved in the US for the treatment of acute lymphoblastic leukemia
Luxturna (voretigene neparvovec-rzyl)	NCT00999993	Approved	RPE65	AAV	Approved	Improvement in visual function	Subretinal injection associated with infrequent ocular inflammation and long-term safety data ongoing	First gene therapy approved in the US for the treatment of Leber congenital amaurosis type 2
AADC-pDC	NCT03709185	Phase II	Aromatic L-amino acid decarboxylase (AADC)	AAV	Ongoing	Improvement in motor function	Generally well-tolerated and long-term safety data awaited	Early signs of motor improvement observed in phase I/II trials for the treatment of Parkinson’s disease
SG01	NCT04041524	Phase I	Connexin 26 (GJB2)	AAV	Ongoing	Improvement in hearing thresholds	Generally well-tolerated in early clinical trials for the treatment of hearing loss	Promising early results for restoration of hearing function
EVI-hPL	NCT03440284	Phase I/II	Hematopoietic progenitor stem cells (HPSCs)	Lentiviral vector	Ongoing	Engraftment and expansion of HPSCs	Early data suggest feasibility and safety and efficacy data pending	Potential treatment for various blood disorders using gene-modified HPSCs
Zolgensma (onasemnogene abeparvovec-xioi)	NCT03347586	Approved	SMN1	AAV	Approved	Event-free survival	Short-term safety data promising, long-term data ongoing	First gene therapy approved in the US for treatment of SMA type 1

## 7 Challenges and regulatory considerations

Nanomaterials in gene therapy present several challenges that must be carefully considered (Duan et al., 2021). A major difficulty is the possible toxicity of some nanomaterials (Dai et al., 2021). Because they possess unique physicochemical properties, nanoparticles may interact in unintended ways with biological systems, producing cytotoxic or immunogenic effects (Soroush et al., 2016). Understanding the toxicological profile of nanomaterials is therefore essential to guarantee the safety of gene therapy applications (QIAO et al., 2021). Another continuing challenge is the delivery efficiency of nanomaterials to the target cells (Chen et al., 2017). Achieving accurate delivery while reducing off-target effects is a complex process (Liang et al., 2023). The creation of efficient delivery systems that can pass biological barriers and reach the desired site of action remains a critical issue in nanomedicine (Sheikhzadeh et al., 2021). Moreover, the long-term stability of nanomaterials and their possible accumulation in tissues or organs require careful investigation (He and Zhao, 2020). This involves the study of biodistribution and clearance pathways to prevent adverse consequences linked with prolonged exposure (Ojha et al., 2023).

Regulatory aspects play a key role in ensuring the safe and ethical application of nanomaterials in gene therapy (Tay and Melosh, 2019). Authorities such as the Food and Drug Administration (FDA) and the European Medicines Agency (EMA) provide frameworks to evaluate the safety and effectiveness of therapies based on nanomaterials (Ghormade et al., 2011). Before starting clinical trials, researchers must satisfy regulatory requirements and submit detailed information on physicochemical properties, toxicity data, and manufacturing protocols (Shen Y. et al., 2018). This process ensures that potential risks and benefits are fully examined before human studies begin (Li et al., 2017). Ethical issues are also central to the regulatory environment (Kang et al., 2017). Studies involving human participants must go through strict ethical reviews to protect the health and rights of subjects (Yang et al., 2023). This requires obtaining informed consent, maintaining privacy, and guaranteeing transparency in research activities (Khaliq et al., 2023). Post-market surveillance and continuous monitoring are further important components of regulation (Han et al., 2021). Ongoing assessments after approval allow the detection of new safety problems and enable agencies to respond to protect public health (Szczeszak et al., 2015).

Addressing off-target effects in nanomaterial-based gene therapy requires multiple strategies (Agapito-Tenfen, 2016). One important method is designing nanocarriers with better specificity (Vélez et al., 2017). Surface modifications of nanoparticles can be used to improve their binding to target cells and reduce non-specific interactions (Zia et al., 2021). Advanced imaging technologies are also necessary to monitor biodistribution and pharmacokinetics of nanomaterials in real time (Flores et al., 2012). These techniques allow the detection of off-target accumulation and enable adjustments to improve delivery accuracy (Kapoor et al., 2018). The addition of intelligent targeting systems, such as ligands or antibodies, can further improve selectivity for certain cell types (Roma-rodrigues et al., 2020). These molecules bind to receptors on target cells, supporting accurate delivery and lowering off-target risks (Kumar et al., 2021). Furthermore, controlling the size and shape of nanomaterials can influence circulation time and uptake by cells (Haque et al., 2021). Adjusting these factors improves nanocarrier performance and reduces unwanted interactions (Salman et al., 2022). Preclinical investigations, both *in vitro* and *in vivo*, are necessary to assess safety and efficacy (Liang et al., 2023). These studies allow researchers to recognize off-target effects at early stages (Yang et al., 2015).

Reducing immunogenic responses in nanomaterial-based gene therapy requires a deep understanding of how nanoparticles interact with the immune system (Wang et al., 2018). One possible approach is to design nanomaterials with immunomodulatory characteristics to lower inflammation and encourage immune tolerance (Shen et al., 2021). Modifying the surface of nanocarriers can also decrease immune recognition (Pillai, 2014). For instance, PEGylation, in which nanoparticles are coated with polyethylene glycol, lowers immunogenicity and increases circulation time (Yang et al., 2015). Choosing biocompatible materials such as lipids or biodegradable polymers is equally important (Shen et al., 2022). Such materials are tolerated by the immune system and can limit harmful reactions (Pezoa et al., 2022). Comprehensive immunotoxicity testing in preclinical studies is essential to detect risks and develop effective countermeasures (Feng et al., 2023). Such evaluations include the effects of nanomaterials on immune cells, cytokine release, and immune balance (Fiaz et al., 2021). Finally, applying immune evasion methods, such as camouflage or combining with immunosuppressive agents, can improve the stealth features of nanomaterials and decrease immune detection (Samarasinghe et al., 2012).

The ethical application of nanomaterials in gene therapy requires careful attention to several main principles (Tian et al., 2013). Informed consent is essential to ensure that participants in clinical trials have a full understanding of the intervention, including the possible risks and benefits (Doroftei et al., 2021). Clear and open communication is necessary to maintain trust and respect for the autonomy of participants (Lai et al., 2017). Another important ethical principle is the protection of privacy (Yuan et al., 2023). Researchers need to apply strong data security systems to protect sensitive information collected during gene therapy trials (Khan, 2019). The use of anonymized data and strict confidentiality rules helps respect the privacy rights of participants (Foley et al., 2022).

Equitable access to gene therapies is also a central ethical concern (Ferdous et al., 2022). It is important to ensure that the benefits of nanomaterial-based gene therapy are available to all populations without discrimination (Kavanagh and Green, 2022). This includes solving problems of cost, availability, and inclusion in clinical trials, which are necessary parts of an ethical framework (Edagwa et al., 2017). Researchers must also reflect on the wider social impacts of gene therapy (Zheng et al., 2021). Considering and reducing possible negative outcomes, such as genetic discrimination or greater social inequalities, is essential (Hur and Chung, 2021). Ethical frameworks should guide all decisions to reduce harm and support social justice (Yang TC. et al., 2020). Ongoing ethical oversight, from the early laboratory stage to monitoring after approval, is also necessary (Majidi et al., 2016). Ethical review boards and regulatory bodies have an important role in protecting ethical standards and ensuring the responsible use of nanomaterials in gene therapy (Wang et al., 2015).

It is also important to flexibly adapt regulatory systems to the rapid progress of nanomaterial-based therapies (Xu et al., 2022). One key method is creating interdisciplinary cooperation between regulatory bodies, scientists, industry partners, and ethicists (Pissuwan et al., 2011; Zhu et al., 2022; Shen L. et al., 2018). This cooperation allows knowledge sharing, real-time risk assessment, and a broader understanding of the field (Erel-Akbaba et al., 2021). The regular updating of regulatory guidelines is necessary for including new scientific evidence and technological progress (Lai et al., 2017). Monitoring scientific studies, joining international collaborations, and working with expert panels can inform regulators about the latest advances in nanomedicine and gene therapy (Lin et al., 2015). Special regulatory pathways for nanomaterial-based therapies can also make the approval process more efficient (Han et al., 2021). Recognizing the unique opportunities and risks of nanotechnology helps the design of specific regulations that encourage innovation while protecting public health (Casper et al., 2023). Furthermore, promoting openness and communication between regulators and industry is essential (Kausar, 2021). Open discussion supports information exchange, helps achieve compliance, and improves responses to new challenges in nanomaterial-based gene therapies (Corte et al., 2019).

Incorporating adaptive licensing strategies, including conditional approvals and post-marketing monitoring, can address the dynamic development of nanomedicine (Ferdous et al., 2022). These strategies create a structure for gathering real-world data, revising risk–benefit evaluations, and making decisions that reflect advancing scientific knowledge (Calos, 2017). International cooperation is necessary to build consistent regulations and guarantee the safe and ethical application of nanomaterial-based gene therapies (Haque et al., 2021). Regulatory authorities from different regions can collaborate to harmonize guidelines, exchange best practices, and jointly respond to the worldwide challenges of this new field (van de Wiel et al., 2017). An effective method is the formation of international working groups or task forces devoted to nanotechnology in gene therapy (Feng et al., 2018). These groups bring experts from various agencies to evaluate scientific progress, exchange information, and design shared frameworks for testing nanomaterial safety and effectiveness (Zych et al., 2018). Participation in global conferences and workshops on nanomedicine and gene therapy also ensures that regulators remain informed about scientific developments (Maity et al., 2023). Such platforms encourage cooperation, networking, and the creation of international partnerships that strengthen regulatory coordination (Agapito-Tenfen, 2016).

The use of shared terminology and definitions for nanomaterials in gene therapy is essential (Su et al., 2016). Harmonization improves clarity in communication between regulators, scientists, and industry representatives (Lamboro et al., 2021). Open data exchange and the sharing of research findings promote transparency and strengthen consistent regulatory decisions (Gogolev et al., 2021). Creating a shared database or repository for nanomaterial safety and effectiveness supports the development of international standards (Ansari et al., 2020). Close cooperation between industry and regulatory authorities is also necessary for smoother approval processes (Zhou et al., 2022). Establishing clear communication and constructive relations promotes the exchange of information, ensures compliance, and accelerates the approval of innovative therapies (Gao et al., 2017). Early and transparent communication with regulators is particularly important (Joun et al., 2022). Companies should actively seek advice and feedback during preclinical and clinical development (Rong et al., 2020). This process helps align study designs, outcomes, and expectations, thus reducing the risk of delays in approval (Ibrahim et al., 2022). Involvement in regulatory pilot projects on nanomaterials in gene therapy provides practical insights and helps refine specialized approaches (Turcheniuk and Mochalin, 2017a). Such initiatives allow regulators and industry to test and improve methods designed for nanotechnology (Foley et al., 2022).

Strong and transparent data generation is also vital (Ding et al., 2022). Industry actors must conduct broad preclinical and clinical research, presenting regulators with detailed evidence on safety, quality, and effectiveness (Ghormade et al., 2011). This practice builds trust in the review process and speeds approvals (Koupaei et al., 2019). A risk-based regulatory approach also ensures an efficient use of resources (Kozielski et al., 2014). By prioritizing actions according to the level of risk linked to different aspects of nanomaterial-based therapies, stakeholders can create a more focused regulatory plan (Morán et al., 2018). At the same time, the development of international ethical standards for nanomaterials in gene therapy requires cooperation from global stakeholders (Wang et al., 2017). Agreement on ethical principles should involve experts, regulators, ethicists, researchers, and industry (Liang et al., 2023). Organizations such as the World Health Organization (WHO) and UNESCO may lead these efforts by coordinating ethical guidelines (Liu et al., 2018). Expert panels and working groups can specifically address the ethical dimensions of nanomaterial-based gene therapies to ensure inclusive solutions (Ferdous et al., 2022).

Public dialogue and engagement remain critical for including diverse perspectives in ethical standards (Edagwa et al., 2017). Ethical questions in gene therapy go beyond scientific or regulatory concerns since they involve social values and cultural contexts (Calos, 2017). Public consultations increase transparency and ensure that guidelines reflect global perspectives (Haque et al., 2021). A continuous review system is needed to respond to scientific and societal change (Feng et al., 2018). Updating ethical standards regularly guarantees their relevance and suitability for new developments (Turcheniuk and Mochalin, 2017a). International collaboration with bioethics organizations can also strengthen expertise and guidance (Rong et al., 2020). Aligning ethical frameworks with existing agreements and conventions helps create a unified global approach to the responsible use of nanomaterials in gene therapy (Shen et al., 2021).

## 8 Outlook and future research directions

Recent developments in nanomaterials for gene therapy focus on using different nanoparticles, such as liposomes, polymeric nanoparticles, and inorganic nanoparticles, to deliver genetic material into target cells (Flores et al., 2012). These nanomaterials act as carriers for therapeutic genes, supporting targeted and regulated gene expression (Gogolev et al., 2021). Liposomes are widely used because of their biocompatibility and ability to enclose both hydrophobic and hydrophilic gene loads (Kavanagh and Green, 2022). Polymeric nanoparticles provide design flexibility and allow precise control over release patterns (Haque et al., 2021). Inorganic nanoparticles, including gold and silica, are studied for their special features, such as surface modification to improve targeting and regulate release (Demirer et al., 2021). The field of nanomaterials in gene therapy is advancing quickly due to progress in materials science and biotechnology (Kaur et al., 2023). Current studies aim to create new materials with higher biocompatibility, lower toxicity, and better stability (Padayachee and Singh, 2020). Smart materials, including stimulus-responsive polymers, enable the controlled release of genetic material in reaction to specific physiological signals, improving therapeutic outcomes (Maity et al., 2023). Multifunctional nanomaterials that combine diagnostic and therapeutic functions are also attracting attention, supporting more precise and personalized gene therapy (Gad et al., 2020). Future directions in this area include innovative delivery strategies, such as cell-specific targeting and tissue-specific accumulation (Su et al., 2016). Developing nanomaterials for oral gene delivery is another important focus; it aims to solve difficulties linked to systemic administration (Xie et al., 2021). Furthermore, combining advanced imaging technologies with nanomaterials may allow the real-time monitoring of gene delivery, giving deeper insights into treatment effectiveness and possible side effects (Gogolev et al., 2021). These new trends in nanomaterials strongly influence gene therapy by improving accuracy, efficiency, and safety (Ansari et al., 2020). Creating nanomaterials with better pharmacokinetics and lower immune response improves the overall success of therapy (Zhou et al., 2022). Designing nanocarriers with particular properties, such as surface charge and size, also affects their interactions with biological barriers, influencing outcomes (Salman et al., 2022). The expected impact of nanomaterials in gene therapy in the near future is significant (Živojević et al., 2021). Nanocarriers may solve existing barriers, such as cell uptake, stability, and immune reactions (Song et al., 2023). Personalized nanotherapeutics that fit individual genetic profiles may soon become possible, opening the way to more targeted and effective treatments (Ibrahim et al., 2022). As the field grows, applying artificial intelligence (AI) and machine learning to improve delivery systems is expected to increase precision and efficacy (Miller and Siegwart, 2018). The progress of nanomaterials in gene therapy shows great potential to transform the field (Lastochkina et al., 2022). Present trends highlight the diversity of nanomaterials, from liposomes to inorganic nanoparticles, used in the controlled delivery of therapeutic genes (Wu K. et al., 2022). The continuous progress of nanomaterials, with better compatibility, reduced toxicity, and creative design, demonstrates the dynamic nature of this research (Xi et al., 2022). Future studies will likely emphasize advanced strategies, including oral delivery and cell-specific targeting, to address current challenges (Shen et al., 2021). New directions not only improve efficiency but also support multifunctional nanomaterials with diagnostic features (Kozielski et al., 2014). Looking ahead, nanomaterials are expected to bring transformative changes to gene therapy, making personalized treatment and AI-driven optimization possible (Mohamad et al., 2023). It is clear that nanomaterials will play a central role in shaping the future of gene therapy, with effects reaching far beyond the present state of the art (Wang et al., 2017).

## References

[B1] Abinaya R. V. Viswanathan P. (2021). Biotechnology-based therapeutics. Transl Biotechnol A Journey Lab Clin, 27–52. 10.1016/b978-0-12-821972-0.00019-8

[B2] Agapito-Tenfen S. Z. (2016). Biosafety aspects of genome-editing techniques. Biosaf. Brief. Third World Netw., 11.

[B3] Alamri H. R. Rezk H. Abd-Elbary H. Ziedan H. A. Elnozahy A. (2020). Experimental investigation to improve the energy effciency of solar PV panels using hydrophobic SiO_2_ nanomaterial. Coatings 10.

[B4] Alsulami M. N. (2021). Eradication of malaria: present situations and new strategies. J. Pharm. Res. Int., 17–37. 10.9734/jpri/2021/v33i47a32986

[B5] Amreddy N. Babu A. Muralidharan R. Munshi A. Ramesh R. (2017). Polymeric nanoparticle-mediated gene delivery for lung cancer treatment. Top. Curr. Chem., 233–255. 10.1007/978-3-319-77866-2_9 PMC5480422 28290155

[B6] Ansari W. A. Chandanshive S. U. Bhatt V. Nadaf A. B. Vats S. Katara J. L. (2020). Genome editing in cereals: approaches, applications and challenges. Int. J. Mol. Sci. 21, 1–32. 10.3390/ijms21114040 32516948 PMC7312557

[B7] Anwar A. Mohammed B. S. Wahab M. A. Liew M. S. (2020). Enhanced properties of cementitious composite tailored with graphene oxide nanomaterial - a review. Dev. Built Environ. 1, 100002. 10.1016/j.dibe.2019.100002

[B8] Arjama M. Mehnath S. Rajan M. Jeyaraj M. (2018). Sericin/RBA embedded gellan gum based smart nanosystem for pH responsive drug delivery. Int. J. Biol. Macromol. 120, 1561–1571. 10.1016/j.ijbiomac.2018.09.146 30261261

[B9] Aseri A. Garg S. K. Nayak A. Trivedi S. K. Ahsan J. (2015). Magnetic nanoparticles: magnetic nano-technology using biomedical applications and future prospects. Int. J. Pharm. Sci. Rev. Res., 119–131.

[B10] Ashique S. Garg A. Mishra N. Raina N. Ming L. C. Tulli H. S. (2023). Nano-mediated strategy for targeting and treatment of non-small cell lung cancer (NSCLC). Naunyn. Schmiedeb. Arch. Pharmacol. 396, 2769–2792. 10.1007/s00210-023-02522-5 37219615

[B11] Ashish P. K. Singh D. (2021). Use of nanomaterial for asphalt binder and mixtures: a comprehensive review on development, prospect, and challenges. Road. Mater Pavement Des. 22, 492–538. 10.1080/14680629.2019.1634634

[B12] Aziz A. Rehman U. Sheikh A. Abourehab M. A. S. Kesharwani P. (2023). Lipid-based nanocarrier mediated CRISPR/Cas9 delivery for cancer therapy. J. Biomater. Sci. Polym. Ed. 34, 398–418. 10.1080/09205063.2022.2121592 36083788

[B13] Åström K. J. Kumar PNPR Adams M. P. Fu T. Cabrera A. G. Morales M. (2015). Abstracts. J. Power Sources 1.

[B14] Banerjee K. Pramanik P. Maity A. Joshi D. C. Wani S. H. Krishnan P. (2019). Methods of using nanomaterials to plant systems and their delivery to plants (mode of entry, uptake, translocation, accumulation, biotransformation and barriers). Adv Phyt. Synth Appl, 123–152. 10.1016/b978-0-12-815322-2.00005-5

[B15] Barhoum A. Jeevanandam J. Danquah M. K. (2022). Fundamentals of bionanomaterials. Fundam. Bionanomaterials.

[B16] Barua S. Geng X. Chen B. (2020). Graphene-based nanomaterials for healthcare applications. Photonanotechnol. Ther. Imaging, 45–81. 10.1016/b978-0-12-817840-9.00003-5

[B17] Besenhard M. O. Pal S. Gkogkos G. Gavriilidis A. (2023). Non-fouling flow reactors for nanomaterial synthesis. React. Chem. Eng. 8, 955–977. 10.1039/d2re00412g

[B18] Bokov D. Turki Jalil A. Chupradit S. Suksatan W. Javed Ansari M. Shewael I. H. (2021). Nanomaterial by sol-gel method: synthesis and application. Adv. Sci. Eng. 2021, 5102014. 10.1155/2021/5102014

[B19] Bora T. Dousse A. Sharma K. Sarma K. Baev A. Hornyak G. L. (2019). Modeling nanomaterial physical properties: theory and simulation. Int. J. Smart Nano Mater 10, 116–143. 10.1080/19475411.2018.1541935

[B20] Boverhof D. R. Bramante C. M. Butala J. H. Clancy S. F. Lafranconi W. M. West J. (2015). Comparative assessment of nanomaterial definitions and safety evaluation considerations. Regul. Toxicol. Pharmacol. 73, 137–150. 10.1016/j.yrtph.2015.06.001 26111608

[B21] Brüngel R. Rückert J. Müller P. Babick F. Friedrich C. M. Ghanem A. (2023). NanoDefiner framework and e-Tool revisited according to the European Commission’s nanomaterial definition 2022/C 229/01. Nanomaterials 13, 990. 10.3390/nano13060990 36985884 PMC10056892

[B22] Buschmann M. D. Carrasco M. J. Alishetty S. Paige M. Alameh M. G. Weissman D. (2021). Nanomaterial delivery systems for MRNA vaccines. Vaccines. 9, 1–30. 10.3390/vaccines9010065 33478109 PMC7836001

[B23] Cai X. J. Xu Y. Y. (2011). Nanomaterials in controlled drug release. Cytotechnology 63, 319–323. 10.1007/s10616-011-9366-5 21720796 PMC3140842

[B24] Calos M. P. (2017). Genome editing techniques and their therapeutic applications. Clin. Pharmacol. Ther. 101, 42–51. 10.1002/cpt.542 27783398

[B25] Campuzano S. Pingarrón J. M. (2023). Electrochemical affinity biosensors: pervasive devices with exciting alliances and Horizons ahead. ACS Sensors 8, 3276–3293. 10.1021/acssensors.3c01172 37534629 PMC10521145

[B26] Casper J. Nicolle L. Willimann M. Eü K. Tran A. Robin P. (2023). Core–shell structured chitosan-polyethylenimine nanoparticles for gene delivery: improved stability, cellular uptake, and transfection efficiency. Macromol. Biosci. 23, 2200314. 10.1002/mabi.202200314 36200651

[B27] Chen J. Guo Z. Tian H. Chen X. (2016). Production and clinical development of nanoparticles for gene delivery. Mol. Therapy-Methods and Clin. Dev. 3, 16023. 10.1038/mtm.2016.23 27088105 PMC4822651

[B28] Chen L. Simpson J. D. Fuchs A. V. Rolfe B. E. Thurecht K. J. (2017). Effects of surface charge of hyperbranched polymers on cytotoxicity, dynamic cellular uptake and localization, hemotoxicity, and pharmacokinetics in mice. Mol. Pharm. 14, 4485–4497. 10.1021/acs.molpharmaceut.7b00611 29116801

[B29] Chen J. Ma Q. Wu X. J. Li L. Liu J. Zhang H. (2019). Wet-chemical synthesis and applications of semiconductor nanomaterial-based epitaxial heterostructures. Nano-Micro Lett. 11, 86. 10.1007/s40820-019-0317-6 34138028 PMC7770813

[B30] Chen Y. Aslanoglou S. Murayama T. Gervinskas G. Fitzgerald L. I. Sriram S. (2020). Silicon-nanotube-mediated intracellular delivery enables *Ex Vivo* gene editing. Adv. Mater. 32, 2000036. 10.1002/adma.202000036 32378244

[B31] Cheng X. Xie Q. Sun Y. (2023). Advances in nanomaterial-based targeted drug delivery systems. Front. Bioeng. Biotechnol. 11, 1177151. 10.3389/fbioe.2023.1177151 37122851 PMC10133513

[B32] Chenouard V. Leray I. Tesson L. Remy S. Allan A. Archer D. (2023). Excess of guide RNA reduces knockin efficiency and drastically increases on-target large deletions. iScience 26, 106399. 10.1016/j.isci.2023.106399 37034986 PMC10074149

[B33] Chi H. Liu G. (2023). Carbon nanomaterial-based molecularly imprinted polymer sensors for detection of hazardous substances in food: recent progress and future trends. Food Chem. 420, 136100. 10.1016/j.foodchem.2023.136100 37062085

[B34] Chin J. S. Chooi W. H. Wang H. Ong W. Leong K. W. Chew S. Y. (2019). Scaffold-mediated non-viral delivery platform for CRISPR/Cas9-based genome editing. Acta Biomater. 90, 60–70. 10.1016/j.actbio.2019.04.020 30978509

[B35] Cho E. Y. Ryu J. Y. Lee H. A. R. Hong S. H. Park H. S. Hong K. S. (2019). Lecithin nano-liposomal particle as a CRISPR/Cas9 complex delivery system for treating type 2 diabetes. J. Nanobiotechnology 17, 19. 10.1186/s12951-019-0452-8 30696428 PMC6350399

[B36] Chu Z. Peng J. Jin W. (2017). Advanced nanomaterial inks for screen-printed chemical sensors. Sensors Actuators, B Chem. 243, 919–926. 10.1016/j.snb.2016.12.022

[B37] Corte L. E. D. Mahmoud L. M. Moraes T. S. Mou Z. Grosser J. W. Dutt M. (2019). Development of improved fruit, vegetable, and ornamental crops using the CRISPR/cas9 genome editing technique. Plants 8, 601. 10.3390/plants8120601 31847196 PMC6963220

[B38] Crowley J. S. Liu A. Dobke M. (2021). Regenerative and stem cell-based techniques for facial rejuvenation. Exp. Biol. Med. 246, 1829–1837. 10.1177/15353702211020701 34102897 PMC8381699

[B39] Dai Z. Xu X. Guo Z. Zheng K. Song X. Z. Qi X. (2021). Effect of ROS generation on highly dispersed 4-layer O-Ti7O13 nanosheets toward tumor synergistic therapy. Mater Sci. Eng. C 120, 111666. 10.1016/j.msec.2020.111666 33545831

[B40] Dannhäuser S. Mrestani A. Gundelach F. Pauli M. Komma F. Kollmannsberger P. (2022). Endogenous tagging of Unc-13 reveals nanoscale reorganization at active zones during presynaptic homeostatic potentiation. Front. Cell Neurosci. 16, 1074304. 10.3389/fncel.2022.1074304 36589286 PMC9797049

[B41] Dasari Shareena T. P. McShan D. Dasmahapatra A. K. Tchounwou P. B. (2018). A review on graphene-based nanomaterials in biomedical applications and risks in environment and health. Nano-Micro Lett. 10, 53. 10.1007/s40820-018-0206-4 30079344 PMC6075845

[B42] Dash B. S. Lu Y. J. Chen H. A. Chuang C. C. Chen J. P. (2021). Magnetic and GRPR-targeted reduced graphene oxide/doxorubicin nanocomposite for dual-targeted chemo-photothermal cancer therapy. Mater Sci. Eng. C 128, 112311. 10.1016/j.msec.2021.112311 34474862

[B43] Davis M. E. (2009). The first targeted delivery of siRNA in humans *via* a self-assembling, cyclodextrin polymer-based nanoparticle: from concept to clinic. Mol. Pharm. 6 (3), 659–668. 10.1021/mp900015y 19267452

[B44] Demirer G. S. Silva T. N. Jackson C. T. Thomas J. B. Ehrhardt D W. Rhee S. Y. (2021). Nanotechnology to advance CRISPR–Cas genetic engineering of plants. Nat. Nanotechnol. 16, 243–250. 10.1038/s41565-021-00854-y 33712738 PMC10461802

[B314] Deng H. Huang W. Zhang Z. (2019). Nanotechnology based CRISPR/Cas9 system delivery for genome editing: progress and prospect. Nano Res. 12, 2437–2450. 10.1007/s12274-019-2465-x

[B45] Dinari A. Moghadam T. T. Abdollahi M. Sadeghizadeh M. (2018). Synthesis and characterization of a nano-polyplex system of GNRs-PDMAEA-pDNA: an inert self-catalyzed degradable carrier for facile gene delivery. Sci. Rep. 8, 8112. 10.1038/s41598-018-26260-4 29802331 PMC5970195

[B46] Ding H. Zhang J. Zhang F. Xu Y. Liang W. Yu Y. (2022). Nanotechnological approaches for diagnosis and treatment of ovarian cancer: a review of recent trends. Drug Deliv. 29, 3218–3232. 10.1080/10717544.2022.2132032 36259505 PMC9586634

[B47] Dodds W. J. (2023). One health: animal models of heritable human bleeding diseases. Animals 13, 87. 10.3390/ani13010087 36611696 PMC9818017

[B48] Doroftei B. Ilie O. D. Puiu M. Ciobica A. Ilea C. (2021). Mini-review regarding the applicability of genome editing techniques developed for studying infertility. Diagnostics 11, 246. 10.3390/diagnostics11020246 33562517 PMC7915733

[B49] Duan L. Ouyang K. Xu X. Xu L. Wen C. Zhou X. (2021). Nanoparticle delivery of CRISPR/Cas9 for genome editing. Front. Genet. 12, 673286. 10.3389/fgene.2021.673286 34054927 PMC8149999

[B50] Dubey N. Dhiman S. Koner A. L. (2023). Review of carbon dot-based drug conjugates for cancer therapy. ACS Appl. Nano Mater. 6, 4078–4096. 10.1021/acsanm.2c05407

[B51] Dufresne A. (2017). Cellulose nanomaterial reinforced polymer nanocomposites. Curr. Opin. Colloid Interface Sci. 29, 1–8. 10.1016/j.cocis.2017.01.004

[B52] Edagwa B. McMillan J. E. Sillman B. Gendelman H. E. (2017). Long-acting slow effective release antiretroviral therapy. Expert Opin. Drug Deliv. 14, 1281–1291. 10.1080/17425247.2017.1288212 28128004 PMC5545165

[B53] Eichhorn S. J. Etale A. Wang J. Berglund L. A. Li Y. Cai Y. (2022). Current international research into cellulose as a functional nanomaterial for advanced applications. J. Mater. Sci. 57, 5697–5767. 10.1007/s10853-022-06903-8

[B54] Erel-Akbaba G. Isar S. Akbaba H. (2021). Development and evaluation of solid witepsol nanoparticles for gene delivery. Turk. J. Pharm. Sci. 18, 344–351. 34157825 10.4274/tjps.galenos.2020.68878 PMC8231323

[B55] Evans B. C. Fletcher R. B. Kilchrist K. V. Dailing E. A. Mukalel A. J. Colazo J. M. (2019). An anionic, endosome-escaping polymer to potentiate intracellular delivery of cationic peptides, biomacromolecules, and nanoparticles. Nat. Commun. 10, 5012. 10.1038/s41467-019-12906-y 31676764 PMC6825215

[B56] Falzarano M. S. Scotton C. Passarelli C. Ferlini A. (2015). Duchenne muscular dystrophy: from diagnosis to therapy. Molecules 20, 18168–18184. 10.3390/molecules201018168 26457695 PMC6332113

[B57] Feng X. Zhao D. Zhang X. Ding X. Bi C. (2018). CRISPR/Cas9 assisted multiplex genome editing technique in *Escherichia coli* . Biotechnol. J. 13, 1700604. 10.1002/biot.201700604 29790644

[B58] feng Li D. song L. Q. feng Y. M. ming Xu H. Zhu M. zheng Zhang Y. (2023). Nanomaterials for mRNA-based therapeutics: challenges and opportunities. Bioeng. Transl. Med. 8, e10492. 10.1002/btm2.10492 37206219 PMC10189457

[B59] Ferdous M. A. Islam S. I. Habib N. Almehmadi M. Allahyani M. Alsaiari A. A. (2022). CRISPR-Cas genome editing technique for fish disease management: current study and future perspective. Microorganisms 10, 2012. 10.3390/microorganisms10102012 36296288 PMC9610719

[B60] Fernando I. P. S. Lee W. W. Han E. J. Ahn G. (2020). Alginate-based nanomaterials: fabrication techniques, properties, and applications. Chem. Eng. J. 391, 123823. 10.1016/j.cej.2019.123823

[B61] Fiaz S. Ahmar S. Saeed S. Riaz A. Mora-Poblete F. Jung K. H. (2021). Evolution and application of genome editing techniques for achieving food and nutritional security. Int. J. Mol. Sci. 22, 5585. 10.3390/ijms22115585 34070430 PMC8197453

[B62] Flores K. J. Craig M. Wanekaya A. Dong L. Ghosh K. Smith J. J. (2012). Tipping the proteome with gene-based vaccines: weighing in on the role of nanomaterials. J. Nanotechnol. 2012, 1–9. 10.1155/2012/843170

[B63] Foley R. A. Sims R. A. Duggan E. C. Olmedo J. K. Ma R. Jonas S. J. (2022). Delivering the CRISPR/Cas9 system for engineering gene therapies: recent cargo and delivery approaches for clinical translation. Front. Bioeng. Biotechnol. 10, 973326. 10.3389/fbioe.2022.973326 36225598 PMC9549251

[B64] Fu H. You Y. Wang S. Chang H. (2022). SnO_2_ nanomaterial coating micro-fiber interferometer for ammonia concentration measurement. Opt. Fiber Technol. 68, 102819. 10.1016/j.yofte.2022.102819

[B65] Gad M. A. Li M. Ahmed F. K. Almoammar H. (2020). Nanomaterials for gene delivery and editing in plants: challenges and future perspective. Multifunct. Hybrid. Nanomater Sustain Agri-Food Ecosyst., 135–153. 10.1016/b978-0-12-821354-4.00006-6

[B66] Gao X. Zhai M. Guan W. Liu J. Liu Z. Damirin A. (2017). Controllable synthesis of a smart multifunctional nanoscale metal-organic framework for magnetic resonance/optical imaging and targeted drug delivery. ACS Appl. Mater Interfaces 9, 3455–3462. 10.1021/acsami.6b14795 28079361

[B67] Gao H. Ran Q. Hu X. Zhu Q. (2021). DNA-free genome editing. Kexue Tongbao/Chinese Sci. Bull. 66, 1408–1422. 10.1360/tb-2020-0891

[B68] Gbian D. L. Omri A. (2021). Current and novel therapeutic strategies for the management of cystic fibrosis. Expert Opin. Drug Deliv. 18, 535–552. 10.1080/17425247.2021.1874343 33426936

[B69] Ge L. Mu X. Tian G. Huang Q. Ahmed J. Hu Z. (2019). Current applications of gas sensor based on 2-D nanomaterial: a mini review. Front. Chem. 7, 839. 10.3389/fchem.2019.00839 31921765 PMC6914763

[B70] Ghormade V. Deshpande M. V. Paknikar K. M. (2011). Perspectives for nano-biotechnology enabled protection and nutrition of plants. Biotechnol. Adv. 29, 792–803. 10.1016/j.biotechadv.2011.06.007 21729746

[B71] Giordani S. (2020). (Invited) carbon nano onions for biomedical applications. ECS Meet. Abstr. MA2020-01, 653. 10.1149/ma2020-016653mtgabs

[B72] Gogolev Y. V. Ahmar S. Akpinar B. A. Budak H. Kiryushkin A. S. Gorshkov V. Y. (2021). Omics, epigenetics, and genome editing techniques for food and nutritional security. Plants 10, 1423. 10.3390/plants10071423 34371624 PMC8309286

[B73] Gong W. Zhang T. Che M. Wang Y. He C. Liu L. (2023). Recent advances in nanomaterials for the treatment of spinal cord injury. Mater. Today Bio. 18, 100524. 10.1016/j.mtbio.2022.100524 36619202 PMC9813796

[B74] González-Reyna M. A. Molina G. A. Juarez-Moreno K. Rodríguez-Torres A. Esparza R. Estevez M. (2023). Green nanoarchitectonics of carbon quantum dots from Cinchona Pubescens Vahl as targeted and controlled drug cancer nanocarrier. Biomater. Adv. 153, 213561. 10.1016/j.bioadv.2023.213561 37515841

[B75] Gupta S. K. (2020). Study of nanotechnology and its application. J. Phys. Opt. Sci., 1–7. 10.47363/jpsos/2020(2)107

[B76] Hamid A. Salee S. (2022). Role of nanoparticles in management of plant pathogens and scope in plant transgenics for imparting disease resistance. Plant Prot. Sci. 58, 173–184. 10.17221/37/2020-pps

[B77] Han S. Lee Y. Lee M. (2021). Biomimetic cell membrane-coated DNA nanoparticles for gene delivery to glioblastoma. J. Control Release 338, 22–32. 10.1016/j.jconrel.2021.08.021 34391836

[B78] Han B. Song Y. Park J. Doh J. (2022). Nanomaterials to improve cancer immunotherapy based on *ex vivo* engineered T cells and NK cells. J. Control. Release. 343, 379–391. 10.1016/j.jconrel.2022.01.049 35124129

[B79] Hao B. Wei L. Cheng Y. Ma Z. Wang J. (2022). Advanced nanomaterial for prostate cancer theranostics. Front. Bioeng. Biotechnol. 10, 1046234. 10.3389/fbioe.2022.1046234 36394009 PMC9663994

[B80] Haque M. A. Rafii M. Y. Yusoff M. M. Ali N. S. Yusuff O. Datta D. R. (2021). Advanced breeding strategies and future perspectives of salinity tolerance in rice. Agronomy 11, 1631. 10.3390/agronomy11081631

[B81] Hastings J. Jeliazkova N. Owen G. Tsiliki G. Munteanu C. R. Steinbeck C. (2015). eNanoMapper: harnessing ontologies to enable data integration for nanomaterial risk assessment. J. Biomed. Semant. 6, 10. 10.1186/s13326-015-0005-5 25815161 PMC4374589

[B82] He Y. Zhao Y. (2020). Technological breakthroughs in generating transgene-free and genetically stable CRISPR-edited plants. aBIOTECH 1, 88–96. 10.1007/s42994-019-00013-x 36305007 PMC9584093

[B83] He X. Deng H. Hwang H. (2019). The current application of nanotechnology in food and agriculture. J. Food Drug Anal. 27, 1–21. 10.1016/j.jfda.2018.12.002 30648562 PMC9298627

[B84] Höijer I. Johansson J. Gudmundsson S. Chin C. S. Bunikis I. Häggqvist S. (2020). Amplification-free long-read sequencing reveals unforeseen CRISPR-Cas9 off-target activity. Genome Biol. 21, 290. 10.1186/s13059-020-02206-w 33261648 PMC7706270

[B85] Hossain A. Skalicky M. Brestic M. Maitra S. Alam M. A. Syed M. A. (2021a). Consequences and mitigation strategies of abiotic stresses in wheat (*Triticum aestivum* l.) under the changing climate. Agronomy 11, 241. 10.3390/agronomy11020241

[B86] Hossain C. M. Dutta D. Vichare R. Biswal M. R. Ali K. A. Chakraborty P. (2021b). Silk protein-based nanomaterials in drug delivery and biomedical applications. Biopolym. Nanomater Drug Deliv. Biomed. Appl., 447–463. 10.1016/b978-0-12-820874-8.00024-5

[B87] Hu T. Gu Z. Williams G. R. Strimaite M. Zha J. Zhou Z. (2022). Layered double hydroxide-based nanomaterials for biomedical applications. Chem. Soc. Rev. 51, 6126–6176. 10.1039/d2cs00236a 35792076

[B88] Huang R. Y. Liu Z. H. Weng W. H. Chang C. W. (2021). Magnetic nanocomplexes for gene delivery applications. J. Mater. Chem. B 9, 4267–4286. 10.1039/d0tb02713h 33942822

[B89] Huang C. Zhou W. Wu R. Guan W. Ye N. (2023). Recent advances in nanomaterial-based chemiluminescence probes for biosensing and imaging of reactive oxygen species. Nanomaterials 13, 1726. 10.3390/nano13111726 37299629 PMC10254534

[B90] Hur J. Chung A. J. (2021). Microfluidic and nanofluidic intracellular delivery. Adv. Sci. 8, 2004595. 10.1002/advs.202004595 34096197 PMC8336510

[B91] Ibrahim J. P. Haque S. Bischof R. J. Whittaker A. K. Whittaker M. R. Kaminskas L. M. (2022). Liposomes are poorly absorbed *via* lung lymph after inhaled administration in sheep. Front. Pharmacol. 13, 880448. 10.3389/fphar.2022.880448 35721215 PMC9201389

[B92] Ihnatsyeu-Kachan A. Dzmitruk V. Apartsin E. Krasheninina O. Ionov M. Loznikova S. (2017). Multi-target inhibition of cancer cell growth by SiRNA cocktails and 5-fluorouracil using effective piperidine-terminated phosphorus dendrimers. Colloids Interfaces 1, 6. 10.3390/colloids1010006

[B93] Imlimthan S. Correia A. Figueiredo P. Lintinen K. Balasubramanian V. Airaksinen A. J. (2020). Systematic *in vitro* biocompatibility studies of multimodal cellulose nanocrystal and lignin nanoparticles. J. Biomed. Mater Res. - Part A 108, 770–783. 10.1002/jbm.a.36856 31794149

[B94] Irshad M. A. Sattar S. Nawaz R. Al-Hussain S. A. Rizwan M. Bukhari A. (2023). Enhancing chromium removal and recovery from industrial wastewater using sustainable and efficient nanomaterial: a review. Ecotoxicol. Environ. Saf. 263, 115231. 10.1016/j.ecoenv.2023.115231 37429088

[B95] Jackman J. A. Yoon B. K. Ouyang L. Wang N. Ferhan A. R. Kim J. (2021). Biomimetic nanomaterial strategies for virus targeting: antiviral therapies and vaccines. Adv. Funct. Mater. 31, 2008352. 10.1002/adfm.202008352

[B96] Jahangiri-Manesh A. Mousazadeh M. Taji S. Bahmani A. Zarepour A. Zarrabi A. (2022). Gold nanorods for drug and gene delivery: an overview of recent advancements. Pharmaceutics 14, 664. 10.3390/pharmaceutics14030664 35336038 PMC8951391

[B97] Jain K. K. (2012). Advances in use of functionalized carbon nanotubes for drug design and discovery. Expert Opin. Drug Discov. 7, 1029–1037. 10.1517/17460441.2012.722078 22946637

[B98] Jeong G. J. Castels H. Kang I. Aliya B. Jang Y. C. (2022). Nanomaterial for skeletal muscle regeneration. Tissue Eng. Regen. Med. 19, 253–261. 10.1007/s13770-022-00446-4 35334091 PMC8971233

[B99] Jones C. F. Grainger D. W. (2009). *In vitro* assessments of nanomaterial toxicity. Adv. Drug Deliv. Rev. 61, 438–456. 10.1016/j.addr.2009.03.005 19383522 PMC2763955

[B100] Joun I. Nixdorf S. Deng W. (2022). Advances in lipid-based nanocarriers for breast cancer metastasis treatment. Front. Med. Technol. 4, 893056. 10.3389/fmedt.2022.893056 36062261 PMC9433809

[B101] Judith J. V. Vasudevan N. (2022). Synthesis of nanomaterial from industrial waste and its application in environmental pollutant remediation. Environ. Eng. Res.

[B102] Kabir E. Raza N. Kumar V. Singh J. Tsang Y. F. Lim D. K. (2019). Recent advances in nanomaterial-based human breath analytical technology for clinical diagnosis and the way forward. Chem. 5, 3020–3057. 10.1016/j.chempr.2019.08.004

[B103] Kang Y. Lee Y. Khang D. Hill J. J. (2017). Enhanced anti-inflammatory effect of low dose dexamethasone-carbon nanotube conjugates on human arthritis synovial fibroblasts. Osteoarthr. Cartil. 25, S416. 10.1016/j.joca.2017.02.719

[B104] Kapoor N. Kaisar A. Pratap Singh N. Singh J. Neelesh Kapoor C. Dixit R. (2018). A study to evaluate the effect of silver nanoparticles synthesized by *Sonchus asper* on fenugreek plant. J. Pharmacogn. Phytochem. 7, 1144–1149.

[B105] Karthikeyan C. Varaprasad K. Venugopal S. K. Shakila S. Venkatraman B. R. Sadiku R. (2021). Biocidal (bacterial and cancer cells) activities of chitosan/CuO nanomaterial, synthesized *via* a green process. Carbohydr. Polym. 259, 117762. 10.1016/j.carbpol.2021.117762 33674015

[B106] Katayama T. Tanaka Y. Okabe T. Nakamura H. Fujii W. Kitamoto K. (2016). Development of a genome editing technique using the CRISPR/Cas9 system in the industrial filamentous fungus *Aspergillus oryzae* . Biotechnol. Lett. 38, 637–642. 10.1007/s10529-015-2015-x 26687199

[B107] Kaur B. Kumar S. Kaushik B. K. (2023). Trends, challenges, and advances in optical sensing for pathogenic bacteria detection (PathoBactD). Biosens. Bioelectron. X 14, 100352. 10.1016/j.biosx.2023.100352

[B108] Kausar A. (2021). Polymer dots and derived hybrid nanomaterials: a review. J. Plast. Film. Sheeting 37, 510–528. 10.1177/87560879211010313

[B109] Kavanagh E. W. Green J. J. (2022). Toward gene transfer nanoparticles as therapeutics. Adv. Healthc. Mater. 11, 2102145. 10.1002/adhm.202102145 35006646

[B110] Khaliq N. U. Lee J. Kim J. Kim Y. Yu S. Kim J. (2023). Mesoporous silica nanoparticles as a gene delivery platform for cancer therapy. Pharmaceutics 15, 1432. 10.3390/pharmaceutics15051432 37242674 PMC10224024

[B111] Khan S. H. (2019). Genome-editing technologies: concept, pros, and cons of various genome-editing techniques and bioethical concerns for clinical application. Mol. Ther. Nucleic Acids 16, 326–334. 10.1016/j.omtn.2019.02.027 30965277 PMC6454098

[B112] Khan S. (2021). Recent advancement and innovations in CRISPR/Cas and CRISPR related technologies: a review. Biotechnol. Bioprocess. 2, 01–12. 10.31579/2766-2314/042

[B113] Khan B. A. Nadeem M. A. Iqbal M. Yaqoob N. Javaid M. M. Maqbool R. (2023). Chitosan nanoparticles loaded with mesosulfuron methyl and mesosulfuron methyl + florasulam + MCPA isooctyl to manage weeds of wheat (*Triticum aestivum* L.). Green Process Synth. 12, 20228152. 10.1515/gps-2022-8152

[B315] Khanna K. Ohri P. Bhardwaj R. (2023). Nanotechnology and CRISPR/Cas9 system for sustainable agriculture. Environ. Sci. Pollut. Res. 30, 118049–118064. 10.1007/s11356-023-26482-8 36973619

[B114] Kim J. H. Moon M. J. Kim D. Y. Heo S. H. Jeong Y. Y. (2018). Hyaluronic acid-based nanomaterials for cancer therapy. Polym. (Basel) 10, 1133. 10.3390/polym10101133 30961058 PMC6403826

[B115] Kim J. Y. Kaganovich I. Lee H. C. (2022). Review of the gas breakdown physics and nanomaterial-based ionization gas sensors and their applications. Plasma Sources Sci. Technol. 31, 033001. 10.1088/1361-6595/ac4574

[B116] Kishore K. Pandey A. Wagri N. K. Saxena A. Patel J. Al-Fakih A. (2023). Technological challenges in nanoparticle-modified geopolymer concrete: a comprehensive review on nanomaterial dispersion, characterization techniques and its mechanical properties. Case Stud. Constr. Mater. 19, e02265. 10.1016/j.cscm.2023.e02265

[B117] Kladko D. V. Falchevskaya A. S. Serov N. S. Prilepskii A. Y. (2021). Nanomaterial shape influence on cell behavior. Int. J. Mol. Sci. 22, 5266. 10.3390/ijms22105266 34067696 PMC8156540

[B118] Kopecny D. Vlcko T. (2020). Plant biotechnology: green for good V 2019. N. Biotechnol. 57, 1–3. 10.1016/j.nbt.2020.01.004 32017997

[B119] Kotb A. Abdel-Rahim R. D. Ali A. S. Gomaa H. (2023). Smart nanomaterials based on metals and metal oxides for photocatalytic applications. Adv. Smart Nanomater their Appl., 351–421. 10.1016/b978-0-323-99546-7.00004-5

[B120] Koupaei M. S. Gabris M. A. Hadi J. B. Baradaran R. Aziz M. Karim KJBA (2019). Adsorption and *in vitro* release study of curcumin form polyethyleneglycol functionalized multi walled carbon nanotube: kinetic and isotherm study. DARU. J. Pharm. Sci. 27, 9–20. 10.1007/s40199-018-0232-2 PMC6593009 30554368

[B121] Kozielski K. L. Tzeng S. Y. Hurtado De Mendoza B. A. Green J. J. (2014). Bioreducible cationic polymer-based nanoparticles for efficient and environmentally triggered cytoplasmic siRNA delivery to primary human brain cancer cells. ACS Nano 8, 3232–3241. 10.1021/nn500704t 24673565 PMC4004313

[B122] Krasteva N. Georgieva M. (2022). Promising therapeutic strategies for colorectal cancer treatment based on nanomaterials. Pharmaceutics 14, 1213. 10.3390/pharmaceutics14061213 35745786 PMC9227901

[B123] Kreyling W. G. Semmler-Behnke M. Chaudhry Q. (2010). A complementary definition of nanomaterial. Nano Today 5, 165–168. 10.1016/j.nantod.2010.03.004

[B124] Kuhlbusch T. A. J. Wijnhoven S. W. P. Haase A. (2018). Nanomaterial exposures for worker, consumer and the general public. NanoImpact 10, 11–25. 10.1016/j.impact.2017.11.003

[B125] Kumar A. Choudhary A. Kaur H. Mehta S. Husen A. (2021). Smart nanomaterial and nanocomposite with advanced agrochemical activities. Nanoscale Res. Lett. 16, 156. 10.1186/s11671-021-03612-0 34664133 PMC8523620

[B126] Kushwaha A. Goswami L. Kim B. S. (2022). Nanomaterial-based therapy for wound healing. Nanomaterials 12, 618. 10.3390/nano12040618 35214947 PMC8878029

[B127] Ladu L. (2021). The governance of genome editing techniques for the European bio-based industry. J. Environ. Policy Plan. 23, 165–180. 10.1080/1523908x.2020.1850247

[B128] Lai W. F. Rogach A. L. Wong W. T. (2017). Molecular design of upconversion nanoparticles for gene delivery. Chem. Sci. 8, 7339–7358. 10.1039/c7sc02956j 29163885 PMC5672820

[B129] Lamboro A. Song B. Songnan Y. Han X. Mingguo H. Li X. (2021). Genetic engineering and genome editing techniques in peanut plants. Plant Sci. Today 8, 528–534. 10.14719/pst.2021.8.3.1127

[B130] Lan S. Liu Y. Shi K. Ma D. (2020). Acetal-functionalized Pillar[5]arene: a pH-Responsive and versatile nanomaterial for the delivery of chemotherapeutic agents. ACS Appl. Bio Mater 3, 2325–2333. 10.1021/acsabm.0c00086 35025284

[B131] Lastochkina O. Aliniaeifard S. SeifiKalhor M. Bosacchi M. Maslennikova D. Lubyanova A. (2022). Novel approaches for sustainable horticultural crop production: advances and prospects. Horticulturae 8, 910. 10.3390/horticulturae8100910

[B132] Lee T. Kim S. Kim J. Park S. C. Yoon J. Park C. (2020). Recent advances in biomolecule-nanomaterial heterolayer-based charge storage devices for bioelectronic applications. Mater. (Basel) 13, 3520. 10.3390/ma13163520 32784985 PMC7475838

[B133] Lee J. Kang Y. K. Oh E. Jeong J. Im S. H. Kim D. K. (2022). Nano-assembly of a chemically tailored Cas9 ribonucleoprotein for *in vivo* gene editing and cancer immunotherapy. Chem. Mater 34, 547–561. 10.1021/acs.chemmater.1c02844

[B134] Li J. (2019). Multi-targeting nano-delivery system provides vehicle for *in vivo* CRISPR/Cas9 gene editing. Kexue Tongbao/Chinese Sci. Bull. 64, 2561–2563. 10.1360/tb-2019-0343

[B135] Li M. Zhao H. Ananiev G. E. Musser M. T. Ness K. H. Maglaque D. L. (2017). Establishment of reporter lines for detecting fragile X mental retardation (FMR1) gene reactivation in human neural cells. Stem Cells 35, 158–169. 10.1002/stem.2463 27422057 PMC5195860

[B136] Li Y. Fang H. Zhang T. Wang Y. Qi T. Li B. (2022a). Lipid-mRNA nanoparticles landscape for cancer therapy. Front. Bioeng. Biotechnol. 10, 1053197. 10.3389/fbioe.2022.1053197 36394007 PMC9659646

[B137] Li Y. Xie M. Jones J. B. Zhang Z. Wang Z. Dang T. (2022b). Targeted delivery of DNA topoisomerase inhibitor SN38 to intracranial tumors of glioblastoma using Sub-5 ultrafine iron oxide nanoparticles. Adv. Healthc. Mater 11, 2102816. 10.1002/adhm.202102816 35481625 PMC9308697

[B138] Li T. Zhang L. Lu T. Zhu T. Feng C. Gao N. (2023). Engineered extracellular vesicle-delivered CRISPR/CasRx as a novel RNA editing tool. Adv. Sci. 10, 2206517. 10.1002/advs.202206517 36727818 PMC10074121

[B139] Liang J. Liang K. (2021). Nano-bio-interface engineering of metal-organic frameworks. Nano Today 40, 101256. 10.1016/j.nantod.2021.101256

[B140] Liang J. Zhao X. (2021). Nanomaterial-based delivery vehicles for therapeutic cancer vaccine development. Cancer Biol. Med. 18, 352–371. 10.20892/j.issn.2095-3941.2021.0004 33979069 PMC8185868

[B141] Liang T. Hou J. R. Qu M. Xi J. X. Raj I. (2022a). Application of nanomaterial for enhanced oil recovery. Pet. Sci. 19, 882–899. 10.1016/j.petsci.2021.11.011

[B142] Liang Y. Xu X. Xu L. Iqbal Z. Ouyang K. Zhang H. (2022b). Chondrocyte-specific genomic editing enabled by hybrid exosomes for osteoarthritis treatment. Theranostics 12, 4866–4878. 10.7150/thno.69368 35836795 PMC9274754

[B143] Liang M. Shang L. Yu Y. Jiang Y. Bai Q. Ma J. (2023). Ultrasound activatable microneedles for bilaterally augmented sono-chemodynamic and sonothermal antibacterial therapy. Acta Biomater. 158, 811–826. 10.1016/j.actbio.2022.12.041 36572249

[B316] Lima R. Del Fiol F. S. Balcão V. M. (2019). Prospects for the use of new technologies to combat multidrug-resistant bacteria. Front. Pharmacol. 10. 10.3389/fphar.2019.00692 31293420 PMC6598392

[B144] Lin Y. Mao C. (2011). Bio-inspired supramolecular self-assembly towards soft nanomaterials. Front. Mater. Sci. 5, 247–265. 10.1007/s11706-011-0141-5 21980594 PMC3185360

[B145] Lin L. Wang Q. (2015). Microplasma: a new generation of technology for functional nanomaterial synthesis. Plasma Chem. Plasma Process. 35, 925–962. 10.1007/s11090-015-9640-y

[B146] Lin G. Zhang H. Huang L. (2015). Smart polymeric nanoparticles for cancer gene delivery. Mol. Pharm. 12, 314–321. 10.1021/mp500656v 25531409 PMC4319689

[B147] Lin Z. Deng Q. Fang Q. Li X. Liu X. Wang J. (2022). Black phosphorus nanoparticles for dual therapy of non-small cell lung cancer. J. Drug Target 30, 614–622. 10.1080/1061186x.2022.2032093 35078385

[B148] Liu J. Wang Z. Zhao S. Ding B. (2018). Multifunctional nucleic acid nanostructures for gene therapies. Nano Res. 11, 5017–5027. 10.1007/s12274-018-2093-x

[B149] Liu J. Li R. Yang B. (2020). Carbon dots: a new type of carbon-based nanomaterial with wide applications. ACS Cent. Sci. 6, 2179–2195. 10.1021/acscentsci.0c01306 33376780 PMC7760469

[B150] Liu L. Ma Q. Cao J. Gao Y. Han S. Liang Y. (2021a). Recent progress of graphene oxide-based multifunctional nanomaterials for cancer treatment. Cancer Nanotechnol. 12, 18. 10.1186/s12645-021-00087-7

[B151] Liu M. Y. Hang C. Z. Zhao X. F. Zhu L. Y. Ma R. G. Wang J. C. (2021b). Advance on flexible pressure sensors based on metal and carbonaceous nanomaterial. Nano Energy. 87, 106181. 10.1016/j.nanoen.2021.106181

[B152] Liu J. Li X. Liu L. Bai Q. Sui N. Zhu Z. (2021c). Self-assembled ultrasmall silver nanoclusters on liposome for topical antimicrobial delivery. Colloids Surfaces B Biointerfaces 200, 111618. 10.1016/j.colsurfb.2021.111618 33592456

[B153] Liu Y. Zhu S. Gu Z. Chen C. Zhao Y. (2022a). Toxicity of manufactured nanomaterials. Particuology 69, 31–48. 10.1016/j.partic.2021.11.007

[B154] Liu Y. Song S. Liu S. Zhu X. Wang P. (2022b). Application of nanomaterial in hydrogels related to wound healing. J. Nanomater. 2022, 4656037. 10.1155/2022/4656037

[B155] Liu H. Zhu X. Wei Y. Song C. Wang Y. (2023). Recent advances in targeted gene silencing and cancer therapy by nanoparticle-based delivery systems. Biomed. Pharmacother. 157, 114065. 10.1016/j.biopha.2022.114065 36481408

[B156] Lu X. Zhang M. Li G. Zhang S. Zhang J. Fu X. (2023). Applications and research advances in the delivery of CRISPR/Cas9 systems for the treatment of inherited diseases. Int. J. Mol. Sci. 24, 13202. 10.3390/ijms241713202 37686009 PMC10487642

[B157] Luly K. M. Choi J. Rui Y. Green J. J. Jackson E. M. (2020). Safety considerations for nanoparticle gene delivery in pediatric brain tumors. Nanomedicine 15, 1805–1815. 10.2217/nnm-2020-0110 32698671 PMC7441302

[B158] Lupu A.-R. Popescu T. Stojanović M. (2020). Therapeutic use of inorganic nanomaterials in malignant diseases, 47–87.

[B159] Ma Y. Han X. Quintana Bustamante O. Bessa De Castro R. Zhang K. Zhang P. (2017). Highly efficient genome editing of human hematopoietic stem cells *via* a nano-silicon-blade delivery approach. Integr. Biol. (United Kingdom) 9, 548–554. 10.1039/c7ib00060j 28513735 PMC5598083

[B160] Madl A. K. Teague S. V. Qu Y. Masiel D. Evans J. E. Guo T. (2012). Aerosolization system for experimental inhalation studiesof carbon-based nanomaterials. Aerosol Sci. Technol. 46, 94–107. 10.1080/02786826.2011.605813

[B161] Maity S. Mukherjee R. Banerjee S. (2023). Recent advances and therapeutic strategies using CRISPR genome editing technique for the treatment of cancer. Mol. Biotechnol. 65, 206–226. 10.1007/s12033-022-00550-9 35999480

[B162] Majidi S. Zeinali Sehrig F. Samiei M. Milani M. Abbasi E. Dadashzadeh K. (2016). Magnetic nanoparticles: applications in gene delivery and gene therapy. Artif. Cells, Nanomedicine Biotechnol. 44, 1186–1193. 10.3109/21691401.2015.1014093 25727710

[B163] Makabenta J. M. V. Nabawy A. Li C. H. Schmidt-Malan S. Patel R. Rotello V. M. (2021). Nanomaterial-based therapeutics for antibiotic-resistant bacterial infections. Nat. Rev. Microbiol. 19, 23–36. 10.1038/s41579-020-0420-1 32814862 PMC8559572

[B164] Manikantan V. Sri Varalakshmi G. Sumohan Pillai A. Alexander A. Lucas A. Kathiravan E. (2023). 5-Fluorouracil-loaded designed praseodymium oxide – poly- β-cyclodextrin nanorods for effectively inhibiting breast cancer cells. Inorg. Chem. Commun. 153, 110830. 10.1016/j.inoche.2023.110830

[B165] Manimekala T. Sivasubramanian R. Dharmalingam G. (2022). Nanomaterial-based biosensors using field-effect transistors: a review. J. Electron. Mater. 51, 1950–1973. 10.1007/s11664-022-09492-z 35250154 PMC8881998

[B166] Mashima R. Takada S. (2022). Lipid nanoparticles: a novel gene delivery technique for clinical application. Curr. Issues Mol. Biol. 44, 5013–5027. 10.3390/cimb44100341 36286056 PMC9600891

[B167] McDonald T. O. Siccardi M. Moss D. Liptrott N. Giardiello M. Rannard S. (2015). “The application of nanotechnology to drug delivery in medicine,” in Nanoeng glob approaches to heal saf issues, 173–223.

[B168] Miao L. Guo S. Zhang J. Kim W. Y. Huang L. (2014). Nanoparticles with precise ratiometric co-loading and co-delivery of gemcitabine monophosphate and cisplatin for treatment of bladder cancer. Adv. Funct. Mater 24, 6601–6611. 10.1002/adfm.201401076 25395922 PMC4225819

[B169] Miller J. B. Siegwart D. J. (2018). Design of synthetic materials for intracellular delivery of RNAs: from siRNA-mediated gene silencing to CRISPR/Cas gene editing. Nano Res. 11, 5310–5337. 10.1007/s12274-018-2099-4

[B170] Min S. Kang H. Seo B. Cheong J. Roh C. Hong S. (2021). A review of nanomaterial based scintillators. Energies.

[B171] Mirón-Barroso S. Domènech E. B. Trigueros S. (2021). Nanotechnology-based strategies to overcome current barriers in gene delivery. Int. J. Mol. Sci. 10.3390/ijms22168537 PMC8395193 34445243

[B172] Mohamad F. Alzahrani R. R. Alsaadi A. Alrfaei B. M. Yassin A. E. B. Alkhulaifi M. M. (2023). An explorative review on advanced approaches to overcome bacterial resistance by curbing bacterial biofilm formation. Infect. Drug Resist. 16, 19–49. 10.2147/idr.s380883 36636380 PMC9830422

[B173] Mohammadinejad R. Dehshahri A. Sagar Madamsetty V. Zahmatkeshan M. Tavakol S. Makvandi P. (2020). *In vivo* gene delivery mediated by non-viral vectors for cancer therapy. J. Control Release 325, 249–275. 10.1016/j.jconrel.2020.06.038 32634464 PMC7334939

[B174] Mohsin M. Ishaq T. Bhatti I. A. Maryam Jilani, A. Melaibari A. A. (2023). Semiconductor nanomaterial photocatalysts for water-splitting hydrogen production: the Holy grail of converting solar energy to fuel. Nanomaterials 13, 546. 10.3390/nano13030546 36770508 PMC9921879

[B175] Moradi P. Hasanzadeh A. Radmanesh F. Rajai Daryasarei S. Hosseini E. S. Kiani J. (2022). Smart arginine-equipped polycationic nanoparticles for p/CRISPR delivery into cells. Nanotechnology 33, 075104. 10.1088/1361-6528/ac357a 34727527

[B176] Morán M. C. Carazo J. Busquets M. A. (2018). Dual responsive gelatin-based nanoparticles for enhanced 5-fluorouracil efficiency. Colloids Surfaces B Biointerfaces 172, 646–654. 10.1016/j.colsurfb.2018.09.027 30243218

[B177] Mostafavi E. Zare H. (2022). Carbon-based nanomaterials in gene therapy. OpenNano 7, 100062. 10.1016/j.onano.2022.100062

[B178] Mushtaq M. Sakina A. Wani S. H. Shikari A. B. Tripathi P. Zaid A. (2019). Harnessing genome editing techniques to engineer disease resistance in plants. Front. Plant Sci. 10, 550. 10.3389/fpls.2019.00550 31134108 PMC6514154

[B179] Nafees M. Liaqut W. Ali S. Shafique M. A. (2013). Synthesis of ZnO/Al:ZnO nanomaterial: structural and band gap variation in ZnO nanomaterial by Al doping. Appl. Nanosci. 3, 49–55. 10.1007/s13204-012-0067-y

[B317] Naik B. J. Shimoga G. Kim S. C. Manjulatha M. Subramanyam Reddy C. Palem P. R. (2022). CRISPR/Cas9 and Nanotechnology Pertinence in Agricultural Crop Refinement. Front. Plant Sci. 13. 10.3389/fpls.2022.843575 35463432 PMC9024397

[B180] Nain A. Sangili A. Hu S. R. Chen C. H. Chen Y. L. Chang H. T. (2022). Recent progress in nanomaterial-functionalized membranes for removal of pollutants. iScience 25, 104616. 10.1016/j.isci.2022.104616 35789839 PMC9250028

[B181] Naskar A. Kim K. S. (2020). Recent advances in nanomaterial-based wound-healing therapeutics. Pharmaceutics 12, 499. 10.3390/pharmaceutics12060499 32486142 PMC7356512

[B182] Niazian M. Molaahmad Nalousi A. Azadi P. Ma’mani L. Chandler S. F. (2021). Perspectives on new opportunities for nano-enabled strategies for gene delivery to plants using nanoporous materials. Planta 254, 83. 10.1007/s00425-021-03734-w 34559312

[B183] Nocito G. Calabrese G. Forte S. Petralia S. Puglisi C. Campolo M. (2021). Carbon dots as promising tools for cancer diagnosis and therapy. Cancers (Basel) 13, 1991. 10.3390/cancers13091991 33919096 PMC8122497

[B184] Ohyanagi T. Nagahori N. Shimawaki K. Hinou H. Yamashita T. Sasaki A. (2011). Importance of sialic acid residues illuminated by live animal imaging using phosphorylcholine self-assembled monolayer-coated quantum dots. J. Am. Chem. Soc. 133, 12507–12517. 10.1021/ja111201c 21740000

[B185] Ojha A. Jaiswal S. Bharti P. Mishra S. K. (2023). Nanoparticles and nanomaterials-based recent approaches in upgraded targeting and management of cancer: a review. Cancers (Basel) 15, 162. 10.3390/cancers15010162 36612158 PMC9817889

[B186] Opanasopit P. Apirakaramwong A. Ngawhirunpat T. Rojanarata T. Ruktanonchai U. (2008). Development and characterization of pectinate micro/nanoparticles for gene delivery. AAPS PharmSciTech 9, 67–74. 10.1208/s12249-007-9007-7 18446463 PMC2976884

[B187] Padayachee J. Singh M. (2020). Therapeutic applications of CRISPR/Cas9 in breast cancer and delivery potential of gold nanomaterials. Nanobiomedicine. 10.1177/1849543520983196 PMC7768851 33488814

[B188] Pan H. Yu Y. Li L. Liu B. Liu Y. (2021). Fabrication and characterization of taurine functionalized graphene oxide with 5-Fluorouracil as anticancer drug delivery systems. Nanoscale Res. Lett. 16, 84. 10.1186/s11671-021-03541-y 33983544 PMC8119561

[B189] Peng C. Huang Y. Zheng J. (2020). Renal clearable nanocarriers: overcoming the physiological barriers for precise drug delivery and clearance. J. Control Release 322, 64–80. 10.1016/j.jconrel.2020.03.020 32194171 PMC8696951

[B190] Pezoa S. Alfano R. Hazi N. (2022). Gene editing/gene therapies: evaluation of transfection reagents for high titer lentivirus production in the icellis nano bioreactor. Cytotherapy 24, S142. 10.1016/s1465-3249(22)00376-0

[B191] Pillai G. (2014). Nanomedicines for cancer therapy: an update of FDA approved and those under various stages of development. SOJ Pharm. Pharm. Sci. 1. 10.15226/2374-6866/1/1/00109

[B192] Pillai G. Ceballos-Coronel M. L. (2013). “Science and technology of the emerging nanomedicines in cancer therapy,” in A primer for physicians and pharmacists, 1. SAGE Open Med. 10.1177/2050312113513759 PMC4687778 26770691

[B193] Pillai G. Cox A. Yuen L. (2018). The science and technology of cancer Theranostic nanomedicines: a primer for Clinicians and pharmacists. SOJ Pharm. Pharm. Sci. 5, 1–17. 10.15226/2374-6866/5/2/00178

[B194] Pissuwan D. Niidome T. Cortie M. B. (2011). The forthcoming applications of gold nanoparticles in drug and gene delivery systems. J. Control. Release. 149, 65–71. 10.1016/j.jconrel.2009.12.006 20004222

[B195] Pokhrel N. Vabbina P. K. Pala N. (2016). Sonochemistry: science and engineering. Ultrason. Sonochem. 29, 104–128. 10.1016/j.ultsonch.2015.07.023 26584990

[B196] Popova J. Bets V. Kozhevnikova E. (2023). Perspectives in genome-editing techniques for livestock. Animals 13, 2580. 10.3390/ani13162580 37627370 PMC10452040

[B197] Prakasha D. G. Sudharani MVVNL Kumar K. G. Chamkha A. J. (2023). Comparative study of hybrid (graphene/magnesium oxide) and ternary hybrid (graphene/zirconium oxide/magnesium oxide) nanomaterials over a moving plate. Int. Commun. Heat. Mass Transf. 140, 106557. 10.1016/j.icheatmasstransfer.2022.106557

[B198] Priyadarsini S. Mohanty S. Mukherjee S. Basu S. Mishra M. (2018). Graphene and graphene oxide as nanomaterials for medicine and biology application. J. Nanostructure Chem. 8, 123–137. 10.1007/s40097-018-0265-6

[B199] Qamar H. Saeed A. Owais M. Hussain T. Hussain K. Rahman A. U. (2021). CuO bionanocomposite with enhanced stability and antibacterial activity against extended-spectrum beta-lactamase strains. Mater. (Basel) 14, 6336. 10.3390/ma14216336 34771863 PMC8585137

[B200] Qiao H. H. Zhang Q. H. Ming D. (2021). CRISPR/Cas9 system and its application in tumor therapy. Prog. Biochem. Biophys., 570–579.

[B201] Quader S. Kataoka K. (2017). Nanomaterial-enabled cancer therapy. Mol. Ther. 25, 1501–1513. 10.1016/j.ymthe.2017.04.026 28532763 PMC5498831

[B202] Rahman M. M. Islam M. Roy R. Younis H. AlNahyan M. Younes H. (2022). Carbon nanomaterial-based lubricants: review of recent developments. Lubricants 10, 281. 10.3390/lubricants10110281

[B203] Rao C. N. R. Sood A. K. Subrahmanyam K. S. Govindaraj A. (2009). Graphene: the new two-dimensional nanomaterial. Angew. Chem. - Int. Ed. 48, 7752–7777. 10.1002/anie.200901678 19784976

[B204] Rawtani D. Tharmavaram M. Pandey G. Hussain C. M. (2019). Functionalized nanomaterial for forensic sample analysis. Trac. - Trends Anal. Chem. 120, 115661. 10.1016/j.trac.2019.115661

[B205] Raya I. Kzar H. H. Mahmoud Z. H. Al Ayub Ahmed A. Ibatova A. Z. Kianfar E. (2022). A review of gas sensors based on carbon nanomaterial. Carbon Lett. 32, 339–364. 10.1007/s42823-021-00276-9

[B206] Raza S. H. Zaman U. Iftikhar M. Shafique O. (2021). An experimental evidence on eco-friendly advertisement appeals and intention to use bio-nanomaterial plastics: institutional collectivism and performance orientation as moderators. Int. J. Environ. Res. Public Health 18, 1–16. 10.3390/ijerph18020791 33477726 PMC7832387

[B207] Rezk M. Y. Sharma J. Gartia M. R. (2020). Nanomaterial-based CO_2_ sensors. Nanomaterials 10, 1–18. 10.3390/nano10112251 33202957 PMC7697554

[B208] Rizeq B. R. Younes N. N. Rasool K. Nasrallah G. K. (2019). Synthesis, bioapplications, and toxicity evaluation of chitosan-based nanoparticles. Int. J. Mol. Sci. 20, 5776. 10.3390/ijms20225776 31744157 PMC6888098

[B209] Roma-rodrigues C. Rivas-garcía L. Baptista P. V. Fernandes A. R. (2020). Gene therapy in cancer treatment: why go nano? Pharmaceutics 12, 233. 10.3390/pharmaceutics12030233 32151052 PMC7150812

[B210] Rong L. Lei Q. Zhang X. Z. (2020). Recent advances on peptide-based theranostic nanomaterials. VIEW 1, 20200050. 10.1002/viw.20200050

[B211] Rouatbi N. Mun Lim Y. Grant V. Miguel Costa P. Pollard S. M. Wang J. T.-W. (2019). CRISPR/Cas9 gene editing of brain cancer stem cells using lipid-based nano-delivery. Neuro Oncol. 21, iv7. 10.1093/neuonc/noz167.029

[B212] Ryu J. Y. Won E. J. Lee H. A. R. Kim J. H. Hui E. Kim H. P. (2020). Ultrasound-activated particles as CRISPR/Cas9 delivery system for androgenic alopecia therapy. Biomaterials, 232. 10.1016/j.biomaterials.2019.119736 31901692

[B213] Sahel D. K. Vora L. K. Saraswat A. Sharma S. Monpara J. D’Souza A. A. (2023). CRISPR/Cas9 genome editing for tissue-specific *in vivo* targeting: nanomaterials and translational perspective. Adv. Sci. 10, 2305072. 10.1002/advs.202305072 37667835 PMC10477853

[B214] Salekdeh P. R. Ma’mani L. Tavakkoly-Bazzaz J. Mousavi H. Modarressi M. H. Salekdeh G. H. (2021). Bi-functionalized aminoguanidine-PEGylated periodic mesoporous organosilica nanoparticles: a promising nanocarrier for delivery of Cas9-sgRNA ribonucleoproteine. J. Nanobiotechnology 19, 95. 10.1186/s12951-021-00838-z 33789675 PMC8011395

[B215] Salman A. Kantor A. McClements M. E. Marfany G. Trigueros S. MacLaren R. E. (2022). Non-viral delivery of CRISPR/Cas cargo to the retina using nanoparticles: current possibilities, challenges, and limitations. Pharmaceutics 14, 1842. 10.3390/pharmaceutics14091842 36145593 PMC9503525

[B216] Samarasinghe R. M. Gibbons J. Kanwar R. K. Kanwar J. R. (2012). Nanotechnology based platforms for survivin targeted drug discovery. Expert Opin. Drug Discov. 7, 1083–1092. 10.1517/17460441.2012.719869 22950742

[B217] Sami H. Maparu A. K. Kumar A. Sivakumar S. (2012). Generic delivery of payload of nanoparticles intracellularly *via* hybrid polymer capsules for bioimaging applications. PLoS One 7, e36195. 10.1371/journal.pone.0036195 22649489 PMC3359331

[B218] Santana I. Jeon S. J. Kim H. I. Islam M. R. Castillo C. Garcia G. F. H. (2022). Targeted carbon nanostructures for chemical and gene delivery to plant chloroplasts. ACS Nano 16, 12156–12173. 10.1021/acsnano.2c02714 35943045

[B219] Saritha G. N. G. Anju T. Kumar A. (2022). Nanotechnology - big impact: how nanotechnology is changing the future of agriculture? J. Agric. Food Res. 10, 100457. 10.1016/j.jafr.2022.100457

[B220] Satta A. Esquirol L. Ebert B. E. (2023). Current metabolic engineering strategies for photosynthetic bioproduction in Cyanobacteria. Microorganisms 11, 455. 10.3390/microorganisms11020455 36838420 PMC9964548

[B221] Sethuram L. Thomas J. Mukherjee A. Chandrasekaran N. (2022). A review on contemporary nanomaterial-based therapeutics for the treatment of diabetic foot ulcers (DFUs) with special reference to the Indian scenario. Nanoscale Adv. 4, 2367–2398. 10.1039/d1na00859e 36134136 PMC9418054

[B222] Shafee A. Shahraki M. S. Taleghani A. H. Nam N. D. Tlili I. (2021). Analysis of nanomaterial flow among two circular tubes in the presence of magnetic force. J. Therm. Anal. Calorim. 144, 993–1002. 10.1007/s10973-020-09555-5

[B223] Shao Y. Yang G. Lin J. Fan X. Guo Y. Zhu W. (2021). Shining light on chiral inorganic nanomaterials for biological issues. Theranostics 11, 9262–9295. 10.7150/thno.64511 34646370 PMC8490512

[B224] Sharma P. Lew T. T. S. (2022). Principles of nanoparticle design for genome editing in plants. Front. Genome 4, 846624. 10.3389/fgeed.2022.846624 35330692 PMC8940305

[B225] Sharma B. Soni U. Afonso L. O. B. Cahill D. M. (2022). Nanomaterial doping: chemistry and strategies for agricultural applications. ACS Agric. Sci. Technol. 2, 240–257. 10.1021/acsagscitech.1c00273

[B226] Sharshar M. M. Samak N. A. Ambreen S. Hao X. Mu T. Maarouf M. (2020). Improving confirmed nanometric sulfur bioproduction using engineered Thioalkalivibrio versutus. Bioresour. Technol. 317, 124018. 10.1016/j.biortech.2020.124018 32836035

[B227] Sheikhzadeh E. Beni V. Zourob M. (2021). Nanomaterial application in bio/sensors for the detection of infectious diseases. Talanta 230, 122026. 10.1016/j.talanta.2020.122026 33934756 PMC7854185

[B228] Shen Y. Cohen J. L. Nicoloro S. M. Kelly M. Yenilmez B. Henriques F. (2018a). CRISPR-delivery particles targeting nuclear receptor–interacting protein 1 (Nrip1) in adipose cells to enhance energy expenditure. J. Biol. Chem. 293, 17291–17305. 10.1074/jbc.ra118.004554 30190322 PMC6222111

[B229] Shen L. Li B. Qiao Y. (2018b). Fe3O4 nanoparticles in targeted drug/gene delivery systems. Mater. (Basel) 11, 324. 10.3390/ma11020324 29473914 PMC5849021

[B230] Shen J. Lu Z. Wang J. Hao Q. Ji W. Wu Y. (2021). Traceable nano-biohybrid complexes by one-step synthesis as CRISPR-Chem vectors for neurodegenerative diseases synergistic treatment. Adv. Mater. 33, 2101993. 10.1002/adma.202101993 34046943

[B231] Shen T. Zhang Y. Mei L. Zhang X. B. Zhu G. (2022). Single-stranded circular DNA theranostics. Theranostics 12, 35–47. 10.7150/thno.66466 34987632 PMC8690921

[B232] Shende P. Trivedi R. (2021). 3D printed bioconstructs: regenerative modulation for genetic expression. Stem Cell Rev. Rep. 17, 1239–1250. 10.1007/s12015-021-10120-2 33454852 PMC7811392

[B233] Shi S. Chen F. Ehlerding E. B. Cai W. (2014). Surface engineering of graphene-based nanomaterials for biomedical applications. Bioconjug. Chem. 25, 1609–1619. 10.1021/bc500332c 25117569 PMC4166029

[B234] Shin M. Lim J. An J. Yoon J. Choi J. W. (2023). Nanomaterial-based biohybrid hydrogel in bioelectronics. Nano Converg. 10, 8. 10.1186/s40580-023-00357-7 36763293 PMC9918666

[B235] Shishparenok A. N. Furman V. V. Zhdanov D. D. (2023). DNA-based nanomaterials as drug delivery platforms for increasing the effect of drugs in tumors. Cancers (Basel) 15, 2151. 10.3390/cancers15072151 37046816 PMC10093432

[B236] Singh V. Nair S. P. N. Aradhyam G. K. (2013). Chemistry of conjugation to gold nanoparticles affects G-protein activity differently. J. Nanobiotechnology 11, 7. 10.1186/1477-3155-11-7 23510390 PMC3614441

[B237] Sohaebuddin S. K. Thevenot P. T. Baker D. Eaton J. W. Tang L. (2010). Nanomaterial cytotoxicity is composition, size, and cell type dependent. Part Fibre Toxicol. 7, 22. 10.1186/1743-8977-7-22 20727197 PMC2936333

[B238] Song N. Li S. Lv Z. Ding X. Li F. Yang D. (2023). Engineering CRISPR/Cas-based nanosystems for therapeutics, diagnosis and bioimaging. Chin. Chem. Lett. 34, 108134. 10.1016/j.cclet.2023.108134

[B239] Soroush A. Rice D. Rahaman M. S. Perreault F. (2016). Graphene-based materials in health and environment. Graphene based mater heal. Environ, 287–322.

[B240] Souri M. Chiani M. Farhangi A. Mehrabi M. R. Nourouzian D. Raahemifar K. (2022). Anti-COVID-19 nanomaterials: directions to improve prevention, diagnosis, and treatment. Nanomaterials 12, 783. 10.3390/nano12050783 35269270 PMC8912597

[B241] Stater E. P. Sonay A. Y. Hart C. Grimm J. (2021). The ancillary effects of nanoparticles and their implications for nanomedicine. Nat. Nanotechnol. 16, 1180–1194. 10.1038/s41565-021-01017-9 34759355 PMC9031277

[B242] Stern S. T. Adiseshaiah P. P. Crist R. M. (2012). Autophagy and lysosomal dysfunction as emerging mechanisms of nanomaterial toxicity. Part. Fibre Toxicol. 9, 20. 10.1186/1743-8977-9-20 22697169 PMC3441384

[B243] Štimac A. Tokić M. Ljubetič A. Vuletić T. Šekutor M. Požar J. (2019). Functional self-assembled nanovesicles based on β-cyclodextrin, liposomes and adamantyl guanidines as potential nonviral gene delivery vectors. Org. Biomol. Chem. 17, 4640–4651. 10.1039/c9ob00488b 31020307

[B244] Su F. Huang Z. Guo Y. Jiao R. Zi L. Chen J. (2016). From random mutagenesis to precise genome editing: the development and evolution of genome editing techniques in Drosophila. Yi Chuan 38, 17–27. 10.16288/j.yczz.15-337 26787520

[B245] Sun Q. Hong Z. Zhang C. Wang L. Han Z. Ma D. (2023). Immune checkpoint therapy for solid tumours: clinical dilemmas and future trends. Signal Transduct. Target. Ther. 8, 320. 10.1038/s41392-023-01522-4 37635168 PMC10460796

[B246] Suri S. S. Fenniri H. Singh B. (2007). Nanotechnology-based drug delivery systems. J. Occup. Med. Toxicol. 2, 16. 10.1186/1745-6673-2-16 18053152 PMC2222591

[B247] Szczeszak A. Ekner-Grzyb A. Runowski M. Mrówczyńska L. Grzyb T. Lis S. (2015). Synthesis, photophysical analysis, and *in vitro* cytotoxicity assessment of the multifunctional (magnetic and luminescent) core@shell nanomaterial based on lanthanide-doped orthovanadates. J. Nanoparticle Res. 17, 143. 10.1007/s11051-015-2950-4

[B248] Tang Z. Wu H. Cort J. R. Buchko G. W. Zhang Y. Shao Y. (2010). Constraint of DNA on functionalized graphene improves its biostability and specificity. Small 6, 1205–1209. 10.1002/smll.201000024 20461727

[B249] Tariq Z. Qadeer M. I. Anjum I. Hano C. Anjum S. (2023). Thalassemia and nanotheragnostics: advanced approaches for diagnosis and treatment. Biosensors 13, 450. 10.3390/bios13040450 37185525 PMC10136341

[B250] Tay A. Melosh N. (2019). Transfection with nanostructure electro-injection is minimally perturbative. Adv. Ther. 2, 1900133. 10.1002/adtp.201900133 37448511 PMC10343936

[B251] Teleanu R. I. Gherasim O. Gherasim T. G. Grumezescu V. Grumezescu A. M. Teleanu D. M. (2019). Nanomaterial-based approaches for neural regeneration. Pharmaceutics 11, 266. 10.3390/pharmaceutics11060266 31181719 PMC6630326

[B252] Tian H. Chen J. Chen X. (2013). Nanoparticles for gene delivery. Small 9, 2034–2044. 10.1002/smll.201202485 23630123

[B253] Tiwari P. K. Ko T. H. Dubey R. Chouhan M. Tsai L. W. Singh H. N. (2023). CRISPR/Cas9 as a therapeutic tool for triple negative breast cancer: from bench to clinics. Front. Mol. Biosci. 10, 1214489. 10.3389/fmolb.2023.1214489 37469704 PMC10352522

[B254] Tsoi K. M. Macparland S. A. Ma X. Z. Spetzler V. N. Echeverri J. Ouyang B. (2016). Mechanism of hard-nanomaterial clearance by the liver. Nat. Mater. 15, 1212–1221. 10.1038/nmat4718 27525571 PMC5132626

[B255] Turcheniuk K. Mochalin V. N. (2017a). Biomedical applications of nanodiamond. Nanotechnology. (Review). 10.1088/1361-6528/aa6ae4 28368852

[B256] Turcheniuk K. Mochalin V. N. (2017b). To cite this article: k turcheniuk and Vadym N Mochalin. Nanotechnology 28, 252001. 10.1088/1361-6528/aa6ae4 28368852

[B257] Unnisa A. Greig N. Kamal M. (2023). Nanotechnology-based gene therapy as a credible tool in the treatment of Alzheimer’s disease. Neural Regen. Res. 18, 2127–2133. 10.4103/1673-5374.369096 37056119 PMC10328264

[B258] Vaidh S. Parekh D. Patel D. Vishwakarma G. S. (2022). Leachate treatment potential of nanomaterial based assemblies: a systematic review on recent development. Water Sci. Technol. 85, 3285–3300. 10.2166/wst.2022.168 35704411

[B259] van de Wiel C. C. M. Schaart J. G. Lotz L. A. P. Smulders M. J. M. (2017). New traits in crops produced by genome editing techniques based on deletions. Plant Biotechnol. Rep. 11, 1–8. 10.1007/s11816-017-0425-z 28386301 PMC5360818

[B260] Vance M. E. Kuiken T. Vejerano E. P. McGinnis S. P. Hochella M. F. Hull D. R. (2015). Nanotechnology in the real world: redeveloping the nanomaterial consumer products inventory. Beilstein J. Nanotechnol. 6, 1769–1780. 10.3762/bjnano.6.181 26425429 PMC4578396

[B261] Vélez M. A. Perotti M. C. Santiago L. Gennaro A. M. Hynes E. (2017). Nutrient delivery (nanotechnology in the agri-Food industry). Elsevier.

[B262] Velluto D. Bojadzic D. De Toni T. Buchwald P. Tomei A. A. (2021). Drug-integrating amphiphilic nanomaterial assemblies: 1. spatiotemporal control of cyclosporine delivery and activity using nanomicelles and nanofibrils. J. Control Release 329, 955–970. 10.1016/j.jconrel.2020.10.026 33086102 PMC7904645

[B263] Vimbela G. V. Ngo S. M. Fraze C. Yang L. Stout D. A. (2017). Antibacterial properties and toxicity from metallic nanomaterials. Int. J. Nanomedicine. 12, 3941–3965. 10.2147/ijn.s134526 28579779 PMC5449158

[B320] Wang B. Lu J. Zhang X. Hu R. Ma H. (2025). Advances in nanomaterial-mediated CRISPR/Cas delivery: from lipid nanoparticles to vesicle-derived systems. Front. Bioeng. Biotechnol. 13, 1669104. 10.3389/fbioe.2025.1669104 PMC12872781 41659011

[B331] Wang K. Xu B. F. Lei C. Y. Nie Z. (2021). Advances in the Integration of Nucleic Acid Nanotechnology into CRISPR-Cas System. J. Anal. Test. 5, 130–141. 10.1007/s41664-021-00180-1

[B264] Wang Y. Guo L. (2016). Nanomaterial-enabled neural stimulation. Front. Neurosci. 10, 69. 10.3389/fnins.2016.00069 27013938 PMC4779906

[B265] Wang Y. Rajala A. Rajala R. (2015). Lipid nanoparticles for ocular gene delivery. J. Funct. Biomater. 6, 379–394. 10.3390/jfb6020379 26062170 PMC4493518

[B266] Wang L. Huang J. Chen H. Wu H. Xu Y. Li Y. (2017). Exerting enhanced permeability and retention effect driven delivery by ultrafine iron oxide nanoparticles with T1-T2 switchable magnetic resonance imaging contrast. ACS Nano. 11, 4582–4592. 10.1021/acsnano.7b00038 28426929 PMC5701890

[B267] Wang Y. Sun S. Zhang Z. Shi D. (2018). Nanomaterials for cancer precision medicine. Adv. Mater. 30, 1705660. 10.1002/adma.201705660 29504159

[B268] Wang Z. H. Liu J. M. Li C. Y. Wang D. Lv H. Lv S. W. (2019). Bacterial biofilm bioinspired persistent luminescence nanoparticles with gut-oriented drug delivery for colorectal cancer imaging and chemotherapy. ACS Appl. Mater Interfaces 11, 36409–36419. 10.1021/acsami.9b12853 31525949

[B269] Wei S. Shao X. Liu Y. Xiong B. Cui P. Liu Z. (2022). Genome editing of PD-L1 mediated by nucleobase-modified polyamidoamine for cancer immunotherapy. J. Mater Chem. B 10, 1291–1300. 10.1039/d1tb02688g 35141737

[B270] Wei Hu S. Ding T. Tang H. Guo H. Cui W. Shu Y. (2023). Nanobiomaterial vectors for improving gene editing and gene therapy. Mater. Today. 66, 114–136. 10.1016/j.mattod.2023.04.011

[B271] Wong L. W. Fahimizadeh M. Tan J. B. L. (2023). Cellulose and cellulose derivatives-based nanosystems as therapeutic platform. Polym. Nanosyst. Theranostic Nanosyst. 1, 113–147. 10.1016/b978-0-323-85656-0.00022-x

[B272] Wu K. Li H. Cui X. Feng R. Chen W. Jiang Y. (2022a). Mutagenesis and resistance development of bacteria challenged by silver nanoparticles. Antimicrob. Agents Chemother. 66, e00628-22. 10.1128/aac.00628-22 36094196 PMC9578424

[B273] Wu Z. Huo X. Yang T. Liu K. Wu T. Feng Z. (2022b). CRISPR/Cas9-3NLS/sgHMGA2@PDA nanosystem is the potential efficient gene editing therapy for gastric cancer with HMGA2 high expression. Front. Oncol. 12, 978533. 10.3389/fonc.2022.978533 36119467 PMC9479195

[B274] Xavier Mendes A. Moraes Silva S. O’Connell C. D. Duchi S. Quigley A. F. Kapsa R. M. I. (2021). Enhanced electroactivity, mechanical properties, and printability through the addition of graphene oxide to photo-cross-linkable gelatin methacryloyl hydrogel. ACS Biomater. Sci. Eng. 7, 2279–2295. 10.1021/acsbiomaterials.0c01734 33956434

[B275] Xi S. Yang Y. G. Suo J. Sun T. (2022). Research progress on gene editing based on nano-drug delivery vectors for tumor therapy. Front. Bioeng. Biotechnol. 10, 873369. 10.3389/fbioe.2022.873369 35419357 PMC8996155

[B276] Xie J. Gong L. Zhu S. Yong Y. Gu Z. Zhao Y. (2019). Emerging strategies of nanomaterial-mediated tumor radiosensitization. Adv. Mater. 31, 1802244. 10.1002/adma.201802244 30156333

[B277] Xie S. Ai L. Cui C. Fu T. Cheng X. Qu F. (2021). Functional aptamer-embedded nanomaterials for diagnostics and therapeutics. ACS Appl. Mater. Interfaces. 13, 9542–9560. 10.1021/acsami.0c19562 33595277

[B278] Xin T. Cheng L. Zhou C. Zhao Y. Hu Z. Wu X. (2022). *In-Vivo* induced CAR-T cell for the potential breakthrough to overcome the barriers of current CAR-T cell therapy. Front. Oncol. 12, 809754. 10.3389/fonc.2022.809754 35223491 PMC8866962

[B279] Xu P. Liang F. (2020). Nanomaterial-based tumor photothermal immunotherapy. Int. J. Nanomedicine. 15, 9159–9180. 10.2147/ijn.s249252 33244232 PMC7684030

[B280] Xu X. Liu Y. Yang Y. Wu J. Cao M. Sun L. (2022). One-pot synthesis of functional peptide-modified gold nanoparticles for gene delivery. Colloids Surfaces A Physicochem Eng. Asp., 640.

[B319] Xu X. Liu C. Wang Y. Koivisto O. Zhou J. Shu Y. (2021). Nanotechnology-based delivery of CRISPR/Cas9 for cancer treatment. Adv. Drug Deliv. Rev. 176. 10.1016/j.addr.2021.113891 34324887

[B281] Yadav K. Sahu K. K. Gnanakani S. P. Sure P. Vijayalakshmi R. Sundar V. D. Sharma V. (2023a). Biomedical applications of nanomaterials in the advancement of nucleic acid therapy: mechanistic challenges, delivery strategies, and therapeutic applications. Int. J. Biol. Macromol. 241, 124582. 10.1016/j.ijbiomac.2023.124582 37116843

[B282] Yadav M. R. Kumar S. Lal M. K. Kumar D. Kumar R. Yadav R. K. (2023b). Mechanistic understanding of leakage and consequences and recent technological advances in improving nitrogen use efficiency in cereals. Agronomy 13, 527. 10.3390/agronomy13020527

[B283] Yadav P. Shah K. Kansara K. Kumar A. Rawal R. Bhatia D. (2023c). Tissue-derived primary cell type dictates the endocytic uptake route of carbon quantum dots and *in vivo* uptake. ACS Appl. Bio Mater 6, 1629–1638. 10.1021/acsabm.3c00072 36976263

[B284] Yan B. Liang Y. (2022). New therapeutics for extracellular vesicles: delivering CRISPR for cancer treatment. Int. J. Mol. Sci. 23, 15758. 10.3390/ijms232415758 36555398 PMC9779094

[B285] Yan X. Sedykh A. Wang W. Yan B. Zhu H. (2020). Construction of a web-based nanomaterial database by big data curation and modeling friendly nanostructure annotations. Nat. Commun. 11, 2519. 10.1038/s41467-020-16413-3 32433469 PMC7239871

[B286] Yang K. Feng L. Liu Z. (2015). The advancing uses of nano-graphene in drug delivery. Expert Opin. Drug Deliv. 12, 601–612. 10.1517/17425247.2015.978760 25466364

[B287] Yang T. C. Chang C. Y. Yarmishyn A. A. Mao Y. S. Yang Y. P. Wang M. L. (2020a). Carboxylated nanodiamond-mediated CRISPR-Cas9 delivery of human retinoschisis mutation into human iPSCs and mouse retina. Acta Biomater. 101, 484–494. 10.1016/j.actbio.2019.10.037 31672582

[B288] Yang Y. Zhang M. Song H. Yu C. (2020b). Silica-based nanoparticles for biomedical applications: from nanocarriers to biomodulators. Acc. Chem. Res. 53, 1545–1556. 10.1021/acs.accounts.0c00280 32667182

[B289] Yang P. Lee A. Y. T. Xue J. Chou S. J. Lee C. Tseng P. (2022). Nano-vectors for CRISPR/Cas9-mediated genome editing. Nano Today 44, 101482. 10.1016/j.nantod.2022.101482 PMC13341159 42416481

[B290] Yang J. Yang K. Du S. Luo W. Wang C. Liu H. (2023). Bioorthogonal reaction-mediated tumor-selective delivery of CRISPR/Cas9 system for dual-targeted cancer immunotherapy. Angew Chem Int Ed Engl 62, e202306863. 37485554 10.1002/anie.202306863

[B291] Yao S. Swetha P. Zhu Y. (2018). Nanomaterial-enabled wearable sensors for healthcare. Adv. Healthc. Mater. 7, 1700889. 10.1002/adhm.201700889 29193793

[B292] Yaqoob A. A. Parveen T. Umar K. Ibrahim M. N. M. (2020). Role of nanomaterials in the treatment of wastewater: a review. Water (Switzerland).

[B293] Yin F. Hu K. Chen Y. Yu M. Wang D. Wang Q. (2017). SiRNA delivery with PEGylated graphene oxide nan osheets for combined photothermal and genetherapy for pancreatic cancer. Theranostics 7, 1133–1148. 10.7150/thno.17841 28435453 PMC5399581

[B294] Ying Y. Ying W. Li Q. Meng D. Ren G. Yan R. (2017). Recent advances of nanomaterial-based membrane for water purification. Appl. Mater. Today. 7, 144–158. 10.1016/j.apmt.2017.02.010

[B295] Yu G. Zhao R. Wu D. Zhang F. Shao L. Zhou J. (2016a). Pillar[5]arene-based amphiphilic supramolecular brush copolymers: fabrication, controllable self-assembly and application in self-imaging targeted drug delivery. Polym. Chem. 7, 6178–6188. 10.1039/c6py01402j 27795740 PMC5084091

[B296] Yu G. Vaidya A. Sun D. Lu Z. R. (2016b). Nanomaterials for treating ocular diseases. Methods Pharmacol. Toxicol., 369–388. 10.1007/978-1-4939-3121-7_19

[B297] Yuan B. Wang Y. Zong C. Sang L. Chen S. Liu C. (2023). Modeling study for predicting altered cellular activity induced by nanomaterials based on Dlk1-Dio3 gene expression and structural relationships. Chemosphere 335, 139090. 10.1016/j.chemosphere.2023.139090 37268226

[B298] Zhai L. M. Zhao Y. Xiao R. L. Zhang S. Q. Tian B. H. Li X. X. (2022). Nuclear-targeted carbon quantum dot mediated CRISPR/Cas9 delivery for fluorescence visualization and efficient editing. Nanoscale 14, 14645–14660. 10.1039/d2nr04281a 36165075

[B299] Zhang W. Chen Q. Wu F. Dai J. Ding D. Wu J. (2021a). Peptide-based nanomaterials for gene therapy. Nanoscale Adv. 3, 302–310. 10.1039/d0na00899k

[B300] Zhang Y. Hou D. Wang Z. Cai N. Au C. (2021b). Nanomaterial-based dual-emission ratiometric fluorescent sensors for biosensing and cell imaging. Polym. (Basel) 13, 2540. 10.3390/polym13152540 34372142 PMC8348892

[B301] Zhang L. Ma X. J. N. Fei Y. Y. Han H. T. Xu J. Cheng L. (2022a). Stem cell therapy in liver regeneration: focus on mesenchymal stem cells and induced pluripotent stem cells. Pharmacol. Ther. 232, 108004. 10.1016/j.pharmthera.2021.108004 34597754

[B302] Zhang A. Fang J. Wang J. Xie X. Chen H. J. He G. (2022b). Interrogation on the cellular nano-interface and biosafety of repeated nano-electroporation by nanostraw System. Biosensors 12, 522. 10.3390/bios12070522 35884325 PMC9313307

[B303] Zhao J. Yin F. Ji L. Wang C. Shi C. Liu X. (2020). Development of a tau-targeted drug delivery system using a multifunctional nanoscale metal-organic framework for alzheimer’s disease therapy. ACS Appl. Mater Interfaces 12, 44447–44458. 10.1021/acsami.0c11064 32897042

[B304] Zhao H. Zhang L. Zhou Y. Zhang Q. (2023). Recycling of Fe3O4 nanomaterial from coal fly ash as catalyst to develop green and sustainable bio-electro fenton: characterization, optimization, and performance. J. Environ. Chem. Eng. 11, 110678. 10.1016/j.jece.2023.110678

[B305] Zhen S. Liu Y. Lu J. Tuo X. Yang X. Chen H. (2020). Human papillomavirus oncogene manipulation using clustered regularly interspersed short palindromic Repeats/Cas9 delivered by pH-sensitive cationic liposomes. Hum. Gene Ther. 31, 309–324. 10.1089/hum.2019.312 31973584

[B306] Zheng M. Pan M. Zhang W. Lin H. Wu S. Lu C. (2021). Poly(α-L-lysine)-based nanomaterials for versatile biomedical applications: current advances and perspectives. Bioact. Mater. 6, 1878–1909. 10.1016/j.bioactmat.2020.12.001 33364529 PMC7744653

[B307] Zhi H. Zhou S. Pan W. Shang Y. Zeng Z. Zhang H. (2022). The promising nanovectors for gene delivery in plant genome engineering. Int. J. Mol. Sci. 23, 8501. 10.3390/ijms23158501 35955636 PMC9368765

[B330] Zhou S. Li Y. Wu Q. Gong C. (2024). Nanotechnology-based CRISPR/Cas9 delivery system for genome editing in cancer treatment. MedComm - Biomater. Appl. 3. 10.1002/mba2.70

[B308] Zhou Y. Quan G. Wu Q. Zhang X. Niu B. Wu B. (2018). Mesoporous silica nanoparticles for drug and gene delivery. Acta Pharm. Sin. B 8, 165–177. 10.1016/j.apsb.2018.01.007 29719777 PMC5926503

[B309] Zhou Y. Mo M. Luo D. Yang Y. Hu J. Ye C. (2022). Evolutionary trend analysis of research on 5-ALA delivery and Theranostic applications based on a scientometrics study. Pharmaceutics 14, 1477. 10.3390/pharmaceutics14071477 35890373 PMC9320574

[B310] Zhu Y. Shen R. Vuong I. Reynolds R. A. Shears M. J. Yao Z. C. (2022). Multi-step screening of DNA/lipid nanoparticles and co-delivery with siRNA to enhance and prolong gene expression. Nat. Commun. 13, 4282. 10.1038/s41467-022-31993-y 35879315 PMC9310361

[B311] Zia R. Nawaz M. S. Siddique M. J. Hakim S. Imran A. (2021). Plant survival under drought stress: implications, adaptive responses, and integrated rhizosphere management strategy for stress mitigation. Microbiol. Res. 242, 126626. 10.1016/j.micres.2020.126626 33189069

[B312] Živojević K. Mladenović M. Djisalov M. Mundzic M. Ruiz-Hernandez E. Gadjanski I. (2021). Advanced mesoporous silica nanocarriers in cancer theranostics and gene editing applications. J. Control. Release. 337, 193–211. 10.1016/j.jconrel.2021.07.029 34293320

[B313] Zych A. O. Bajor M. Zagozdzon R. (2018). Application of genome editing techniques in immunology. Arch. Immunol. Ther. Exp. Warsz. 66, 289–298. 10.1007/s00005-018-0504-z 29344676 PMC6061149

